# NK cells contribute to the resolution of experimental malaria-associated acute respiratory distress syndrome after antimalarial treatment

**DOI:** 10.3389/fimmu.2024.1433904

**Published:** 2024-09-17

**Authors:** Emilie Pollenus, Hendrik Possemiers, Sofie Knoops, Fran Prenen, Leen Vandermosten, Thao-Thy Pham, Laura Buysrogge, Patrick Matthys, Philippe E. Van den Steen

**Affiliations:** ^1^ Laboratory of Immunoparasitology, Department of Microbiology, Immunology & Transplantation, Rega Institute for Medical Research, KU Leuven, Leuven, Belgium; ^2^ Clinical Immunology Unit, Department of Clinical Sciences, Institute of Tropical Medicine Antwerp, Antwerp, Belgium; ^3^ Laboratory of Immunobiology, Department of Microbiology, Immunology & Transplantation, Rega Institute for Medical Research, KU Leuven, Leuven, Belgium

**Keywords:** malaria, inflammation, resolution, natural killer cells, immunology, parasitology

## Abstract

In both humans and mice, natural killer (NK) cells are important lymphocytes of the innate immune system. They are often considered pro-inflammatory effector cells but may also have a regulatory or pro-resolving function by switching their cytokine profile towards the production of anti-inflammatory cytokines, including interleukin-10 (IL-10) and transforming growth factor-β, and by killing pro-inflammatory immune cells. Here, the role of NK cells in the resolution of malaria lung pathology was studied. Malaria complications, such as malaria-associated acute respiratory distress syndrome (MA-ARDS), are often lethal despite the rapid and efficient killing of *Plasmodium* parasites with antimalarial drugs. Hence, studying the resolution and healing mechanisms involved in the recovery from these complications could be useful to develop adjunctive treatments. Treatment of *Plasmodium berghei NK65*-infected C57BL/6 mice with a combination of artesunate and chloroquine starting at the appearance of symptoms was used as a model to study the resolution of MA-ARDS. The role of NK cells was studied using anti-NK1.1 depletion antibodies and NK cell-deficient mice. Using both methods, NK cells were found to be dispensable in the development of MA-ARDS, as shown previously. In contrast, NK cells were crucial in the initiation of resolution upon antimalarial treatment, as survival was significantly decreased in the absence of NK cells. Considerably increased IL-10 expression by NK cells suggested an anti-inflammatory and pro-resolving phenotype. Despite the increase in *Il10* expression in the NK cells, inhibition of the IL-10/IL-10R axis using anti-IL10R antibodies had no effect on the resolution for MA-ARDS, suggesting that the pro-resolving effect of NK cells cannot solely be attributed to their IL-10 production. In conclusion, NK cells contribute to the resolution of experimental MA-ARDS.

## 1 Introduction

In both humans and mice, natural killer (NK) cells are important lymphocytes of the innate immune system and are considered pro-inflammatory effector cells (1). NK cells mediate cytotoxicity against dysfunctional cells, for example after damage or infection, and produce pro-inflammatory cytokines, e.g. interferon-γ (IFN-γ) and tumor necrosis factor-α (TNF-α), and chemokines, such as CC chemokine ligand 5 (CCL5). A variety of activating and inhibitory receptors are expressed on the NK cell surface to scan cell surfaces of potential target cells, for example for a lack of major histocompatibility complex class I (MHCI) on damaged or infected cells. In order to activate NK cells during malaria, direct cell-cell contact with other cells via, for example, intercellular adhesion molecule-1 (ICAM-1), and the presence of interleukin (IL)-2, IL-12 and IL-18 in the environment are needed (2–4). In contrast to their pro-inflammatory role, NK cells also have a regulatory or pro-resolving function. NK cells can switch to the secretion of IL-10 and transforming growth factor-β (TGF-β), and thereby dampen immune responses (5, 6). IL-10 production by NK cells could prevent immunopathology, as was found in mouse models for cerebral malaria, sepsis and cytomegalovirus (7–9). This IL-10 production typically occurs during systemic infections and is IL-12-dependent (10). In addition, NK cells also have an immunoregulatory role by inducing apoptosis of pro-inflammatory immune cells as was shown both *in vitro* and *in vivo* (11–16).

*Plasmodium* parasites cause malaria, a global disease with 249 million clinical cases and 608 000 deaths worldwide in 2022 (17). The majority of infections remain asymptomatic or result in mild febrile disease, because of the development of semi-immunity in adults who get infected regularly (18). However, some infections can lead to life-threatening complications, such as cerebral malaria (CM), malaria-associated acute respiratory distress syndrome (MA-ARDS), severe malarial anemia, malaria-associated acute kidney injury and metabolic complications (19, 20). Mainly adults living in endemic regions or non-immune individuals travelling to those regions develop MA-ARDS, with mortality rates up to 80%. MA-ARDS develops upon excessive pulmonary inflammation, resulting in alveolar-capillary membrane disruption (21, 22). Subsequently, this causes pulmonary edema, microhemorrhages and eventually lethal hypoxemia. This shows that while the activation of the immune system is crucial to attempt to kill the parasite and limit its further replication and dissemination in the human body, an overreaction may be rather detrimental by causing severe immunopathology.

Since NK cells are important producers of IFN-γ and are able to mediate antibody-dependent cellular cytotoxicity (ADCC), they may contribute to protection against parasitemia in malaria patients (23–25). However, excessive production of pro-inflammatory cytokines, such as IFN-γ, but also TNF-α and IL-12, in response to malaria infection, can cause severe immunopathology. In mouse studies, contradicting roles of NK cells in malaria have been described. Several studies using anti-asialo GM1 antibodies to deplete NK cells report that NK cells are critical for sporozoite-induced immunity (4, 26–31). Using the same depletion method, Hansen et al. indicated a role of NK cells in the development of CM (32). Importantly, the anti-asialo GM1 antibody is often used to deplete NK cells, but it may also deplete T cells, basophils, monocytes and other leukocytes (33–36). In contrast, other studies using either anti-asialo GM1 antibodies or the more specific anti-NK1.1 depletion antibodies, could not find a role for NK cells in anti-parasitic immunity (27, 29, 37–40) and in malaria-associated pathology (41–44).

Around 15% of patients with severe malaria succumb to the complication, despite the very rapid and efficient clearance of parasites with artemisinin-based combination therapies (45). The lethality in patients, despite the rapid parasite killing by artemisinin derivatives, indicates the need to study the recovery, also known as resolution, from these malarial complications during antimalarial treatment, in order to find additional pro-resolving treatments that can be used in combination with the already existing antimalarial drugs.

In general, resolution of inflammation aims to dampen the inflammatory response and to restore cell and tissue function (46–48). In the literature, it has been described that general resolution is initiated by apoptosis of pro-inflammatory leukocytes, which are then removed by efferocytosis by macrophages (47). This causes macrophages to switch from the pro-inflammatory M1 to a more reparative M2-like phenotype (49–51). After the initial step limiting the inflammation, wound healing mechanisms remove debris and induce tissue function restoration. Specialized pro-resolving lipid mediators (SPMs), for example lipoxins and resolvins, promote different aspects of the resolution process (52, 53). However, definite data that these mechanisms also contribute to resolution of malaria pathology is lacking.

Until today, resolution remains poorly investigated in the context of malaria. A recent study showed the critical importance of lymphatic clearance of vasogenic edema from the brain during experimental cerebral malaria (54). Some studies describe pro-resolution effects of exogenous molecules in malaria. Lipoxin A4, a member of the SPM family, was found to protect against CM and MA-ARDS (55–57). Treatment of *Pb*ANKA-infected C57BL/6 mice with Lipoxin A4 or 15-epi-lipoxin A4 increased survival and protected against both lung and brain pathology. In addition, anti-inflammatory cytokines, e.g. IL-10, were found to protect against malaria-associated immunopathology (58, 59), but may in parallel aggravate infection by inhibiting anti-parasitic pro-inflammatory responses (60, 61). IL-33 administration together with antimalarial treatment promotes the recovery from CM (62), while contradicting results on the effect of IL-33 alone exist (62–65). IL-15 complex (IL-15C; IL-15 receptor α-Fc fusion protein bound to IL-15) administration was found to promote NK cell-derived IL-10 production resulting in protection from CM (7). However, endogenous mediators of resolution of malaria complications have not been studied thoroughly.

Previously, an experimental model to study MA-ARDS resolution was optimized, by treatment of *Plasmodium berghei* NK65 (*Pb*NK65)-infected C57BL/6 mice with antimalarial drugs. With this model, we indicated a dispensable role for CC chemokine receptor type 2 (CCR2)^+^ monocytes (66). In the current study, the function of NK cells in the resolution of MA-ARDS was investigated. By using anti-NK1.1 depletion antibodies and Natural cytotoxicity triggering receptor 1-improved Cre recombinase (Ncr1-iCre) ROSA-diphteria toxin A (ROSA-DTA) mice in this experimental model, we demonstrate a role for NK cells in the recovery from MA-ARDS. While NK cells were not involved in the development of MA-ARDS, as shown previously (44), our data indicate that NK cells contributed to the initiation of resolution upon antimalarial treatment. These NK cells drastically increased their IL-10 production, corroborating their regulatory/pro-resolving phenotype in the resolution of the malarial lung complication. However, global inhibition of the IL-10/IL-10R axis using anti-IL10R antibodies did not affect the resolution of MA-ARDS upon antimalarial treatment.

## 2 Materials and methods

### 2.1 Mice

C57BL/6 mice (7-8 weeks old) were purchased from Janvier Labs (Le Genest-Saint-Isle, France). SPF Ncr1-iCre ROSA-DTA mice (7-9 weeks old) were bred in the animal facility of the Rega Institute for Medical Research, KU Leuven (Leuven, Belgium). Ncr1-iCre ROSA-DTA mice were kindly gifted by Bart Lambrecht (VIB, University of Ghent, Belgium) with approval of Eric Vivier (Aix-Marseille University, France) (67, 68). Ncr1-iCre^Tg/WT^ ROSA-DTA^Tg/WT^ mice were used as NK cell-deficient mice and their littermates (Ncr1-iCre^Tg/WT^ ROSA-DTA^WT/WT^, Ncr1-iCre^WT/WT^ ROSA-DTA^Tg/WT^ and Ncr1-iCre^WT/WT^ ROSA-DTA^WT/WT^) were used as the appropriate non-deficient controls. Individually ventilated cages in a SPF facility were used to house the mice and they received *ad libitum* high energy food (Ssniff Spezialdiäte GMBH, Soest, Germany) and water, supplemented with 0.422 mg/ml 4-amino-benzoic acid (PABA; Sigma-Aldrich, Bornem, Belgium) after infection. All experiments were performed at the KU Leuven according to the regulations of the European Union (directive 2010/63/EU) and the Belgian Royal Decree of 29 May 2013, and were approved by the Animal Ethics Committee of the KU Leuven (License LA1210186, project P049/2018 and P084/2020, Belgium). Mice were euthanised by intraperitoneal (i.p.) injection of dolethal (Vétoquinol, Aarstelaar, Belgium; 200 mg/ml). Murine blood samples were obtained by cardiac puncture in heparinized (LEO, Pharma, Lier, Belgium) syringes and broncho-alveolar lavage fluids (BALF) were collected before the transcardial perfusion and processed as described previously (66). Lungs and spleen were kept for analysis using flow cytometry or reverse transcriptase-quantitative real time polymerase chain reaction (RT-qPCR).

### 2.2 Genotyping

DNA was isolated from a piece of the tail or ear snippet of Ncr1-iCre ROSA-DTA mice after overnight digestion in NID buffer (50mM KCl, 1mM MgCl_2_, 10 mM Tris-HCl, 0.1 g/ml Gelatine, 0.45% NP40 and 0.45% Tween 20 at pH 8.3) with Proteinase K (0.7 mg/ml, Sigma-Aldrich) at 56°C followed by heat inactivation for 15 min at 94°C. PCR and gel electrophoresis were performed to confirm the genotypes. To determine the percentage of C57BL/6J background of the Ncr1-iCre ROSA-DTA mice, background strain characterization was performed using genome-wide SNP analysis on ear genomic DNA from the 6 original Ncr1-iCre ROSA-DTA mice (Mouse Genome Scanning panel of 2050 SNPs, Taconic, Rensselaer, NY, USA) (**Supplementary Table 1**). Characterisation confirmed the C57BL/6 background (99.22-99.59%)

### 2.3 Parasite infection and clinical scoring

Mice were infected with 10^4^ *Pb*NK65 [Edinburgh strain (69, 70)]-infected red blood cells by i.p. injection. In each experiment, non-infected controls with the same sex and age were included. The severity of disease was evaluated daily from 6 days post infection (dpi) onwards based on body weight, parasitemia and clinical score, as described previously (66).

### 2.4 Treatments

Mice received antimalarial drugs where indicated. Artesunate (ART, 10 mg/kg in 0.9% NaCl with 0.1% NaHCO_3_; Sigma-Aldrich) was combined with chloroquine diphosphate salt (CQ, 30 mg/kg in 0.9% NaCl; Sigma-Aldrich) and i.p. injected daily, starting at 8 dpi until 12 dpi. NK cells were depleted by i.p. injection of 500 µg of InVivoPlus anti-mouse NK1.1 (clone PK136, mouse IgG2a, BioXCell, Lebanon, NH, USA) in Dulbecco’s Phosphate Buffered Saline (DPBS; Lonza, Breda, The Netherlands) at 6 and 9 dpi in case of dissection at 12 dpi, or only at 6 dpi in case of dissection at 9 dpi. DPBS was used as control. IL-10 receptor (IL-10R) was blocked via i.p. injection of 300 µg of InVivoMab anti-mouse IL-10R (clone 1B1.3A, rat IgG1, BioXCell) or InVivoMab IgG1 isotype control (rat anti-HRP, clone HRPN, BioXCell) in DPBS at 8 and 10 dpi.

### 2.5 Determination of lung pathology

Lung pathology was measured based on the weight of the non-perfused left lung, and the protein and IgM concentrations in the BALF. The protein concentration in the BALF supernatant was determined using Bradford assay (Bio-Rad, Hercules, CA, USA). The IgM concentration in the BALF fluid was measured with ELISA, according to the manufacturer’s protocol (Jackson Immunoresearch, Newmarket, UK). Using a Bürker chamber, the number of RBCs and white blood cells (WBCs) in the BALF pellet was counted as a marker for hemorrhages and leukocyte infiltration, respectively.

### 2.6 Measurement of glucose in plasma

Glucose level in the plasma samples was measured with the OneTouch Verio glucometer (LifeScan, Zurich, Switzerland).

### 2.7 Isolation of splenic cells

Splenic cells were isolated as described previously (66). In short, spleens were collected in PBS + 2% fetal calf serum (FCS, Gibco, Borgloon, Belgium) at 4°C. Single cells were obtained after mashing the spleen through a 70 µm nylon cell strainer (VWR, Leuven, Belgium) followed by RBC lysis. Live cells were counted using trypan blue (VWR) in a Bürker chamber.

### 2.8 Isolation of pulmonary cells

Lung cells were isolated, as described previously, using two different protocols, as indicated in the respective figure legends (66). In Protocol 1, lungs were collected in HEPES buffer and homogenized in the gentleMACS™ Dissociator according to the manufacturer’s instructions (MACS Miltenyi Biotec) followed by incubation for 30 min at 37°C in digestion medium containing 2 mg/ml collagenase D (Sigma-Aldrich) and 0.04 mg/ml DNase I (Sigma-Aldrich). After a second processing in the gentle MACS Dissociator, cells were passed through a 70 µm nylon cell strainer. Leukocytes were isolated using a Percoll gradient (40% and 72% Percoll). Live cells were counted using trypan blue in a Bürker chamber.

In Protocol 2, lungs were collected in RPMI buffer (RPMI glutamax + 5% FCS + 1% Penicillin/streptomycin) with 0.1% beta-mercaptoethanol at room temperature (RT). Lungs were first minced with scissors and then incubated for 30 min at 37°C in digestion medium containing 2 mg/ml collagenase D and 0.1 mg/ml DNase I. Afterwards, tissue chunks were minced using a needle and syringe and a second incubation at 37°C for 15 min was performed with fresh digestion medium. Lung tissue was again minced by passing through a syringe and centrifuged. The cell pellet was resuspended using 10 mM EDTA and further diluted in PBS + 2% FCS. RBC lysis was performed, and the cells were passed through a 70 µm nylon cell strainer. Live cells were counted in trypan blue in a Bürker chamber.

### 2.9 Staining and flow cytometry of leukocytes

1.5-3 million cells per sample were washed with PBS. Cells were stained with Zombie Aqua (1/1000; Biolegend, San Diego, CA, USA) or Zombie UV (1/1000; Biolegend) to check viability, combined with Mice Fc block (MACS Miltenyi Biotec), in the dark for 15 min at RT. After washing twice with PBS + 2% FCS + 2 mM EDTA, the cells were incubated with a mixture of monoclonal antibodies (**Supplementary Table 2**) dissolved in PBS with Brilliant stain buffer (BD Biosciences; Erembodegem, Belgium) for 20 min at 4°C in the dark. In the myeloid cell panel, lymphocytes were excluded using a dumpgate (positive for the lineage-specific markers CD3, CD19 and NK1.1 in BV650). Cells were washed and fixated using PBS + 0.4% formaldehyde.

As indicated in **Supplementary Table 2**, per sample, 100 000 or 200 000 live single cells were analyzed with a BD Fortessa X-20 Flow cytometer (BD Biosciences). FlowJo v10 software (FlowJo LLC, Ashland, OR, USA) was used for data analysis according to gating strategies in **Supplementary Figure 1** and as described previously (66). The frequency of a population within total live cells was multiplied by the number of live cells counted in the Bürker chamber, to calculate the absolute number of that population.

### 2.10 Fluorescence-activated sorting of NK cells

Pulmonary cells were stained with Viability dye and fluorescently labelled antibodies as described above. Next, 250 000 live NK cells (gated as CD3^-^ NK1.1^+^ DX5^+^, **Supplementary Figure 2**) were sorted using the BD FACSAria III sorter or BD FACSAria Fusion sorter (BD Biosciences). Afterwards, NK cells were centrifuged, resuspended in lysis buffer (RLT buffer + β-mercaptoethanol) and stored at -80°C until RNA extraction.

### 2.11 Determination of mRNA expression levels

mRNA expression levels were quantified in the left lungs and in sorted NK cells with RT-qPCR. RNA was extracted from the left lung or from sorted NK cells using the Qiagen’s RNeasy Mini Kit or Micro kit (Qiagen, Venlo, The Netherlands), respectively, according to the manufacturer’s protocol. The RNA concentration was determined by measuring absorbance at 260 nm using the CLARIOstar microplate reader with the LVis plate (BMG Labtech, Ortenberg, Germany). cDNA was synthesized using the High-Capacity cDNA Reverse Transcription Kit (Applied Biosystems, Life Technologies). The TaqMan^®^ Fast Universal PCR master mix (Applied Biosystems) was used for the detection of the targeted gene in combination with specific primers (**Supplementary Table 3**). The relative mRNA expression was calculated using the 2^-ΔΔCt^ method, which reflects the fold change in gene expression relative to the mean of the uninfected controls and normalized to the 18S housekeeping gene.

### 2.12 Statistical analysis

The GraphPad PRISM software (GraphPad, San Diego, California, USA) was used for statistical analysis. The non-parametric Mann-Whitney U test followed by the Holm-Bonferroni correction was used. The comparisons that were made to calculate significances are indicated in the figure legends. P-values were indicated as follows: *p<0.05, **p<0.01, ***p<0.001. Median in each group was indicated by a horizontal black line. Statistical differences compared to the appropriate uninfected control group are indicated with asterisk above the individual data sets and horizontal lines with asterisk on top indicate significant differences between groups.

## 3 Results

### 3.1 NK cell depletion hampers the resolution initiation of experimental MA-ARDS with antimalarial drugs

**Figure 1:**
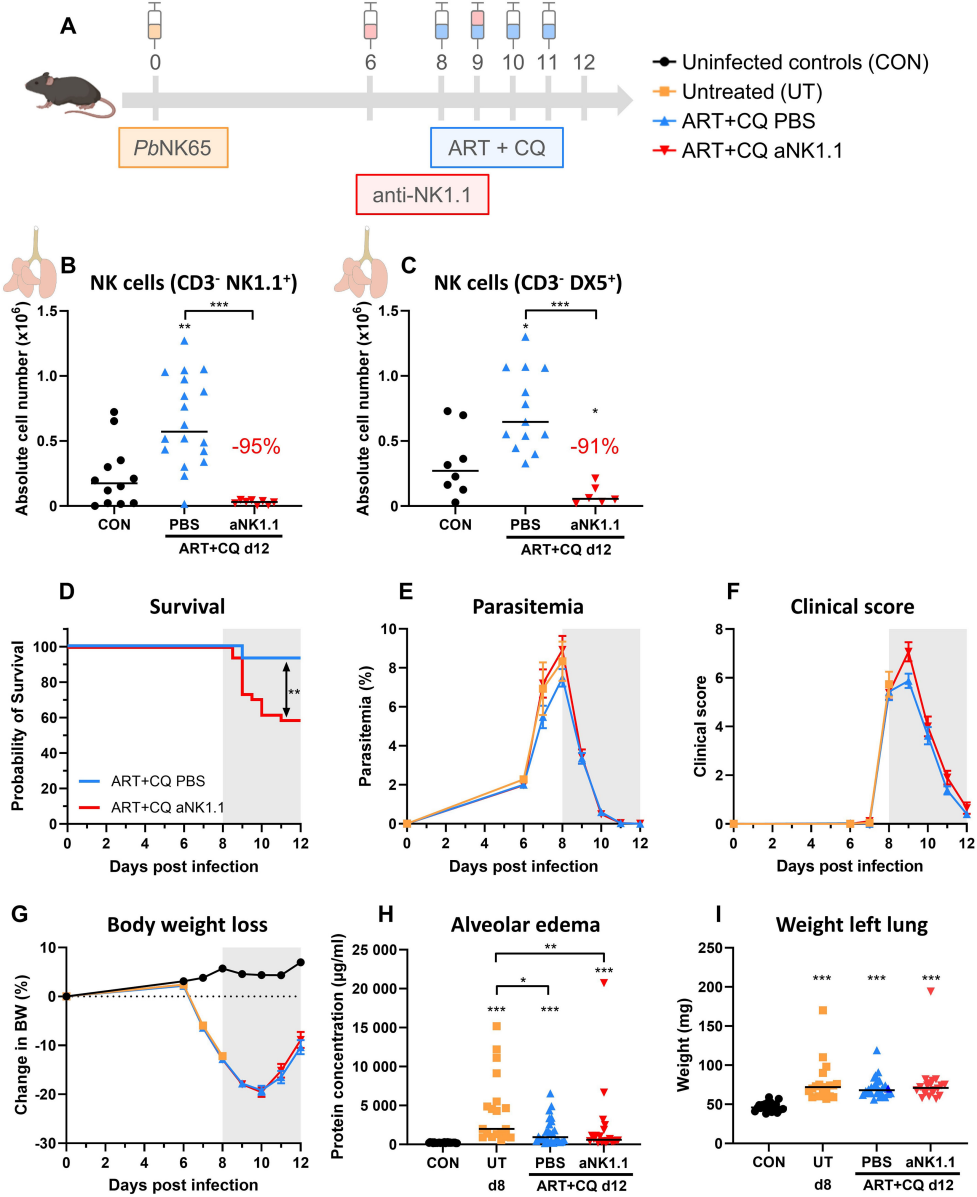
NK cell depletion hampers the initiation of antimalarial treatment-induced resolution of experimental MA-ARDS. C57BL/6 mice were infected with *Pb*NK65. Daily treatment from 8 until 12 dpi with 10 mg/kg artesunate + 30 mg/kg chloroquine (ART+CQ). At 6 and 9 dpi, mice received 500 µg of anti-NK1.1 (PK136) depletion antibodies or PBS. Mice were dissected at 12 dpi. Pulmonary cells were isolated according to protocol 1 and flow cytometry was performed. **(A)** Schematic representation of the timing of infection and treatments in the mouse model. **(B, C)** Number of NK cells present in the lungs gated as CD45^+^ CD3^-^ NK1.1^+^ **(B)** or as CD45^+^ CD3^-^ DX5^+^ **(C)**. Data from three **(B)** or two **(C)** experiments. Each symbol represents data of an individual mouse. n = 8-12 for CON, n = 13-18 for ART+CQ PBS and n = 6-8 for ART+CQ anti-NK1.1 (aNK1.1) group. **(D)** Survival until 12 dpi. Data from five experiments. n = 29 for ART+CQ PBS, n = 34 for ART+CQ aNK1.1. **(E)** Parasitemia was determined daily starting at 6 dpi using Giemsa-stained blood smears. **(F)** Clinical score was monitored daily starting at 6 dpi. **(G)** Body weight loss was calculated compared to 0 dpi starting at 6 dpi. **(E-G)** Data from five experiments. Data are represented as means ± SEM. n = 19-20 for UT, n = 32-35 for ART+CQ PBS, n = 20-44 for ART+CQ aNK1.1 with the highest number in each group indicating the number of mice at the start of the experiment and the lowest number in each group indicating the number of mice remaining at the end of each experiment due to death of the mice. **(H)** Level of alveolar edema was determined based on protein concentration in the BALF. **(I)** Unperfused left lung was weighed as another marker for lung edema. **(H, I)** Data from five experiments. Each symbol represents data of an individual mouse. n = 20-28 for CON, n = 19 for UT, n = 26-27 for ART+CQ PBS, n = 20 for ART+CQ aNK1.1. **(B-I)** The non-parametric Mann-Whitney U test followed by the Holm-Bonferroni correction was used to determine significance between all groups. P-values were indicated as follows: *p<0.05, **p<0.01, ***p<0.001. Median in each group was indicated by a horizontal black line, unless indicated otherwise. Statistical differences compared to the uninfected control group are indicated with asterisk above the individual data sets and horizontal lines with asterisk on top indicate significant differences between groups.

In this study, we used the experimental MA-ARDS model with C57BL/6 mice infected with *Pb*NK65. These mice develop the first symptoms of MA-ARDS at 8 dpi (**Figure 1**). Without antimalarial treatment, these mice die around 10 days post infection. Recently, we showed that NK cell depletion did not affect the development of MA-ARDS (44). To investigate the resolution process in these mice, daily antimalarial treatment with a combination of artesunate and chloroquine (ART+CQ) is started on 8 dpi in order to kill the parasite. This results in the resolution of the pulmonary inflammation and edema and in survival in the majority of the mice, as described in more detail previously (66). To study the role of NK cells in this recovery process, NK cells were depleted by administration of 500 µg of anti-NK1.1 depletion antibodies in PBS at 6 and 9 dpi (**Figure 1A**). Detailed follow-up of the disease course was performed till 12 dpi (disease resolution phase), when mice were dissected for further analysis. NK cells in the lungs were gated both as CD3^-^ NK1.1^+^ (**Figure 1B**) and as CD3^-^ DX5^+^ (**Figure 1C**) to exclude misinterpretation of the depletion efficiency by competition between the depletion anti-NK1.1 and detection anti-NK1.1 antibodies (gating strategy shown in **Supplementary Figure 1**). At 12 dpi, during disease resolution and tissue repairment, the number of NK cells (CD45^+^ CD3^-^ NK1.1^+^ or CD45^+^ CD3^-^ DX5^+^) present in the lungs was increased compared to uninfected control mice (**Figures 1B, C**). Both gating strategies demonstrated a decrease in pulmonary NK cells with more than 90% at 12 dpi after injection with the depletion anti-NK1.1 antibodies (**Figures 1B, C**). Importantly, in the absence of NK cells, a significant decrease in survival (< 60% in comparison to 90%) upon antimalarial treatment was observed, suggesting that NK cells are important for the resolution of MA-ARDS (**Figure 1D**). However, NK-cell depleted mice that did survive until 12 dpi, recovered in a similar manner compared to the non-depleted mice, as evident from similar decrease in parasitemia (**Figure 1E**) and clinical score (**Figure 1F**), and a comparable recovery of the body weight loss (**Figure 1G**). Also, lung pathology, measured by the level of alveolar edema (**Figure 1H**) and weight of the left lung (**Figure 1I**) was similar between the NK cell-depleted and non-depleted mice. These findings suggest that NK cells may be important in the initiation of resolution upon antimalarial treatment but might be dispensable in later phases of the resolution process.

### 3.2 NK cell depletion does not affect other lymphoid and myeloid cell populations in lungs nor spleen of antimalarial-treated *Pb*NK65-infected C57BL/6 mice

**Figure 2:**
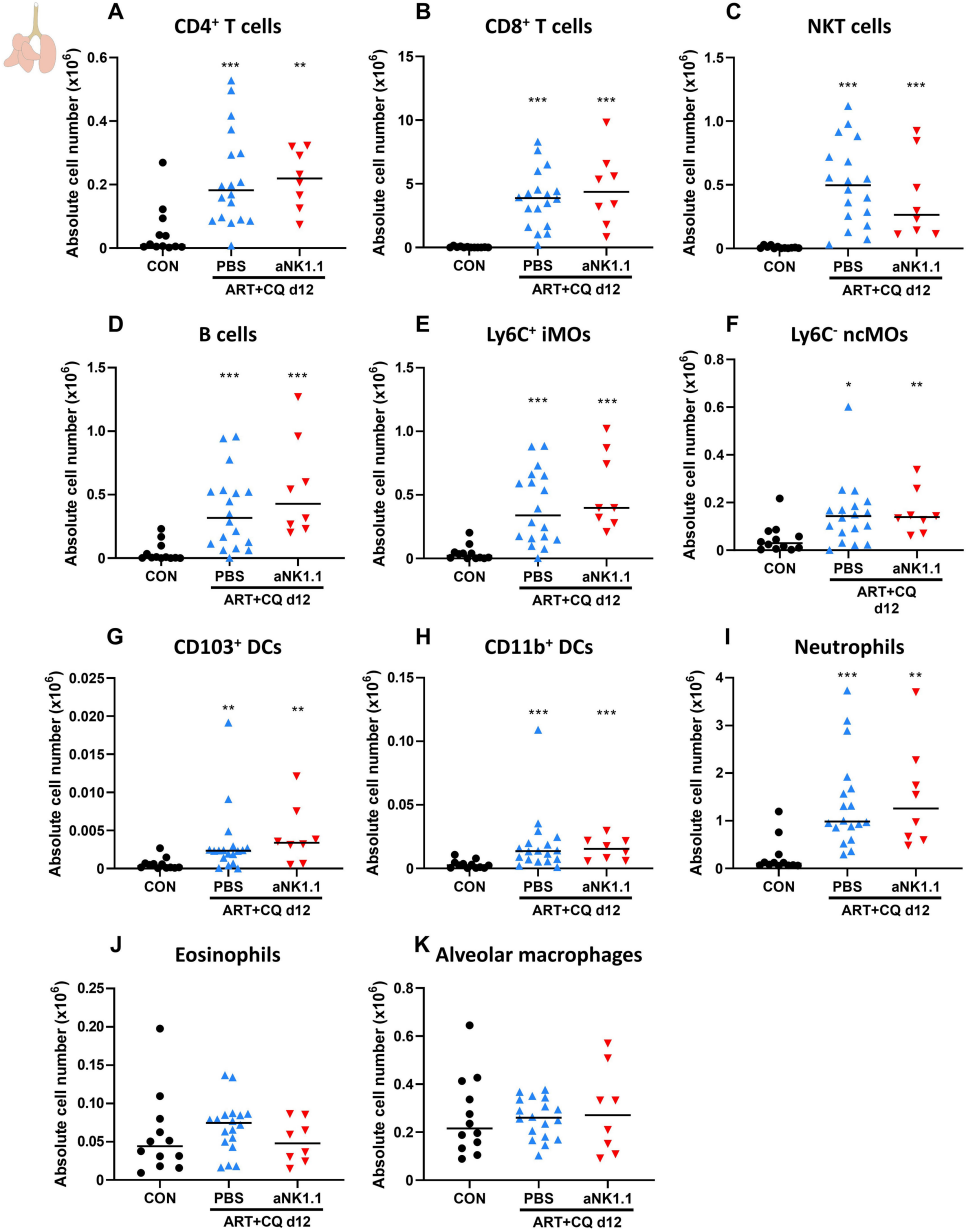
NK cell depletion has no effect on other lymphoid and myeloid cell populations present in the lungs of antimalarial-treated, *Pb*NK65-infected C57BL/6 mice. C57BL/6 mice were infected with *Pb*NK65. Daily treatment from 8 until 12 dpi with 10 mg/kg artesunate + 30 mg/kg chloroquine (ART+CQ). At 6 and 9 dpi, mice received 500 µg of anti-NK1.1 (PK136) depletion antibodies or PBS. Mice were dissected at 12 dpi. Pulmonary cells were isolated according to protocol 1 and flow cytometry was performed. The absolute number of **(A)** CD4^+^ T cells (CD45^+^ CD3^+^ NK1.1^-^ CD4^+^), **(B)** CD8^+^ T cells (CD45^+^ CD3^+^ NK1.1^-^ CD8^+^), **(C)** NKT cells (CD45^+^ CD3^+^ NK1.1^+^), **(D)** B cells (CD45^+^ CD3^-^ NK1.1^-^ B220^+^), **(E)** Ly6C^+^ inflammatory monocytes (iMOs; CD45^+^ Lin^-^ SiglecF^-^ Ly6G^-^ CD11b^hi^ MHCII^-^ Ly6C^+^), **(F)** Ly6C^-^ non-classical monocytes (ncMOs; CD45^+^ Lin^-^ SiglecF^-^ Ly6G^-^ CD11b^hi^ MHCII^-^ Ly6C^-^), **(G)** CD103^+^ dendritic cells (CD103^+^ DCs; CD45^+^ Lin^-^ SiglecF^-^ MHCII^+^ CD11c^+^ CD103^+^), **(H)** CD11b^+^ dendritic cells (CD11b^+^ DCs; CD45^+^ Lin^-^ SiglecF^-^ MHCII^+^ CD11c^+^ CD11b^+^ CD24^+^ CD64^-^), **(I)** Neutrophils (CD45^+^ Lin^-^ SiglecF^-^ CD11b^+^ Ly6G^+^), **(J)** Eosinophils (CD45^+^ SiglecF^+^ CD11b^+^ CD11c^-^) and **(K)** alveolar macrophages (CD45^+^ SiglecF^+^ CD11b^int^ CD11c^+^) in the lungs were calculated. For the myeloid cell gating, only Lineage-negative (Lin^-^) cells were selected based on CD3, CD19 and NK1.1. Data from three experiments. Each symbol represents data of an individual mouse. n = 12 for CON, n = 18 for ART+CQ PBS, n = 8 for ART+CQ aNK1.1. The non-parametric Mann-Whitney U test followed by the Holm-Bonferroni correction was used to determine significance between all groups. P-values were indicated as follows: *p<0.05, **p<0.01, ***p<0.001. Median in each group was indicated by a horizontal black line, unless indicated otherwise. Statistical differences compared to the uninfected control group are indicated with asterisk above the individual data sets and horizontal lines with asterisk on top indicate significant differences between groups.

Using flow cytometry, the number of other lymphoid and myeloid cell populations present in the lungs and spleen at 12 dpi was characterized. In the lungs, a similar increase in the number of CD4^+^ T cells (**Figure 2A**), CD8^+^ T cells (**Figure 2B**), NKT cells (**Figure 2C**) and B cells (**Figure 2D**) was observed at 12 dpi in the NK cell-depleted and non-depleted mice. In addition, the number of Ly6C^+^ inflammatory monocytes (iMOs; **Figure 2E**), Ly6C^-^ non-classical monocytes (ncMOs; **Figure 2F**), CD103^+^ dendritic cells (DCs) (**Figure 2G**), CD11b^+^ DCs (**Figure 2H**) and neutrophils (**Figure 2I**) was increased during resolution compared to non-infected controls, irrespectively of NK cell presence. No difference between any of the groups was observed in the number of pulmonary eosinophils (**Figure 2J**) and alveolar macrophages (**Figure 2K**). In the spleen at 12 dpi, a significant depletion of NK cells was observed due to *Pb*NK65 infection (**Supplementary Figures 3A, B**). Therefore, no further significant decrease in NK cells was obtained using anti-NK1.1 depletion antibodies. No differences between the NK cell-depleted and non-depleted mice were observed in the cell numbers of other lymphoid and myeloid populations (**Supplementary Figures 3C–L**).

### 3.3 Resolution of MA-ARDS upon antimalarial treatment is impaired in a transgenic NK cell-deficient mouse model

**Figure 3:**
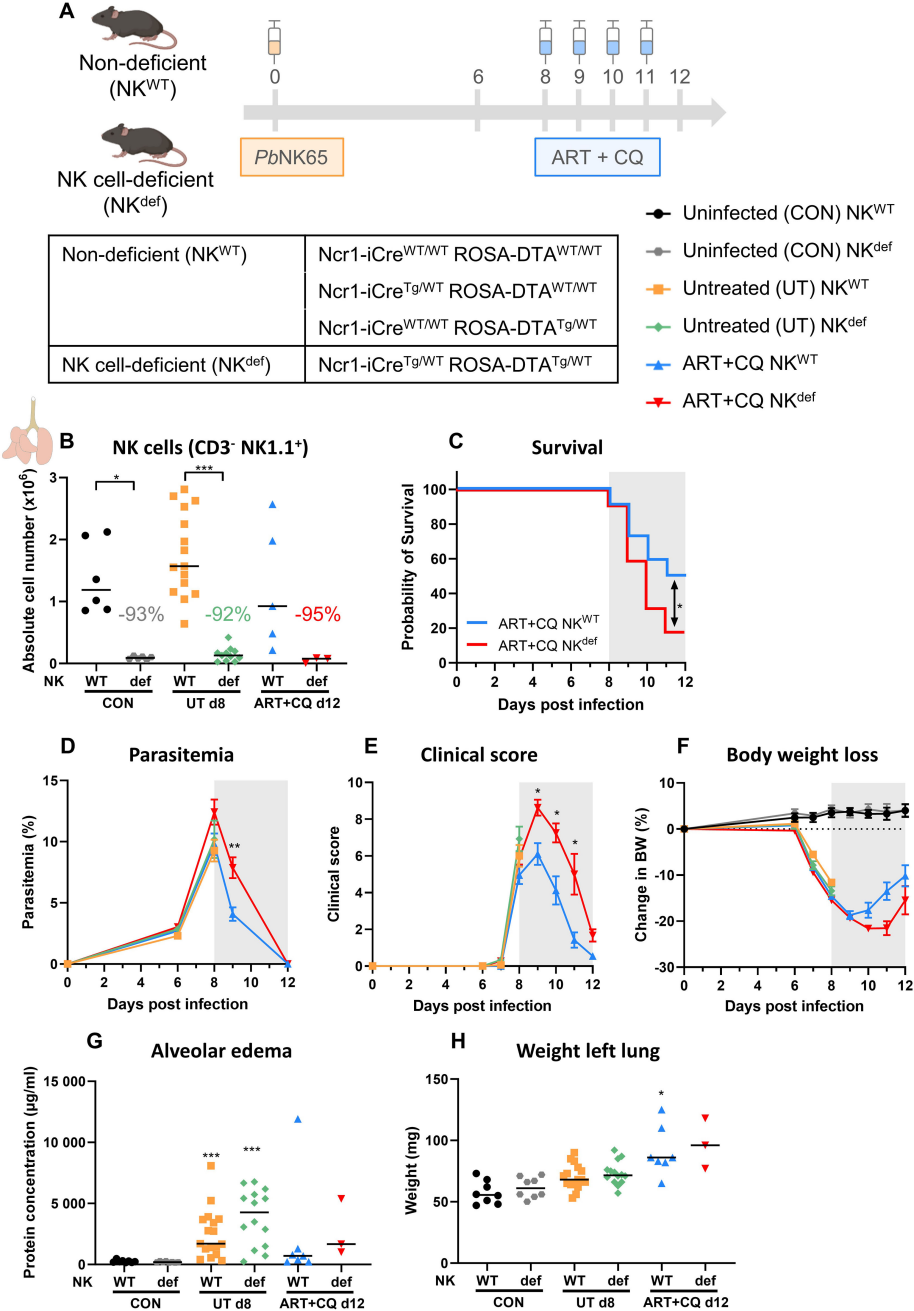
Recovery of MA-ARDS upon antimalarial treatment is also decreased in a NK cell-deficient mouse model. NK cell-deficient (Ncr1-iCre^Tg/WT^ ROSA-DTA^Tg/WT^) and non-deficient (Ncr1-iCre^Tg/WT^ ROSA-DTA^WT/WT^, Ncr1-iCre^WT/WT^ ROSA-DTA^Tg/WT^, Ncr1-iCre^WT/WT^ ROSA-DTA^WT/WT^) C57BL/6 mice were infected with *Pb*NK65. Daily treatment from 8 until 12 dpi with 10 mg/kg artesunate + 30 mg/kg chloroquine (ART+CQ). Mice were dissected at 8 dpi for untreated groups and 12 dpi for ART+CQ-treated groups. Pulmonary cells were isolated according to protocol 2 and flow cytometry was performed. **(A)** Schematic representation of the timing of infection and treatments in the mouse model. **(B)** The absolute number of NK cells (CD45^+^ CD3^-^ NK1.1^+^) present in the lungs was calculated. Data from three experiments. Each symbol represents data of an individual mouse. n = 6 for CON NK^WT^ and CON NK^def^, n = 15 for UT NK^WT^, n = 11 for UT NK^def^, n = 5 for ART+CQ NK^WT^, n = 3 for ART+CQ NK^def^. **(C)** Survival until 12 dpi. Data from four experiments. n = 22 for ART+CQ NK^WT^, n = 22 for ART+CQ NK^def^. **(D)** Parasitemia was determined daily starting at 6 dpi using Giemsa-stained blood smears. **(E)** Clinical score was monitored daily starting at 6 dpi. **(F)** Body weight loss was calculated compared to 0 dpi starting at 6 dpi. **(D-F)** Data from five experiments. Data are means ± SEM. n = 17 for UT NK^WT^, n = 14-15 for UT NK^def^, n = 11-21 for ART+CQ NK^WT^, n = 4-22 for ART+CQ NK^def^ with the highest number in each group indicating the number of mice at the start of the experiment and the lowest number in each group indicating the number of mice remaining at the end of each experiment due to death of the mice. **(G)** Level of alveolar edema was determined based on protein concentration in the BALF. **(H)** Unperfused left lung was weighed as another marker for lung pathology. **(G, H)** Data from four experiments. Each symbol represents data of an individual mouse. n = 8 for CON NK^WT^ and CON NK^def^, n = 16-17 for UT NK^WT^, n = 14 for UT NK^def^, n = 7 for ART+CQ NK^WT^, n = 3 for ART+CQ NK^def^. **(B-H)** The non-parametric Mann-Whitney U test followed by the Holm-Bonferroni correction was used to determine significance between each condition for the NK cell-deficient mice and between each condition for the non-deficient mice and between the NK cell-deficient and non-deficient mice within each condition. P-values were indicated as follows: *p<0.05, **p<0.01, ***p<0.001. Median in each group was indicated by a horizontal black line, unless indicated otherwise. Statistical differences compared to the appropriate uninfected control group are indicated with asterisk above the individual data sets and horizontal lines with asterisk on top indicate significant differences between groups.

To confirm the previously obtained results using the anti-NK1.1 depletion antibodies, Ncr1-iCre ROSA-DTA mice were used. Ncr1-iCre^Tg/WT^ ROSA-DTA^Tg/WT^ mice express the improved Cre recombinase in Ncr1-expressing cells, which are the NK cells (67). In addition, diphtheria toxin A (DTA) preceded by a floxed stop codon is inserted in the Rosa26 locus. Therefore, specifically in NK cells, the Cre recombinase excises the floxed stop codon resulting in expression of DTA and subsequent killing of the cell. These mice thus lack NK cells and are further referred to as NK cell-deficient (NK^def^) mice. In contrast, Ncr1-iCre^WT/WT^ ROSA-DTA^WT/WT^, Ncr1-iCre^Tg/WT^ ROSA-DTA^WT/WT^ and Ncr1-iCre^WT/WT^ ROSA-DTA^Tg/WT^ mice, have normal numbers of NK cells, and are further annotated as non-deficient (NK^WT^) mice. Both NK^def^ and NK^WT^ mice were infected with *Pb*NK65 and treated with antimalarial drugs at 8 dpi, when the first disease symptoms appear (**Figure 3A**). Untreated, *Pb*NK65-infected NK^def^ and NK^WT^ mice were dissected at 8 dpi, to investigate the effect of NK cells on the development of MA-ARDS, and ART+CQ-treated *Pb*NK65-infected NK^def^ and NK^WT^ mice were dissected at 12 dpi, to study the role of NK cells in disease resolution. The absence of NK cells (CD3^-^ NK1.1^+^) in the NK^def^ mice was confirmed in the lungs at each time-point, with an average of 92% NK cell decrease in each condition (uninfected, infected and treated) compared to NK^WT^ mice (**Figure 3B**).

In concordance with the anti-NK1.1 depletion experiments, survival after parasite killing was significantly decreased in the absence of NK cells (**Figure 3C**). However, in this NK depletion approach, a delayed decrease in parasitemia and clinical score was observed in the surviving NK^def^ mice (**Figures 3D, E**). Body weight loss was restored similarly in the NK^def^ and NK^WT^ mice, except for a non-significant trend (p = 0.0875) towards a lower body weight in NK^def^ mice versus NK^WT^ mice at 11 dpi (**Figure 3F**). Moreover, the level of alveolar edema and the weight of the left lung were similar in the mice that survived till 12 dpi (**Figures 3G, H**). Importantly, no significant difference in any of the parameters was observed at 8 dpi in the untreated NK^def^ and NK^WT^ mice, demonstrating the NK cells are not involved in the development of experimental MA-ARDS. Overall, these findings thus confirm that NK cells are crucial for the efficient resolution of experimental MA-ARDS upon antimalarial treatment.

### 3.4 NK cell deficiency only increased pulmonary NKT cells in uninfected controls, with no difference in other pulmonary and splenic lymphocyte populations

**Figure 4:**
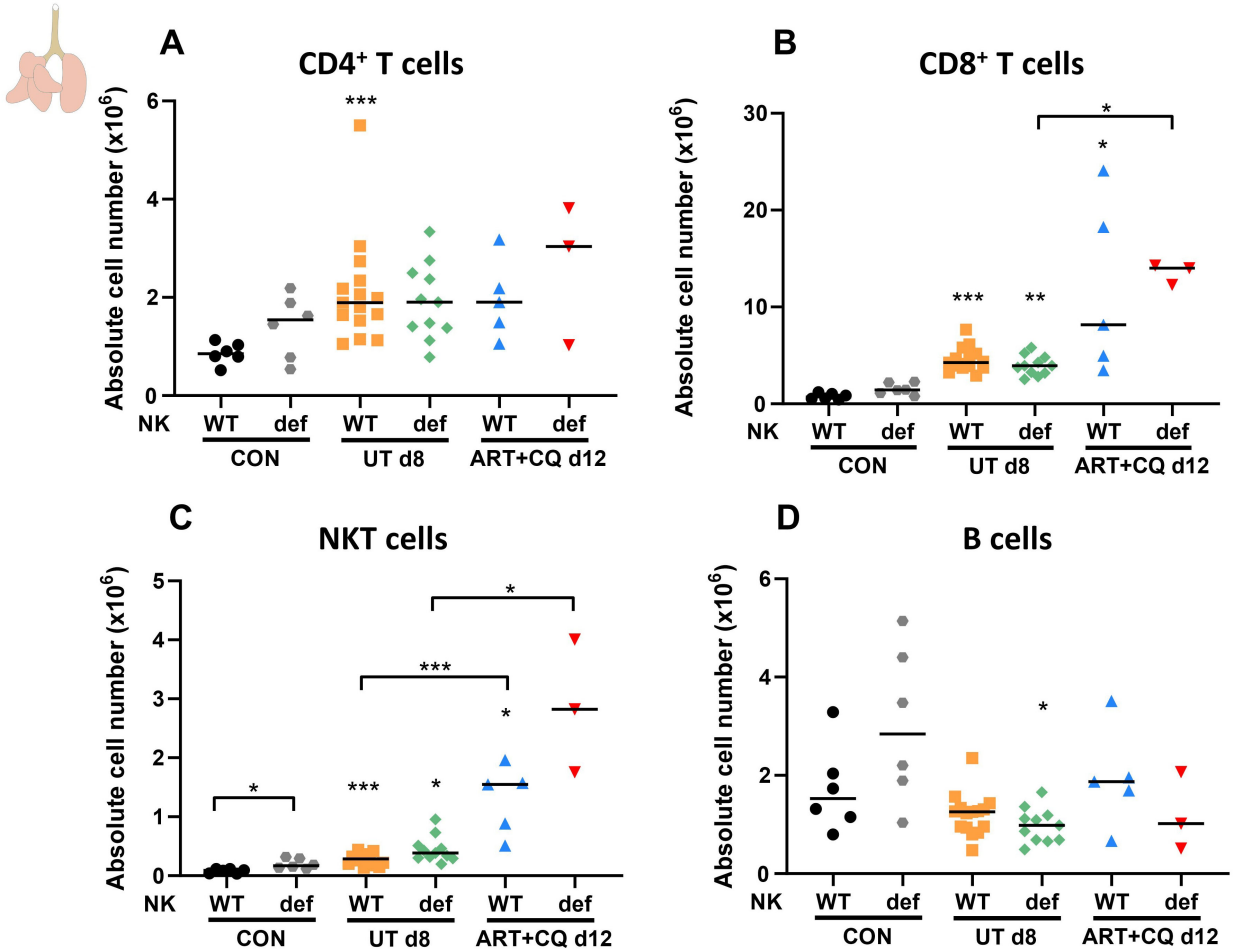
NK cell deficiency did not affect other lymphocyte numbers, except for an increase in NKT cells in uninfected mice. NK cell-deficient (Ncr1-iCre^Tg/WT^ ROSA-DTA^Tg/WT^) and non-deficient (Ncr1-iCre^Tg/WT^ ROSA-DTA^WT/WT^, Ncr1-iCre^WT/WT^ ROSA-DTA^Tg/WT^, Ncr1-iCre^WT/WT^ ROSA-DTA^WT/WT^) C57BL/6 mice were infected with *Pb*NK65. Daily treatment from 8 until 12 dpi with 10 mg/kg artesunate + 30 mg/kg chloroquine (ART+CQ). Mice were dissected at 8 dpi for untreated groups and 12 dpi for ART+CQ-treated groups. Pulmonary cells were isolated according to protocol 2 and flow cytometry was performed. The absolute number of **(A)** CD4^+^ T cells (CD45^+^ CD3^+^ NK1.1^-^ CD4^+^), **(B)** CD8^+^ T cells (CD45^+^ CD3^+^ NK1.1^-^ CD8^+^), **(C)** NKT cells (CD45^+^ CD3^+^ NK1.1^+^) and **(D)** B cells (CD45^+^ CD3^-^ NK1.1^-^ B220^+^) in the lungs were calculated. Data from three experiments. Each symbol represents data of an individual mouse. n = 6 for CON NK^WT^ and CON NK^def^, n = 15 for UT NK^WT^, n = 11 for UT NK^def^, n = 5 for ART+CQ NK^WT^, n = 3 for ART+CQ NK^def^. The non-parametric Mann-Whitney U test followed by the Holm-Bonferroni correction was used to determine significance between each condition for the NK cell-deficient mice and between each condition for the non-deficient mice and between the NK cell-deficient and non-deficient mice within each condition. P-values were indicated as follows: *p<0.05, **p<0.01, ***p<0.001. Median in each group was indicated by a horizontal black line, unless indicated otherwise. Statistical differences compared to the appropriate uninfected control group are indicated with asterisk above the individual data sets and horizontal lines with asterisk on top indicate significant differences between groups.

Furthermore, the number of pulmonary and splenic lymphocyte populations was determined using flow cytometry. As described previously, CD8^+^ T cells in the lungs were considerably increased upon infection, and a mild increase in CD4^+^ T cells was also noticed (66). However, the number of CD4^+^ and CD8^+^ T cells was not different between the NK^def^ and NK^WT^ mice at all time-points (**Figures 4A, B**).

In contrast, an increase in pulmonary NKT cells was observed in the absence of NK cells, although this was only significant in the uninfected control mice (**Figure 4C**). The number of B cells present in the lungs was not changed in the absence of NK cells (**Figure 4D**). The decreased number of NK cells in NK^def^ mice was also confirmed in the spleen for uninfected and infected mice at 8 dpi, while no differences between the NK^def^ and NK^WT^ mice were observed at any time-point for other splenic lymphocyte populations (**Supplementary Figure 4**).

### 3.5 Lung pathology was not affected by the absence of NK cells in the early phase of resolution

**Figure 5:**
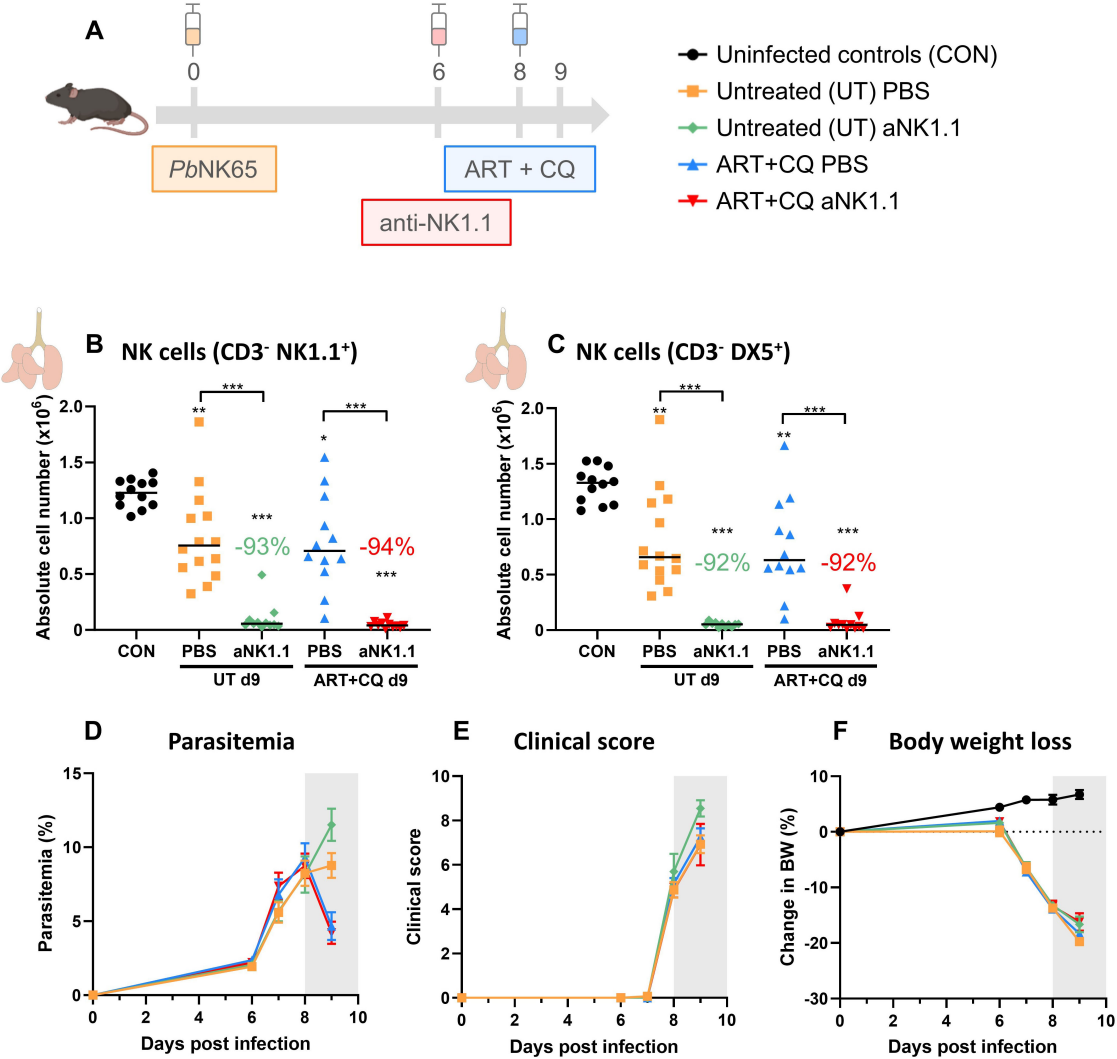
NK cell depletion has no effect on MA-ARDS at 9 dpi. C57BL/6 mice were infected with *Pb*NK65. Treatment at 8 dpi with 10 mg/kg artesunate + 30 mg/kg chloroquine (ART+CQ). At 6 dpi, mice were injected i.p. with 500 µg of anti-NK1.1 (PK136) depletion antibodies or PBS. Mice were dissected at 9 dpi. Pulmonary cells were isolated according to protocol 2 and flow cytometry was performed. **(A)** Schematic representation of the timing of infection and treatments in the mouse model. **(B, C)** Number of NK cells present in the lungs gated as CD45^+^ CD3^-^ NK1.1^+^ **(B)** or as CD45^+^ CD3^-^ DX5^+^ **(C)**. Data from three experiments. Each symbol represents data of an individual mouse. n = 12 for CON, n = 14 for UT PBS, n = 11 for UT anti-NK1.1 (aNK1.1), n = 12 for ART+CQ PBS and n = 11 for ART+CQ aNK1.1 group. **(D)** Parasitemia was determined daily starting at 6 dpi using Giemsa-stained blood smears. **(E)** Clinical score was monitored daily starting at 6 dpi. **(F)** Body weight loss was calculated compared to 0 dpi starting at 6 dpi. **(D-F)** Data from five experiments. Data are shown as means ± SEM. n = 14-15 for UT PBS, n = 11-13, n = 12-15 for ART+CQ PBS, n = 11-14 for ART+CQ aNK1.1 with the highest number in each group indicating the number of mice at the start of the experiment and the lowest number in each group indicating the number of mice remaining at the end of each experiment due to death of the mice. **(B-F)** The non-parametric Mann-Whitney U test followed by the Holm-Bonferroni correction was used to determine significance between all groups, except for the comparison untreated (UT) PBS with antimalarial drug-treated (ART+CQ) anti-NK1.1 and the comparison UT anti-NK1.1 with ART+CQ PBS. P-values were indicated as follows: *p<0.05, **p<0.01, ***p<0.001. Median in each group was indicated by a horizontal black line, unless indicated otherwise. Statistical differences compared to the uninfected control group are indicated with asterisk above the individual data sets and horizontal lines with asterisk on top indicate significant differences between groups.

By using both NK cell depleting approaches, we observed the majority of deaths (50-75% of deaths) due to the absence of NK cells in the early phase of disease resolution (at 9-10 dpi). Therefore, the effect of NK cell depletion using the anti-NK1.1 antibodies was studied at 9 dpi in both untreated, *Pb*NK65-infected C57BL/6 mice and *Pb*NK65-infected C57BL/6 mice that received one day of antimalarial treatment (**Figure 5A**). Both in the untreated and the ART+CQ-treated groups, a significant decrease in pulmonary NK cells was observed in the NK cell-depleted mice (both when gating on CD3^-^ NK1.1^+^ and on CD3^-^ DX5^+^) (**Figures 5B, C**). Consistent with the experiment shown in **Figure 1**, the absence of NK cells had no impact on the parasitemia, clinical score and body weight (**Figures 5D–F**).

**Figure 6:**
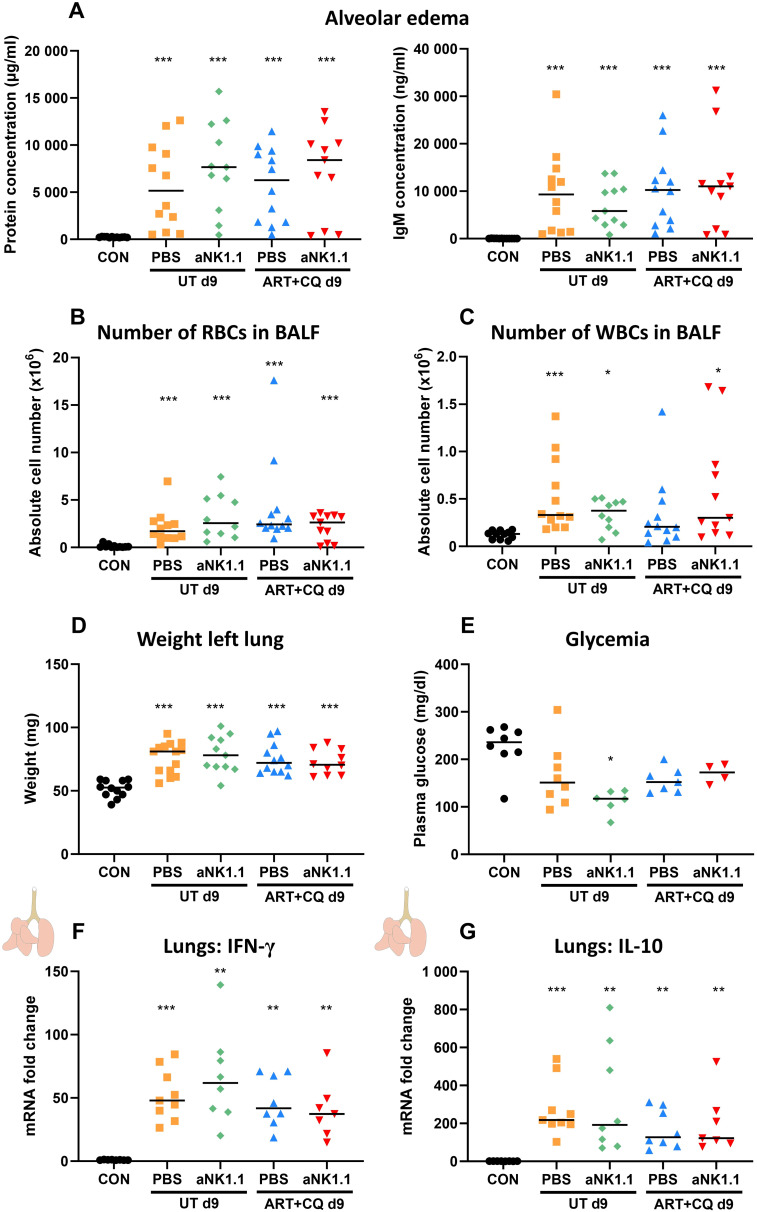
Lung pathology was not affected by the absence of NK cells at 9 dpi. C57BL/6 mice were infected with *Pb*NK65. Treatment at 8 dpi with 10 mg/kg artesunate + 30 mg/kg chloroquine (ART+CQ). At 6 dpi, mice were injected i.p. with 500 µg of anti-NK1.1 (PK136) depletion antibodies or PBS. Mice were dissected at 9 dpi. **(A)** Level of alveolar edema was determined based on protein concentration (**A**, left) and IgM concentration (**A**, right) in the BALF. **(B, C)** The number of RBCs **(B)** and WBCs **(C)** present in the BALF was counted using a Bürker chamber. **(D)** Unperfused left lungs were weighed as another marker for lung pathology. **(E)** Glucose levels in plasma were determined using OneTouch Verio glucometer. **(A-E)** Data from two **(E)** or three **(A-D)** experiments. Each symbol represents data of an individual mouse. n = 8-12 for CON, n = 8-14 for UT PBS, n = 6-11 for UT aNK1.1, n = 7-12 for ART+CQ PBS, n = 4-11 for ART+CQ aNK1.1. **(F, G)** The difference in the mRNA fold expression in left lungs homogenates at 9 dpi was determined compared to uninfected controls for **(F)** IFN-γ and **(G)** IL-10. Data from two experiments. Each symbol represents data of an individual mouse. n = 8 for CON, n = 9 for UT PBS, n = 8 for UT aNK1.1, n = 8 for ART+CQ PBS, n = 7 for ART+CQ aNK1.1. **(A-G)** The non-parametric Mann-Whitney U test followed by the Holm-Bonferroni correction was used to determine significance between all groups, except for the comparison untreated (UT) PBS with antimalarial drug-treated (ART+CQ) anti-NK1.1 and the comparison UT anti-NK1.1 with ART+CQ PBS. P-values were indicated as follows: *p<0.05, **p<0.01, ***p<0.001. Median in each group was indicated by a horizontal black line, unless indicated otherwise. Statistical differences compared to the uninfected control group are indicated with asterisk above the individual data sets and horizontal lines with asterisk on top indicate significant differences between groups.

In addition, the level of alveolar edema, based on either the total alveolar protein concentration and IgM concentration, was not affected by NK cell depletion or by one day of antimalarial treatment (**Figure 6A**). Also, no significant differences in alveolar RBC and leukocyte numbers were found between the NK cell-depleted and non-depleted mice and between the untreated and ART+CQ-treated mice (**Figures 6B, C**). Weights of the left lung, as another indicator for lung pathology, were similar in all infected groups (**Figure 6D**). In patients, hypoglycemia is an important feature of severe malaria, contributing to a poor prognosis, especially in children and pregnant women (71). Therefore, plasma glucose levels were determined as hypoglycemia may be the cause of the observed lethality in the NK cell-depleted group. However, glycemia was not different between any of the groups, except for a decrease in the untreated, NK cell-depleted group (**Figure 6E**). Next, the inflammatory status in the left lung was determined by measuring the IFN-γ and IL-10 mRNA expression, a pro- and anti-inflammatory marker respectively. Both IFN-γ and IL-10 expression was highly increased in the lungs of all infected groups compared to uninfected controls at 9 dpi, but no difference between NK cell-depleted and non-depleted mice was observed (**Figures 6F, G**). Also, expression of IFN-γ and IL-10 was similar between UT PBS and ART+CQ PBS suggesting that one day of antimalarial treatment had no significant effect on the pulmonary inflammation status at 9 dpi. In conclusion, neither the absence of NK cells, nor treatment with antimalarial drugs for one day affected lung pathology upon *Pb*NK65 infection at 9 dpi.

### 3.6 NK cell depletion did not have an effect on pulmonary and splenic leukocyte numbers in infected mice at the early phase of MA-ARDS resolution

**Figure 7:**
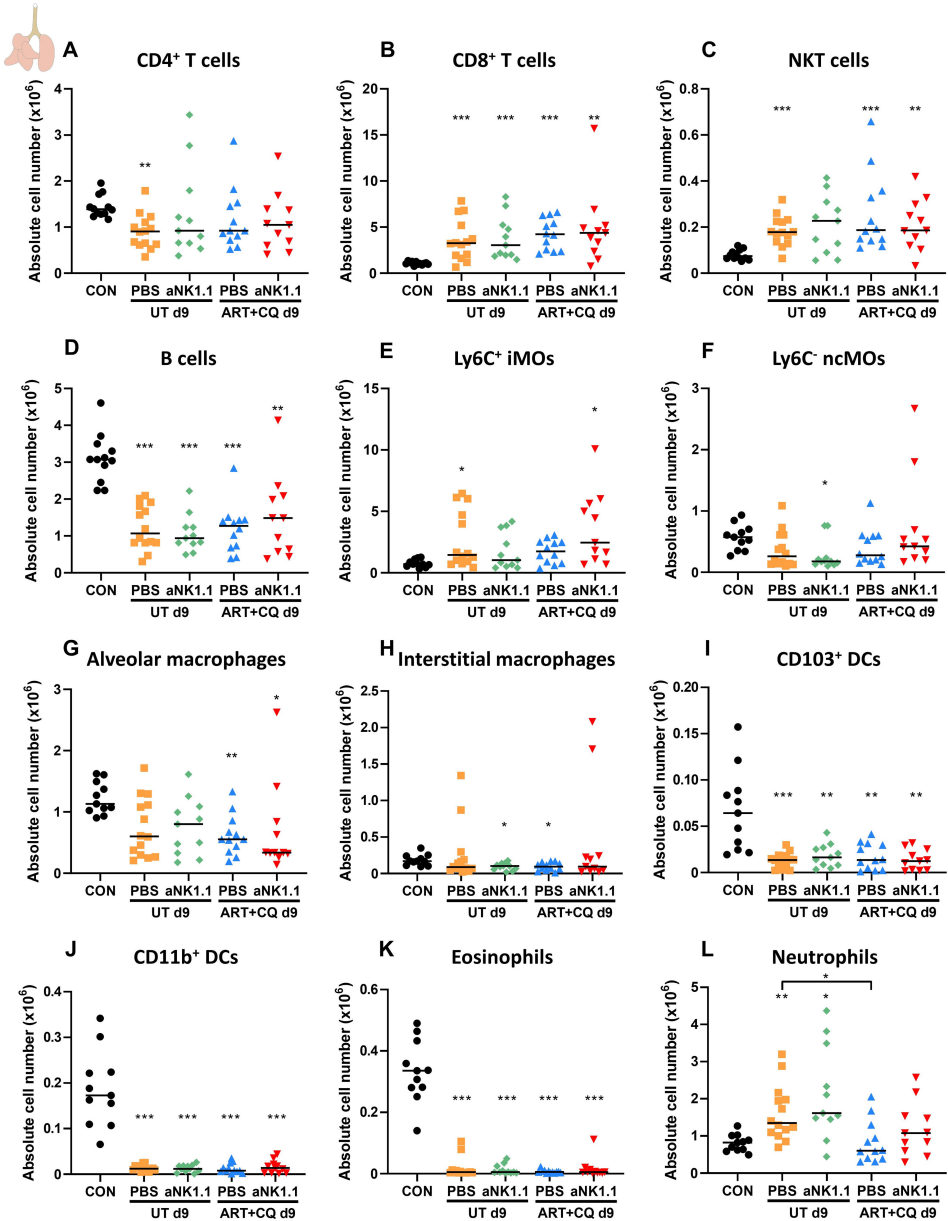
NK cell depletion has no effect on other lymphoid and myeloid cell populations present in the lungs at 9 dpi. C57BL/6 mice were infected with *Pb*NK65. Treatment at 8 dpi with 10 mg/kg artesunate + 30 mg/kg chloroquine (ART+CQ). At 6 dpi, mice were injected i.p. with 500 µg of anti-NK1.1 (PK136) depletion antibodies or PBS. Mice were dissected at 9 dpi. Pulmonary cells were isolated according to protocol 2 and flow cytometry was performed. The absolute number of **(A)** CD4^+^ T cells (CD45^+^ CD3^+^ NK1.1^-^ CD4^+^), **(B)** CD8^+^ T cells (CD45^+^ CD3^+^ NK1.1^-^ CD8^+^), **(C)** NKT cells (CD45^+^ CD3^+^ NK1.1^+^), **(D)** B cells (CD45^+^ CD3^-^ NK1.1^-^ B220^+^), **(E)** Ly6C^+^ inflammatory monocytes (iMOs; CD45^+^ Lin^-^ SiglecF^-^ Ly6G^-^ CD11b^hi^ MHCII^-^ Ly6C^+^), **(F)** Ly6C^-^ non-classical monocytes (ncMOs; CD45^+^ Lin^-^ SiglecF^-^ Ly6G^-^ CD11b^hi^ MHCII^-^ Ly6C^-^), **(G)** alveolar macrophages (CD45^+^ SiglecF^+^ CD11b^int^ CD11c^+^), **(H)** interstitial macrophages (CD45^+^ Lin^-^ SiglecF^-^ Ly6G^-^ CD11b^hi^ MHCII^+^ CD24^-^ CD64^+^), **(I)** CD103^+^ dendritic cells (CD103^+^ DCs; CD45^+^ Lin^-^ SiglecF^-^ MHCII^+^ CD11c^+^ CD103^+^), **(J)** CD11b^+^ dendritic cells (CD11b^+^ DCs; CD45^+^ Lin^-^ SiglecF^-^ MHCII^+^ CD11c^+^ CD11b^+^ CD24^+^ CD64^-^), **(K)** Eosinophils (CD45^+^ SiglecF^+^ CD11b^+^ CD11c^-^) and **(L)** Neutrophils (CD45^+^ Lin^-^ SiglecF^-^ CD11b^+^ Ly6G^+^) in the lungs were calculated. For the myeloid cell gating, only Lineage-negative (Lin^-^) cells were selected based on CD3, CD19 and NK1.1. Data from three experiments. Each symbol represents data of an individual mouse. n = 11-12 for CON, n = 14 for UT PBS, n = 11 for UT aNK1.1, n = 12 for ART+CQ PBS, n = 11 for ART+CQ aNK1.1. The non-parametric Mann-Whitney U test followed by the Holm-Bonferroni correction was used to determine significance between all groups, except for the comparison untreated (UT) PBS with antimalarial drug-treated (ART+CQ) anti-NK1.1 and the comparison UT anti-NK1.1 with ART+CQ PBS. P-values were indicated as follows: *p<0.05, **p<0.01, ***p<0.001. Median in each group was indicated by a horizontal black line, unless indicated otherwise. Statistical differences compared to the uninfected control group are indicated with asterisk above the individual data sets and horizontal lines with asterisk on top indicate significant differences between groups.

Since most NK cell-depleted mice died in the beginning of the resolution phase (at 9-10 dpi), pulmonary leukocytes were studied at 9 dpi. In lungs of infected mice at 9 dpi, a mild decrease in the number of CD4^+^ T cells was observed, which was only significant for the untreated PBS group (**Figure 7A**). In contrast, the number of CD8^+^ T cells (**Figure 7B**) and NKT cells (**Figure 7C**) increased compared to non-infected controls. The number of B cells significantly decreased upon infection (**Figure 7D**). One day of antimalarial treatment and NK cell depletion had no effect on the number of CD4^+^ T cells, CD8^+^ T cells, NKT and B cells (**Figures 7A–D**).

Increased numbers of Ly6C^+^ iMOs (**Figure 7E**, significant for the untreated PBS and the ART+CQ anti-NK1.1 groups) and a decreased number of Ly6C^-^ ncMOs (**Figure 7F**, only significant for the untreated anti-NK1.1 group), alveolar and interstitial macrophages (**Figures 7G, H**) was observed at 9 dpi in infected mice compared to uninfected controls. CD103^+^ DCs (**Figure 7I**), CD11b^+^ DCs (**Figure 7J**) and eosinophils (**Figure 7K**) decreased in the lungs of infected mice at 9 dpi. The number of neutrophils (**Figure 7L**) was increased in untreated *Pb*NK65-infected C57BL/6 mice compared to both uninfected controls, while this was not the case for the ART+CQ-treated *Pb*NK65-infected C57BL/6 mice. At 9 dpi similar as to 12 dpi, NK cell depletion had no effect on the myeloid cell populations present in lungs of infected mice (**Figures 7E–L**). In the spleen, a decreasing trend was observed for all lymphoid and myeloid populations in infected mice at 9 dpi with no difference between NK cell-depleted and non-depleted mice (**Supplementary Figure 5**).

### 3.7 NK cells drastically upregulate their expression of *Il10* in response to *Pb*NK65 infection

**Figure 8:**
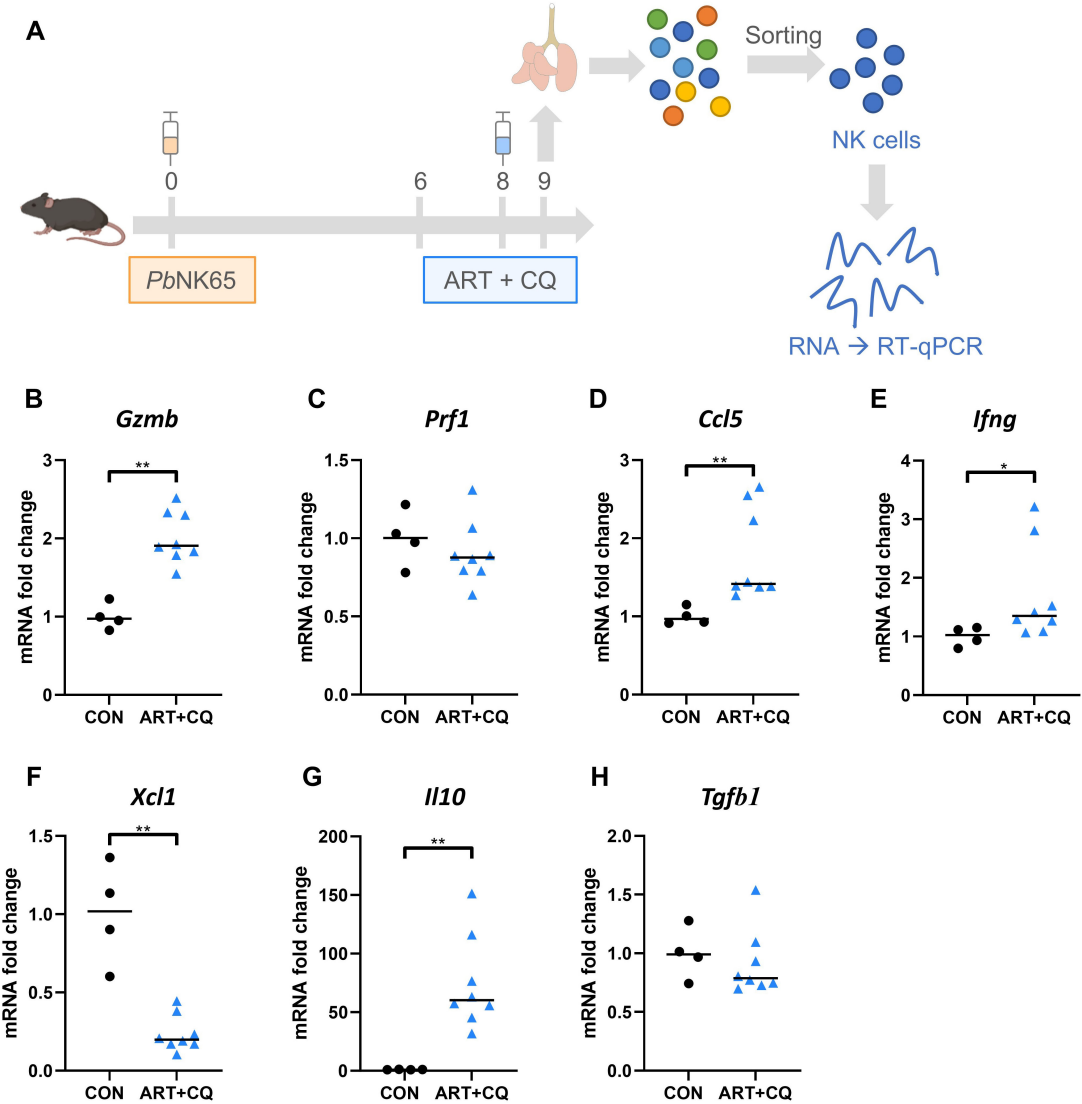
NK cells drastically upregulate their expression of IL-10 in response to *Pb*NK65 infection. C57BL/6 mice were infected with *Pb*NK65. Mice were treated at 8 dpi with 10 mg/kg artesunate + 30 mg/kg chloroquine (ART+CQ). Mice were dissected at 9 dpi. Pulmonary cells were isolated according to protocol 2 and fluorescence activated cell sorting was performed to isolate NK cells (CD3^-^ NK1.1^+^ DX5^+^). **(A)** Schematic representation of experiment set-up. The difference in the mRNA fold expression in the NK cells at 9 dpi was determined compared to uninfected controls for **(B)** Granzyme B (*Gzmb*), **(C)** Perforin (*Prf1*), **(D)** *Ccl5*, **(E)** *Ifng*, **(F)** *Xcl1*, **(G)** *Il10* and **(H)** *Tgfb1*. Data from two experiments. Each symbol represents data of an individual mouse. n = 4 for CON, n = 8 for ART+CQ. The non-parametric Mann-Whitney U test followed by the Holm-Bonferroni correction was used to determine significance between all groups. P-values were indicated as follows: *p<0.05, **p<0.01. Median in each group was indicated by a horizontal black line, unless indicated otherwise. Statistical differences compared to the uninfected control group are indicated with asterisk above the individual data sets and horizontal lines with asterisk on top indicate significant differences between groups.

Because our experiments show that NK cells are contributing to the initiation of resolution of MA-ARDS, we explored the potential mechanism. To this end, NK cells (CD3^-^ NK1.1^+^ DX5^+^, **Supplementary Figure 2**) from lungs of *Pb*NK65-infected C57BL/6 mice, that received antimalarial treatment at 8 dpi, and uninfected control mice were sorted at 9 dpi and RT-qPCR was performed for cytotoxic molecules, cytokines and chemokines, as schematically presented in **Figure 8A**.

At 9 dpi, NK cells showed an increase in Granzyme B (*Gzmb*), but not Perforin (*Prf1*) expression, two essential effector molecules for cytotoxicity compared to the uninfected control group (**Figures 8B, C**), which might suggest an increased cytotoxicity of NK cells during the initiation of recovery. Despite some variability, NK cell-specific expression of *Ccl5* and *Ifng* was also significantly increased in infected mice, indicating presence of pulmonary inflammation at the early phase of resolution (**Figures 8D, E**). *Xcl1*, also called lymphotactin, is a chemokine secreted by NK cells and CD8^+^ T cells and attract antigen-presenting cells, such as dendritic cells (72). Expression of *Xcl1* by NK cells was decreased in the ART+CQ-treated, *Pb*NK65-infected C57BL/6 mice compared to uninfected mice at 9 dpi (**Figure 8F**). During inflammation, NK cells have also been described to have a regulatory function, potentially via the production of anti-inflammatory cytokines, such as IL-10 and TGF-β (5, 6). In this model, NK cells drastically upregulate the expression of *Il10*, but not *Tgfb1* (**Figures 8G, H**).

### 3.8 Inhibition of the IL-10 receptor has no effect on the resolution of MA-ARDS

**Figure 9:**
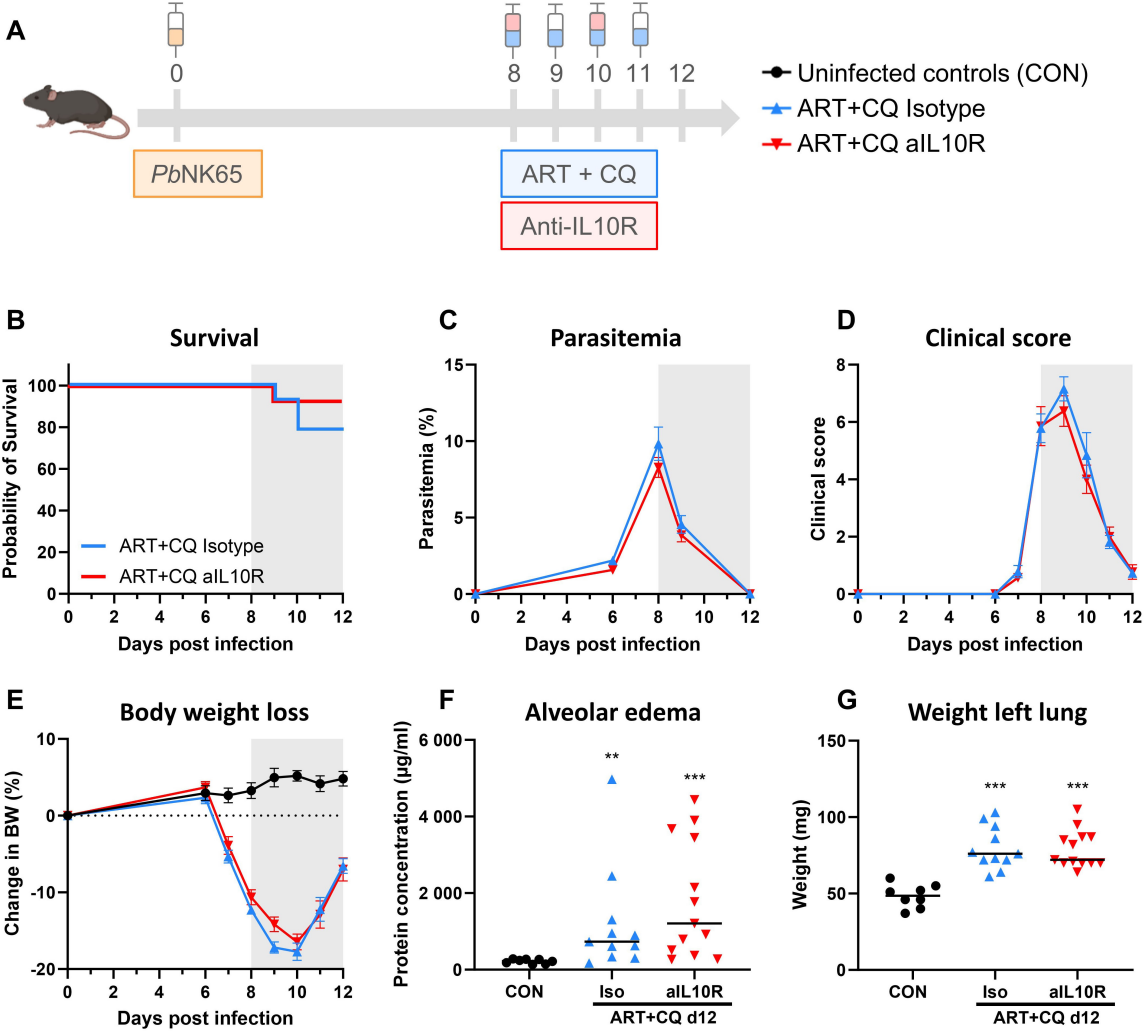
Inhibition of the IL-10/IL-10R axis had no effect on the resolution of experimental MA-ARDS. C57BL/6 mice were infected with *Pb*NK65. Daily treatment from 8 until 12 dpi with 10 mg/kg artesunate + 30 mg/kg chloroquine (ART+CQ). At 8 and 10 dpi, mice received 300 µg of anti-IL10R (1B1.3A) antibodies or isotype control antibodies. Mice were dissected at 12 dpi. **(A)** Schematic representation of the timing of infection and treatments in the mouse model. **(B)** Survival until 12 dpi. Data from two experiments. n = 14 for ART+CQ Isotype and ART+CQ aIL10R. **(C)** Parasitemia was determined daily starting at 6 dpi using Giemsa-stained blood smears. **(D)** Clinical score was monitored daily starting at 6 dpi. **(E)** Body weight loss was calculated compared to 0 dpi starting at 6 dpi. **(B-E)** Data from two experiments. Data are represented as means ± SEM. n = 8 for CON, n = 14 for ART+CQ Isotype and ART+CQ aIL10R. **(F)** Level of alveolar edema was determined based on protein concentration in the BALF. **(G)** Unperfused left lung was weighed as another marker for lung edema. **(F-G)** Data from two experiments. Each symbol represents data of an individual mouse. n = 8 for CON, n = 11 for ART+CQ Isotype, n = 13 for ART+CQ aIL10R. **(B-G)** The non-parametric Mann-Whitney U test followed by the Holm-Bonferroni correction was used to determine significance between all groups. P-values were indicated as follows: **p<0.01, ***p<0.001. Median in each group was indicated by a horizontal black line, unless indicated otherwise. Statistical differences compared to the uninfected control group are indicated with asterisk above the individual data sets and horizontal lines with asterisk on top indicate significant differences between groups.

The role of the IL-10/IL-10 receptor (IL-10R) axis was investigated in the resolution of MA-ARDS. Therefore, ART+CQ-treated, *Pb*NK65-infected C57BL/6 mice were injected with anti-IL10R antibodies on 8 and 10 dpi (**Figure 9A**). Inhibition of the IL-10/IL-10R axis had no effect on the resolution of MA-ARDS, since no significant differences in survival (**Figure 9B**), parasitemia (**Figure 9C**), clinical score (**Figure 9D**), body weight loss (**Figure 9E**), level of alveolar edema (**Figure 9F**) and weight of the left lung (**Figure 9G**) were found between the anti-IL10R-treated and isotype-treated groups.

In conclusion, NK cells induce the resolution of MA-ARDS leading to increased survival. This is paralleled by increased expression of the cytotoxicity marker *Gzmb* and the anti-inflammatory cytokine *Il10*. However, inhibition of the global IL-10/IL-10R axis had no effect on the resolution of MA-ARDS, suggesting that there is probably a combination of factors at play.

## 4 Discussion

NK cells were found to demonstrate a protective role in the resolution of experimental MA-ARDS, since survival was decreased both upon depletion with anti-NK1.1 antibodies and when using NK cell-deficient mice. This was paralleled by high *Il10* expression by the NK cells, suggesting a potent regulatory phenotype. In contrast, NK cells were not essential in the development of experimental MA-ARDS, as shown previously (44). Inhibition of the IL-10/IL-10R axis had no effect on the resolution of MA-ARDS, suggesting that the pro-resolving effect of NK cells cannot be solely attributed to the production of IL-10. Moreover, IL-10R inhibition did not affect the clearance of parasites upon antimalarial treatment.

As published previously, we have established a robust resolution model for experimental MA-ARDS wherein more than 80% of *Pb*NK65-infected mice survive the pulmonary complication using the combination of artesunate and chloroquine (66). This was again confirmed in the non-depleted C57BL/6 mice in the experiments using anti-NK1.1 antibodies. In the NK^WT^ mice, a lower survival (± 50%) was observed compared to standard non-transgenic C57BL/6 mice (> 80%). We cannot fully exclude that minor differences in the genetic background are responsible for this. As shown in the SNP analysis of these mice (**Supplementary Table 1**), 99.2-99.6% of the genome corresponds to C57BL/6 mice, which corresponds to 7-8 back-crossings. Due to the high variability in susceptibility of different mouse strains to *Plasmodium* parasites, the small fraction of non-C57BL/6 DNA might possibly cause the increased susceptibility of these mice to *Pb*NK65 infection. Nevertheless, survival upon antimalarial treatment was significantly decreased in anti-NK1.1 injected mice compared to PBS injected mice and in NK^def^ mice compared to NK^WT^ mice. In contrast, no effect of NK cell depletion was noted on the development of MA-ARDS [in accordance to our previous publication (44)]. Similar to the data by Yan et al., also no effect of NK cell depletion was seen in healthy non-infected mice (73). While NK cell depletion could possibly increase susceptibility to other pathogens, our mice were housed in a high-level SPF facility in individually ventilated cages, excluding contamination or interference by unwanted pathogens. This confirms that NK cells effectively contribute to the disease resolution in experimental MA-ARDS.

Contradicting, such as both pro- and anti-inflammatory, roles of NK cells in malaria have been described in literature. Multiple studies using either anti-asialo GM1 antibodies or the more specific anti-NK1.1 depletion antibodies, could not find a role for NK cells in anti-parasitic immunity (27, 29, 37–40) and in malaria-associated pathology (41–44). These data are in line with our results showing that the development of experimental MA-ARDS is not affected in the absence of NK cells, demonstrated by anti-NK1.1 depleting antibodies (44) and by Ncr1-iCre^Tg/WT^ ROSA-DTA^Tg/WT^ mice. However, we demonstrated with both models that NK cells are crucial in the resolution of experimental MA-ARDS during antimalarial treatment and that these NK cells may have a regulatory phenotype. In *P. falciparum*-infected patients, adaptive NK cells contributed to the killing of infected RBCs through ADCC (23). Such adaptive NK cells also participated in anti-malarial immunity by ADCC against merozoites and correlated with protection from symptomatic malaria (24, 25).

NK cells also have an immunoregulatory role via their NK cell-mediated cytotoxicity (NKCC) against pro-inflammatory cells (11–15, 74, 75). In severe asthma patients, NK cell-mediated resolution was found to be disabled, resulting in aberrant killing of pro-inflammatory cells and thus impaired resolution (76, 77). In a mouse model for autoinflammatory arthritis, it was found that NK cells target and kill activated monocytes and dendritic cells, via a NKG2D-dependent mechanism (16). In contrast, in a mouse model of excisional skin wounding, depletion of NK cells resulted in increased wound healing, suggesting that NK cells inhibit wound healing (78, 79). In our study, expression of *Gzmb*, but not *Prf1*, was increased in NK cells at 9 dpi compared to NK cells from uninfected control mice. This suggests that NKCC might be increased, inducing apoptosis of pro-inflammatory cells and thereby promoting resolution. However, since no effect of NK cell deficiency on other splenic and pulmonary leukocyte populations was found, this seems unlikely.

As reviewed by Martinez-Espinosa et al., IL-10 production by NK cells might be both detrimental and beneficial during infection (6). NK cell-derived IL-10 production can be detrimental by inhibiting protective immune responses against pathogens and might therefore promote infection persistence and aggravation. For example, in mouse models for *Listeria monocytogenes, Streptococcus pneumoniae* and visceral leishmaniasis, NK cell-derived IL-10 was found to increase susceptibility to infection (80–82). A similar role for IL-10 producing NK cells was described in patients with chronic viral infections, such as hepatitis B and C (6). In contrast, IL-10 production by NK cells may also limit immunopathology. In murine sepsis and upon infection of immunocompromised mice with murine cytomegalovirus, NK cell-derived IL-10 was found to prevent an exacerbated immune response (8, 9).

The role of IL-10 in malaria has been studied elaborately, as reviewed in detail by Kumar et al. (83). IL-10 protects against severe malarial immunopathology by limiting the excessive immune response, as IL-10 was shown to prevent CM and liver pathology (58–60, 84–87). In these studies, the IL-10/IL-10R axis was blocked using antibodies or knock-out mice or stimulated by the administration of recombinant IL-10. This resulted in decreased or increased survival, respectively. Moreover, IL-15C-induced production of IL-10 by NK cells prevents development of experimental CM (7). Parasitemia upon infection with *Pb*ANKA and *P. chabaudi* AS was found to be unaffected by IL-10 (59, 86). In contrast, Claser et al. observed a decrease in parasitemia, but an increased parasite accumulation in the brain upon administration of anti-IL-10R blocking antibodies in *Pb*ANKA-infected BALB/c mice (58). On the contrary, upon infection with *Pb*NK65-NY and *P. yoelii*, a decrease in parasitemia in the absence of IL-10 was found, suggesting that IL-10 impairs the protective anti-parasitic immune response (58, 60, 61). Omer et al. found that both IL-10 and TGF-β need to be inhibited in order to obtain an adequate type 1 immune response to control *P. yoelii* proliferation and increased survival, while inhibition of only IL-10 or only TGF-β promoted parasite clearance, but could not avoid lethality (61). In conclusion, IL-10 may be beneficial to avoid immunopathology, but it may also inhibit anti-parasitic responses that are crucial to control parasite growth.

Our results suggest that NK cells may have a regulatory phenotype in the recovery of MA-ARDS as shown by an increased expression of *Il10* in the NK cells, explaining the observed effect of NK cells in the resolution. We demonstrated an increased global pulmonary *Il10* expression at 9 dpi in *Pb*NK65-infected mice, which was independent of antimalarial treatment and NK cells, suggesting that the majority of IL-10 in the lungs is produced by other leukocyte subsets. Global inhibition of the IL-10/IL-10R axis had no effect on the resolution of MA-ARDS nor on the decrease in parasitemia upon antimalarial treatment, suggesting that pro-resolving pathways may be more important than IL-10-mediated anti-inflammatory pathways in the resolution of MA-ARDS. Further investigations are needed to decipher through which mechanisms NK cells promote the resolution of MA-ARDS.

In conclusion, by using two different approaches, NK cells were found to contribute to the recovery from MA-ARDS during antimalarial treatment, without affecting the development of MA-ARDS. This was paralleled by high *Il10* expression by the NK cells, suggesting a potent regulatory phenotype. However, inhibition of the IL-10/IL-10R axis had no effect on the resolution of MA-ARDS nor on the decrease in parasitemia upon antimalarial treatment, suggesting that there is probably a combination of factors at play in the protective role of NK cells.

## Data Availability

The original contributions presented in the study are included in the article/**Supplementary Material**, further inquiries can be directed to the corresponding author.
