# Supplementary material for: NK cells contribute to the resolution of experimental malaria-associated acute respiratory distress syndrome after antimalarial treatment
Source: Front Immunol. 2024 Sep 17;15:1433904. doi: 10.3389/fimmu.2024.1433904 (PMC11442241; doi:10.3389/fimmu.2024.1433904)
Supplement: Supplementary file 1 [file DataSheet1.docx]

# Supplementary Figures


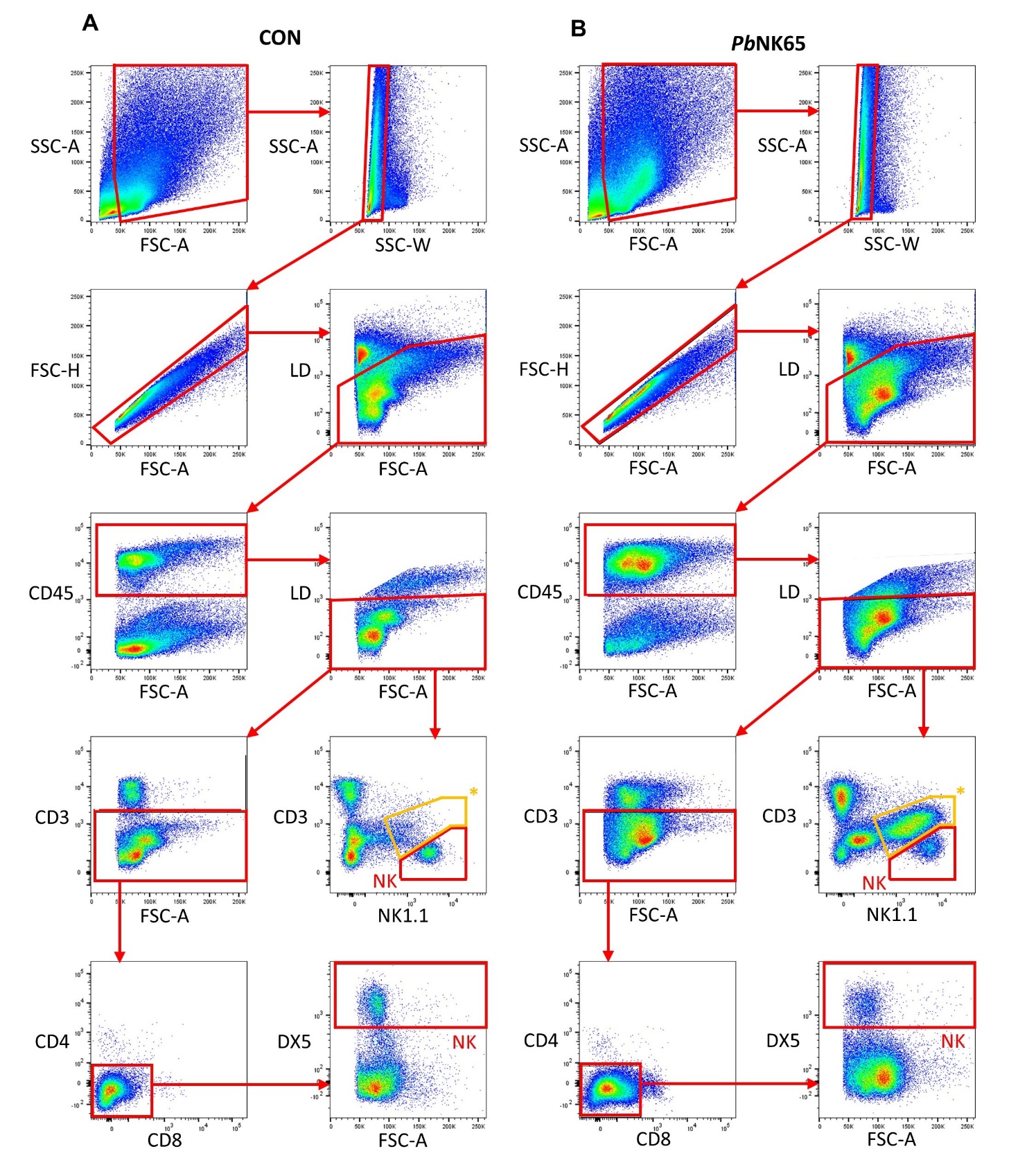


**Supplementary Figure 1. Flow cytometry gating strategy for NK cells.**

Pulmonary and splenic cells were isolated and stained to perform flow cytometry. Red blood cells, debris and doublets of cells were excluded and all live cells (LD^-^) were gated. The leukocytes were identified as CD45^+^. In case of lung samples, autofluorescent alveolar macrophages were excluded by plotting LD versus FSC-A as those cells otherwise result in false positive staining. Representative gatings of pulmonary cells isolated from an uninfected control and of a *Pb*NK65-infected C57BL/6 mouse at 9 dpi according to protocol 2 are shown. Two alternative gating strategies for NK cells are shown based on either NK1.1 or DX5. Note that the CD3^int^ NK1.1^+^ cells (*; yellow gate) appearing in the infected mice are monocytes and macrophages that bind the anti-NK1.1 antibody in an aspecific way, and are thus not NK cells. This was elaborately shown in our recent study, including by comparison with the corresponding isotype antibody control, by further immunophenotyping and by microscopy of sorted cells (44). The NK cells are indicated in the red CD3^-^ NK1.1^+^ gate and in the red CD3^-^ CD4^-^ CD8^-^ DX5^+^ gate. LD, live dead.


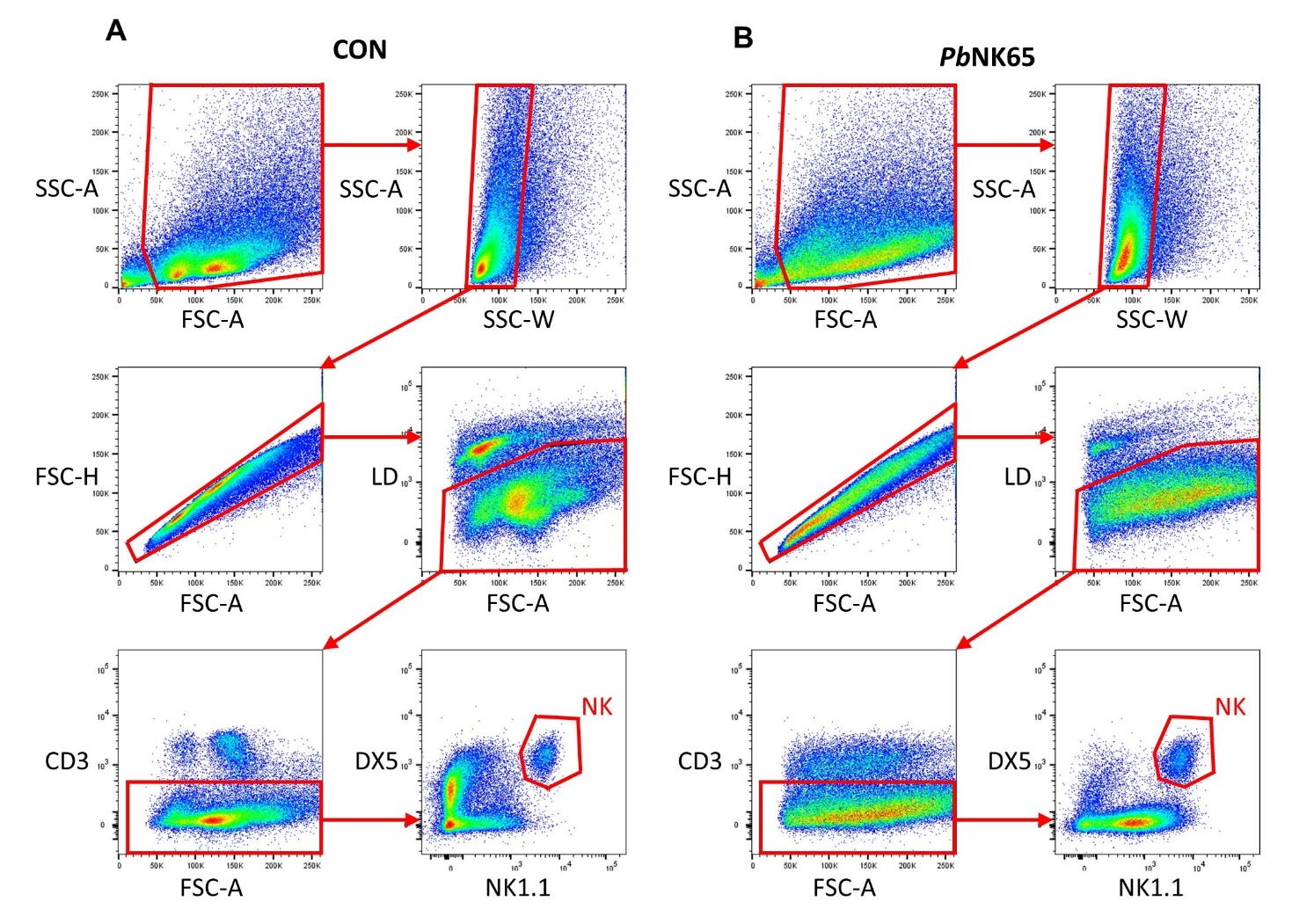


**Supplementary Figure 2. Fluorescence-activated cell sorting gating for NK cells.**

Pulmonary cells were isolated according to protocol 2 and stained to perform fluorescence-activated cell sorting. Red blood cells, debris and doublets of cells were excluded and all live cells (LD^-^) were gated. NK cells were sorted as CD3^-^ NK1.1^+^ DX5^+^ (Red gate). Representative gatings of an uninfected control and of a *Pb*NK65-infected C57BL/6 mouse at 9 dpi are shown. LD, live dead.


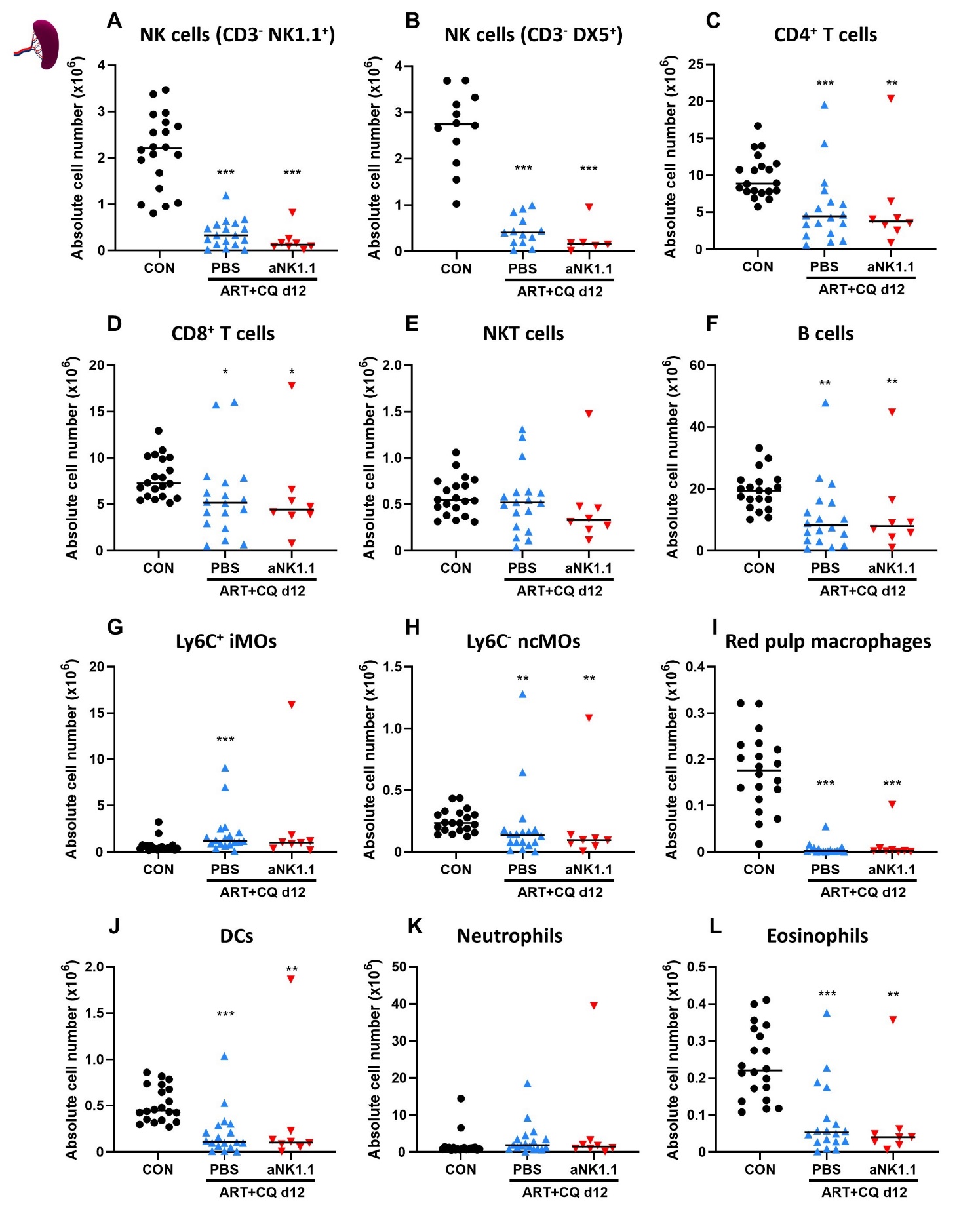


**Supplementary Figure 3. NK cell depletion has no effect on other lymphoid and myeloid cell populations present in the spleen of antimalarial-treated, *Pb*NK65-infected C57BL/6 mice.**

C57BL/6 mice were infected with *Pb*NK65. Daily treatment from 8 until 12 dpi with 10 mg/kg artesunate + 30 mg/kg chloroquine (ART+CQ). At 6 and 9 dpi, mice were injected i.p. with 500 µg of anti-NK1.1 (PK136) depletion antibodies or PBS. Mice were dissected at 12 dpi. Splenic cells were and flow cytometry was performed. The absolute number of (A-B) NK cells (A: CD45^+^ CD3^-^ NK1.1^+^; B: CD45^+^ CD3^-^ DX5^+^), (C) CD4^+^ T cells (CD45^+^ CD3^+^ NK1.1^-^ CD4^+^), (D) CD8^+^ T cells (CD45^+^ CD3^+^ NK1.1^-^ CD8^+^), (E) NKT cells (CD45^+^ CD3^+^ NK1.1^+^), (F) B cells (CD45^+^ CD3^-^ NK1.1^-^ B220^+^), (G) Ly6C^+^ inflammatory monocytes (iMOs; CD45^+^ Lin^-^ SiglecF^-^ Ly6G^-^ CD11b^hi^ MHCII^-^ Ly6C^+^), (H) Ly6C^-^ non-classical monocytes (ncMOs; CD45^+^ Lin^-^ SiglecF^-^ Ly6G^-^ CD11b^hi^ MHCII^-^ Ly6C^-^), (I) Red pulp macrophages (CD45^+^ Lin^-^ Ly6G^-^ SiglecF^-^ CD11b^-^ F4/80^+^), (J) Dendritic cells (DCs; CD45^+^ Lin^-^ MHCII^+^ CD11c ^+^), (H) CD11b^+^ dendritic cells (CD11b^+^ DCs; CD45^+^ Lin^-^ SiglecF^-^ MHCII^+^ CD11c^+^ CD11b^+^ CD24^+^ CD64^-^), (K) Neutrophils (CD45^+^ Lin^-^ CD11b^+^ Ly6G^+^) and (L) Eosinophils (CD45^+^ SiglecF^+^ CD11c^-^) in the spleen were calculated. For the myeloid cell gating, only Lineage-negative (Lin^-^) cells were selected based on CD3, CD19 and NK1.1. Data from two (B) or three (A, C-L) experiments. Each symbol represents data of an individual mouse. n = 12-20 for CON, n = 13-18 for ART+CQ PBS, n = 6-8 for ART+CQ aNK1.1. The non-parametric Mann-Whitney U test followed by the Holm-Bonferroni correction was used to determine significance between all groups. P-values were indicated as follows: *p<0.05, **p<0.01, ***p<0.001. Median in each group was indicated by a horizontal black line, unless indicated otherwise. Statistical differences compared to the uninfected control group are indicated with asterisk above the individual data sets and horizontal lines with asterisk on top indicate significant differences between groups.


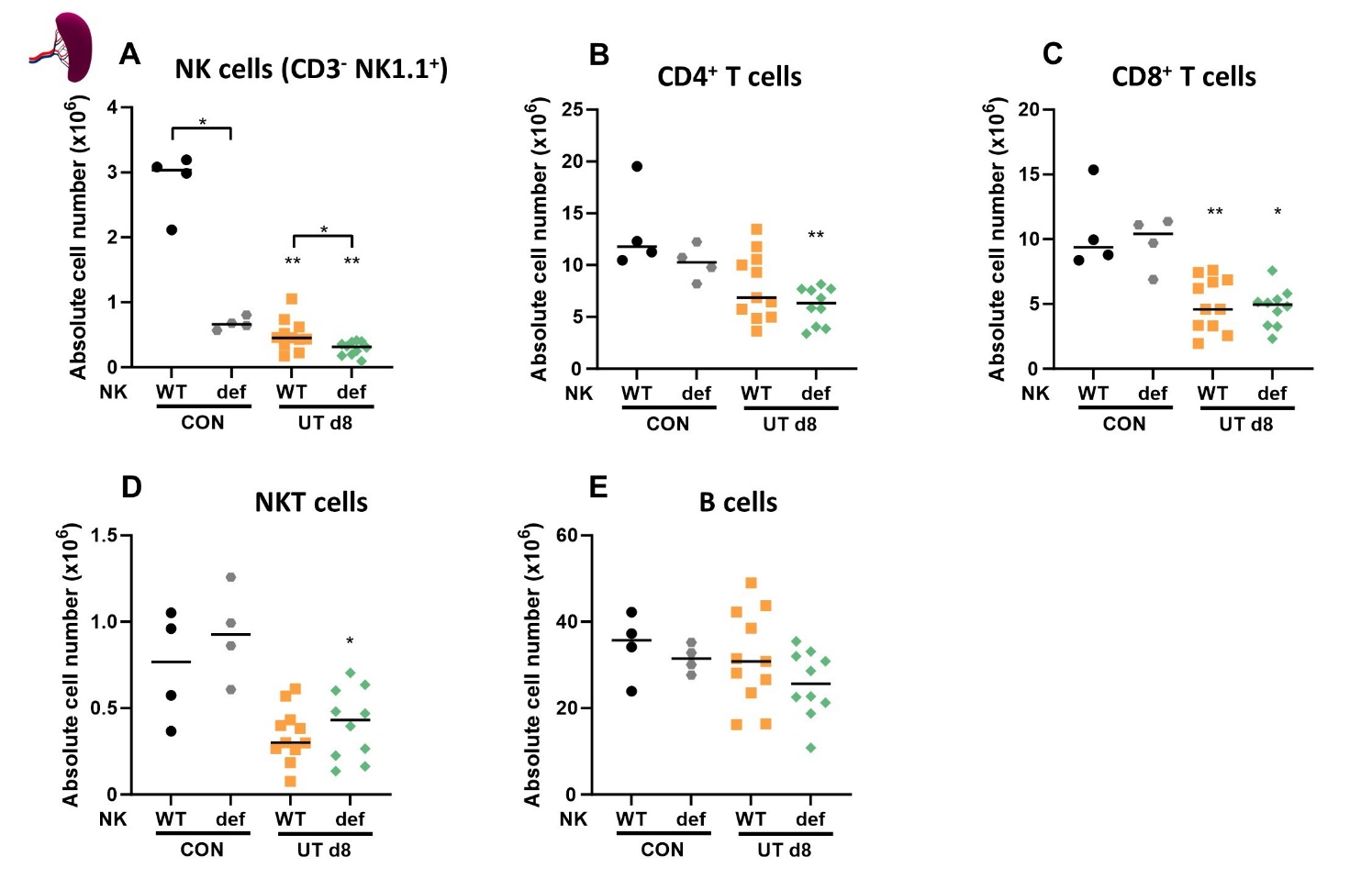


**Supplementary Figure 4.** **NK cell deficiency had no effect on other splenic lymphocyte populations.**

NK cell-deficient (Ncr1-iCre^Tg/WT^ ROSA-DTA^Tg/WT^) and non-deficient (Ncr1-iCre^Tg/WT^ ROSA-DTA^WT/WT^, Ncr1-iCre^WT/WT^ ROSA-DTA^Tg/WT^, Ncr1-iCre^WT/WT^ ROSA-DTA^WT/WT^) C57BL/6 mice were infected with *Pb*NK65. Mice were dissected at 8 dpi. Splenic cells were isolated and flow cytometry was performed. The absolute number of (A) NK cells (CD45^+^ CD3^-^ NK1.1^+^), (B) CD4^+^ T cells (CD45^+^ CD3^+^ NK1.1^-^ CD4^+^), (C) CD8^+^ T cells (CD45^+^ CD3^+^ NK1.1^-^ CD8^+^), (D) NKT cells (CD45^+^ CD3^+^ NK1.1^+^) and (E) B cells (CD45^+^ CD3^-^ NK1.1^-^ B220^+^) in the lungs were calculated. Data from two experiments. Each symbol represents data of an individual mouse. n = 4 for CON NK^WT^ and CON NK^def^, n = 11 for UT NK^WT^, n = 10 for UT NK^def^. The non-parametric Mann-Whitney U test followed by the Holm-Bonferroni correction was used to determine significance between each condition for the NK cell-deficient mice and between each condition for the non-deficient mice and between the NK cell-deficient and non-deficient mice within each condition. P-values were indicated as follows: *p<0.05, **p<0.01, ***p<0.001. Median in each group was indicated by a horizontal black line, unless indicated otherwise. Statistical differences compared to the appropriate uninfected control group are indicated with asterisk above the individual data sets and horizontal lines with asterisk on top indicate significant differences between groups.


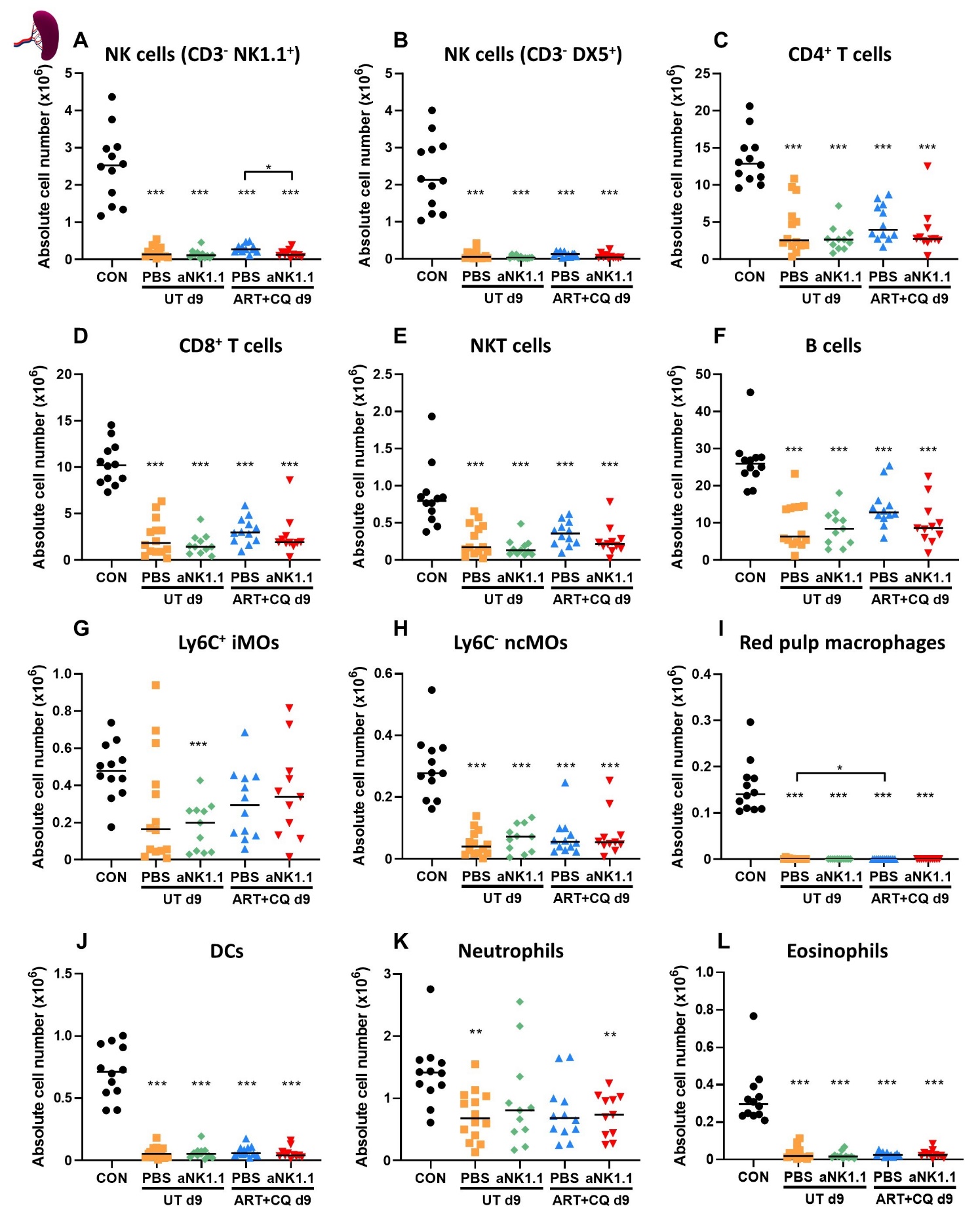


**Supplementary Figure 5. NK cell depletion has no effect on other lymphoid and myeloid cell populations present in the spleen at 9 dpi.**

C57BL/6 mice were infected with *Pb*NK65. Mice were treated at 8 dpi with 10 mg/kg artesunate + 30 mg/kg chloroquine (ART+CQ). At 6 dpi, mice were injected i.p. with 500 µg of anti-NK1.1 (PK136) depletion antibodies or PBS. Mice were dissected at 9 dpi. Splenic cells were isolated and flow cytometry was performed. The absolute number of (A-B) NK cells (A: CD45^+^ CD3^-^ NK1.1^+^; B: CD45^+^ CD3^-^ DX5^+^), (C) CD4^+^ T cells (CD45^+^ CD3^+^ NK1.1^-^ CD4^+^), (D) CD8^+^ T cells (CD45^+^ CD3^+^ NK1.1^-^ CD8^+^), (E) NKT cells (CD45^+^ CD3^+^ NK1.1^+^), (F) B cells (CD45^+^ CD3^-^ NK1.1^-^ B220^+^), (G) Ly6C^+^ inflammatory monocytes (iMOs; CD45^+^ Lin^-^ SiglecF^-^ Ly6G^-^ CD11b^hi^ MHCII^-^ Ly6C^+^), (H) Ly6C^-^ non-classical monocytes (ncMOs; CD45^+^ Lin^-^ SiglecF^-^ Ly6G^-^ CD11b^hi^ MHCII^-^ Ly6C^-^), (I) Red pulp macrophages (CD45^+^ Lin^-^ Ly6G^-^ SiglecF^-^ CD11b^-^ F4/80^+^), (K) Dendritic cells (DCs; CD45^+^ Lin^-^ MHCII^+^ CD11c^+^), (K) Neutrophils (CD45^+^ Lin^-^ CD11b^+^ Ly6G^+^) and (L) Eosinophils (CD45^+^ SiglecF^+^ CD11c^-^) in the spleen were calculated. For the myeloid cell gating, only lineage-negative (Lin^-^) cells were selected based on CD3, CD19 and NK1.1. Data from three experiments. Each symbol represents data of an individual mouse. n = 12 for CON, n = 14 for UT PBS, n = 11 for UT aNK1.1, n = 12 for ART+CQ PBS, n = 11 for ART+CQ aNK1.1. The non-parametric Mann-Whitney U test followed by the Holm-Bonferroni correction was used to determine significance between all groups, except for the comparison untreated (UT) PBS with antimalarial drug-treated (ART+CQ) anti-NK1.1 and the comparison UT anti-NK1.1 with ART+CQ PBS. P-values were indicated as follows: *p<0.05, **p<0.01, ***p<0.001. Median in each group was indicated by a horizontal black line, unless indicated otherwise. Statistical differences compared to the uninfected control group are indicated with asterisk above the individual data sets and horizontal lines with asterisk on top indicate significant differences between groups.
