# Supplementary material for: NK cells contribute to the resolution of experimental malaria-associated acute respiratory distress syndrome after antimalarial treatment
Source: Front Immunol. 2024 Sep 17;15:1433904. doi: 10.3389/fimmu.2024.1433904 (PMC11442241; doi:10.3389/fimmu.2024.1433904)
Supplement: Supplementary file 2 [file Table1.pdf]

Supplementary Table 1. SNP analysis

Index	Name	Chr	Position	SNPs that differentiate between C57BL/6NTac and C57BL/6J on the Mouse Genome Scanning Panel	Genotyping Result								Congenic Result					
					C57BL/6NTac-3_SNP Ref Sample.GType	C57BL/6Jax-3_SNP Ref Sample.GType	Ncr1-iCre$^{Tg/WT}$ ROSA-DTA$^{WT/WT}$	Ncr1-iCre$^{WT/WT}$ ROSA-DTA$^{Tg/WT}$	Ncr1-iCre$^{Tg/WT}$ ROSA-DTA$^{WT/WT}$	Ncr1-iCre$^{Tg/WT}$ ROSA-DTA$^{WT/WT}$	Ncr1-iCre$^{WT/WT}$ ROSA-DTA$^{WT/WT}$	Ncr1-iCre$^{Tg/WT}$ ROSA-DTA$^{WT/WT}$	Ncr1-iCre$^{Tg/WT}$ ROSA-DTA$^{WT/WT}$	Ncr1-iCre$^{WT/WT}$ ROSA-DTA$^{Tg/WT}$	Ncr1-iCre$^{Tg/WT}$ ROSA-DTA$^{WT/WT}$	Ncr1-iCre$^{Tg/WT}$ ROSA-DTA$^{WT/WT}$	Ncr1-iCre$^{WT/WT}$ ROSA-DTA$^{WT/WT}$	Ncr1-iCre$^{Tg/WT}$ ROSA-DTA$^{WT/WT}$
272	CH0MM1	0	24466918		AA	AA	AA	AA	AA	AA	AA	AA	1	1	1	1	1	1
1254	CH1MM1	1	3197400		AA	AA	AA	AA	AA	AA	AA	AA	1	1	1	1	1	1
1819	CH1MM2	1	3492195		AA	AA	AA	AA	AA	AA	AA	AA	1	1	1	1	1	1
321	CH1MM3	1	4526088		AA	AA	AA	AA	AA	AA	AA	AA	1	1	1	1	1	1
1987	CH1MM4	1	5602036		AA	AA	AA	AA	AA	AA	AA	AA	1	1	1	1	1	1
322	CH1MM5	1	5906465		AA	AA	AA	AA	AA	AA	AA	AA	1	1	1	1	1	1
323	CH1MM6GM1	1	6655964		AA	AA	AA	AA	AA	AA	AA	AA	1	1	1	1	1	1
1259	CH1MM7	1	8083448		BB	BB	BB	BB	BB	BB	BB	BB	1	1	1	1	1	1
1448	CH1MM8	1	9141039		BB	BB	BB	BB	BB	BB	BB	BB	1	1	1	1	1	1
1810	CH1MM9	1	11106394		BB	BB	BB	BB	BB	BB	BB	BB	1	1	1	1	1	1
1491	CH1MM10	1	11371737		AA	AA	AA	AA	AA	AA	AA	AA	1	1	1	1	1	1
324	CH1MM11	1	12981709		BB	BB	BB	BB	BB	BB	BB	BB	1	1	1	1	1	1
1183	CH1MM12	1	16448703		AA	AA	AA	AA	AA	AA	AA	AA	1	1	1	1	1	1
325	CH1MM13	1	17512198		BB	BB	BB	BB	BB	BB	BB	BB	1	1	1	1	1	1
257	CH1MM14	1	19040122		AA	AA	AA	AA	AA	AA	AA	AA	1	1	1	1	1	1
1100	CH1MM15	1	19291760		BB	BB	BB	BB	BB	BB	BB	BB	1	1	1	1	1	1
326	CH1MM16	1	19680103		AA	AA	AA	AA	AA	AA	AA	AA	1	1	1	1	1	1
1972	CH1MM17	1	20994482		BB	BB	BB	BB	BB	BB	BB	BB	1	1	1	1	1	1
1716	CH1MM18	1	22977896		AA	AA	AA	AA	AA	AA	AA	AA	1	1	1	1	1	1
270	CH1MM19	1	25471405		AA	AA	AA	AA	AA	AA	AA	AA	1	1	1	1	1	1
1712	CH1MM20	1	26136349		AA	AA	AA	AA	AA	AA	AA	AA	1	1	1	1	1	1
1224	CH1MM21	1	27510356		AA	AA	AA	AA	AA	AA	AA	AA	1	1	1	1	1	1
10	CH1MM22	1	28516388		AA	AA	AA	AA	AA	AA	AA	AA	1	1	1	1	1	1
327	CH1MM23	1	28549433		BB	BB	BB	BB	BB	BB	BB	BB	1	1	1	1	1	1
1313	CH1MM24	1	29990732		AA	AA	AA	AA	AA	AA	AA	AA	1	1	1	1	1	1
328	CH1MM25	1	30024577		AA	AA	AA	AA	AA	AA	AA	AA	1	1	1	1	1	1
1642	CH1MM26	1	30030567		AA	AA	AA	AA	AA	AA	AA	AA	1	1	1	1	1	1
1529	CH1MM27	1	31102378		BB	BB	BB	BB	BB	BB	BB	BB	1	1	1	1	1	1
1391	CH1MM28	1	32799125		BB	BB	BB	BB	BB	BB	BB	BB	1	1	1	1	1	1
1242	CH1MM29	1	34414162		AA	AA	AA	AA	AA	AA	AA	AA	1	1	1	1	1	1
1255	CH1MM30	1	36198091		BB	BB	BB	BB	BB	BB	BB	BB	1	1	1	1	1	1
1637	CH1MM31	1	37990570		BB	BB	BB	BB	BB	BB	BB	BB	1	1	1	1	1	1
1596	CH1MM32	1	39320203		BB	BB	BB	BB	BB	BB	BB	BB	1	1	1	1	1	1
143	CH1MM33	1	40297949		BB	BB	BB	BB	BB	BB	BB	BB	1	1	1	1	1	1
329	CH1MM34	1	41262551		BB	BB	BB	BB	BB	BB	BB	BB	1	1	1	1	1	1
144	CH1MM35	1	41423174		BB	BB	BB	BB	BB	BB	BB	BB	1	1	1	1	1	1
330	CH1MM36	1	42789902		AA	AA	AA	AA	AA	AA	AA	AA	1	1	1	1	1	1
958	CH1MM37	1	44001168		BB	BB	BB	BB	BB	BB	BB	BB	1	1	1	1	1	1
28	CH1MM38	1	44668113		AA	AA	AA	AA	AA	AA	AA	AA	1	1	1	1	1	1
331	CH1MM39	1	46277649		AA	AA	AA	AA	AA	AA	AA	AA	1	1	1	1	1	1
1046	CH1MM40BLK4	1	46932893	X	BB	AA	AA	AA	AA	AA	AA	AA	1	1	1	1	1	1
332	CH1MM41	1	47845326		AA	AA	AA	AA	AA	AA	AA	AA	1	1	1	1	1	1
29	CH1MM42	1	49807741		BB	BB	BB	BB	BB	BB	BB	BB	1	1	1	1	1	1
30	CH1MM43	1	49993068		BB	BB	BB	BB	BB	BB	BB	BB	1	1	1	1	1	1
1502	CH1MM44	1	50613798		BB	BB	BB	BB	BB	BB	BB	BB	1	1	1	1	1	1
333	CH1MM45	1	51771264		BB	BB	BB	BB	BB	BB	BB	BB	1	1	1	1	1	1
334	CH1MM46	1	52159056		AA	AA	AA	AA	AA	AA	AA	AA	1	1	1	1	1	1
283	CH1MM47GM2-BM	1	52665170		AA	AA	AA	AA	AA	AA	AA	AA	1	1	1	1	1	1
1721	CH1MM48	1	53853346		BB	BB	BB	BB	BB	BB	BB	BB	1	1	1	1	1	1
1945	CH1MM49	1	54482766		AA	AA	AA	AA	AA	AA	AA	AA	1	1	1	1	1	1
1492	CH1MM50	1	55942220		BB	BB	BB	BB	BB	BB	BB	BB	1	1	1	1	1	1
1659	CH1MM51	1	58438091		BB	BB	BB	BB	BB	BB	BB	BB	1	1	1	1	1	1
335	CH1MM52	1	58775689		AA	AA	AA	AA	AA	AA	AA	AA	1	1	1	1	1	1
1748	CH1MM53	1	59624742		AA	AA	AA	AA	AA	AA	AA	AA	1	1	1	1	1	1
1897	CH1MM54	1	60126995		BB	BB	BB	BB	BB	BB	BB	BB	1	1	1	1	1	1
1452	CH1MM55	1	61943426		AA	AA	AA	AA	AA	AA	AA	AA	1	1	1	1	1	1
1068	CH1MM56	1	62562701	X	BB	AA	AB	AA	AA	BB	AB	BB	0,5	1	1	0	0,5	0
1874	CH1MM57	1	63649681		BB	BB	BB	BB	BB	BB	BB	BB	1	1	1	1	1	1
11	CH1MM58	1	64798968		BB	BB	BB	BB	BB	BB	BB	BB	1	1	1	1	1	1
1956	CH1MM59	1	65272783		BB	BB	BB	BB	BB	BB	BB	BB	1	1	1	1	1	1
336	CH1MM60	1	66810982		AA	AA	AA	AA	AA	AA	AA	AA	1	1	1	1	1	1
1841	CH1MM61	1	68917890		BB	BB	BB	BB	BB	BB	BB	BB	1	1	1	1	1	1
337	CH1MM62	1	69117243		BB	BB	BB	BB	BB	BB	BB	BB	1	1	1	1	1	1
1860	CH1MM63	1	69408967		AA	AA	AA	AA	AA	AA	AA	AA	1	1	1	1	1	1
1730	CH1MM64	1	72377589		AA	AA	AA	AA	AA	AA	AA	AA	1	1	1	1	1	1
338	CH1MM65	1	73407862		BB	BB	BB	BB	BB	BB	BB	BB	1	1	1	1	1	1
1435	CH1MM66	1	74900046		AA	AA	AA	AA	AA	AA	AA	AA	1	1	1	1	1	1
1902	CH1MM67	1	75362320		AA	AA	AA	AA	AA	AA	AA	AA	1	1	1	1	1	1
339	CH1MM68	1	76832354		AA	AA	AA	AA	AA	AA	AA	AA	1	1	1	1	1	1
260	CH1MM69	1	76984491		BB	BB	BB	BB	BB	BB	BB	BB	1	1	1	1	1	1
145	CH1MM70	1	78359030		BB	BB	BB	BB	BB	BB	BB	BB	1	1	1	1	1	1
340	CH1MM71	1	79202787		BB	BB	BB	BB	BB	BB	BB	BB	1	1	1	1	1	1
254	CH1MM72	1	80839073		BB	BB	BB	BB	BB	BB	BB	BB	1	1	1	1	1	1
341	CH1MM73	1	81235702		BB	BB	BB	BB	BB	BB	BB	BB	1	1	1	1	1	1
1003	CH1MM74	1	82651571		AA	AA	AA	AA	AA	AA	AA	AA	1	1	1	1	1	1
146	CH1MM75	1	82668689		AA	AA	AA	AA	AA	AA	AA	AA	1	1	1	1	1	1
147	CH1MM76	1	85664769		AA	AA	AA	AA	AA	AA	AA	AA	1	1	1	1	1	1
1493	CH1MM77	1	85865441		BB	BB	BB	BB	BB	BB	BB	BB	1	1	1	1	1	1
2003	CH1MM78	1	87190477		BB	BB	BB	BB	BB	BB	BB	BB	1	1	1	1	1	1
1737	CH1MM79	1	88494443		AA	AA	AA	AA	AA	AA	AA	AA	1	1	1	1	1	1
342	CH1MM80	1	91575836		BB	BB	BB	BB	BB	BB	BB	BB	1	1	1	1	1	1
1818	CH1MM81	1	93319361		AA	AA	AA	AA	AA	AA	AA	AA	1	1	1	1	1	1
1702	CH1MM82	1	95497940		AA	AA	AA	AA	AA	AA	AA	AA	1	1	1	1	1	1
1652	CH1MM83	1	95625917		AA	AA	AA	AA	AA	AA	AA	AA	1	1	1	1	1	1
1520	CH1MM84	1	96875139		BB	BB	BB	BB	BB	BB	BB	BB	1	1	1	1	1	1
343	CH1MM85	1	97677389		AA	AA	AA	AA	AA	AA	AA	AA	1	1	1	1	1	1
344	CH1MM86	1	99797518		AA	AA	AA	AA	AA	AA	AA	AA	1	1	1	1	1	1
1916	CH1MM87	1	101749052		BB	BB	BB	BB	BB	BB	BB	BB	1	1	1	1	1	1
24	CH1MM88	1	103251925		BB	BB	BB	BB	BB	BB	BB	BB	1	1	1	1	1	1
345	CH1MM89	1	103459296		AA	AA	AA	AA	AA	AA	AA	AA	1	1	1	1	1	1
1151	CH1MM90	1	104472398		BB	BB	BB	BB	BB	BB	BB	BB	1	1	1	1	1	1
1677	CH1MM91	1	105335135		BB	BB	BB	BB	BB	BB	BB	BB	1	1	1	1	1	1
25	CH1MM92	1	105423103		BB	BB	BB	BB	BB	BB	BB	BB	1	1	1	1	1	1
346	CH1MM93	1	106831730		AA	AA	AA	AA	AA	AA	AA	AA	1	1	1	1	1	1
1264	CH1MM94	1	109634975		BB	BB	BB	BB	BB	BB	BB	BB	1	1	1	1	1	1
347	CH1MM95	1	110616212		AA	AA	AA	AA	AA	AA	AA	AA	1	1	1	1	1	1
1514	CH1MM96	1	111780707		AA	AA	AA	AA	AA	AA	AA	AA	1	1	1	1	1	1
348	CH1MM97	1	113899609		AA	AA	AA	AA	AA	AA	AA	AA	1	1	1	1	1	1
1977	CH1MM98	1	115462613		BB	BB	BB	BB	BB	BB	BB	BB	1	1	1	1	1	1
262	CH1MM99	1	115751113		BB	BB	BB	BB	BB	BB	BB	BB	1	1	1	1	1	1
1203	CH1MM100	1	117379659		BB	BB	BB	BB	BB	BB	BB	BB	1	1	1	1	1	1
1310	CH1MM101	1	118500635		BB	BB	BB	BB	BB	BB	BB	BB	1	1	1	1	1	1
1230	CH1MM102	1	120380872		BB	BB	BB	BB	BB	BB	BB	BB	1	1	1	1	1	1
349	CH1MM103GM3	1	122010714		BB	BB	BB	BB	BB	BB	BB	BB	1	1	1	1	1	1
1455	CH1MM104	1	122796548		AA	AA	AA	AA	AA	AA	AA	AA	1	1	1	1	1	1
1302	CH1MM105	1	123642529		BB	BB	BB	BB	BB	BB	BB	BB	1	1	1	1	1	1
1531	CH1MM106	1	125475941		BB	BB	BB	BB	BB	BB	BB	BB	1	1	1	1	1	1
350	CH1MM107	1	126400694		AA	AA	AA	AA	AA	AA	AA	AA	1	1	1	1	1	1

Taconic Key

Symbol	Definition
AA	Homozygous for Allele1
AB	Heterozygous 1/2
BB	Homozygous for Allele2
1	Homozygous for C57BL/6J SNP
0,5	Heterozygous for C57BL/6J SNP and non-C57BL/6J SNP
0	Homozygous for non-C57BL/6J SNP
NC=no call	
Highlighted region indicates the region 25 MBp on either side of the known chromosomal modification	

1773	CH1MM108	1	126460218		BB	BB	BB	BB	BB	BB	BB	BB	1	1	1	1	1	1
351	CH1MM109	1	128094625		BB	BB	BB	BB	BB	BB	BB	BB	1	1	1	1	1	1
1899	CH1MM110	1	128165659		AA	AA	AA	AA	AA	AA	AA	AA	1	1	1	1	1	1
1342	CH1MM111	1	130962369		BB	BB	BB	BB	BB	BB	BB	BB	1	1	1	1	1	1
1507	CH1MM112	1	131376437		AA	AA	AA	AA	AA	AA	AA	AA	1	1	1	1	1	1
352	CH1MM113	1	134099450		AA	AA	AA	AA	AA	AA	AA	AA	1	1	1	1	1	1
1997	CH1MM114	1	135044501		AA	AA	AA	AA	AA	AA	AA	AA	1	1	1	1	1	1
1831	CH1MM115	1	137268795		AA	AA	AA	AA	AA	AA	AA	AA	1	1	1	1	1	1
1801	CH1MM116	1	137529520		AA	AA	AA	AA	AA	AA	AA	AA	1	1	1	1	1	1
148	CH1MM117	1	138089211		AA	AA	AA	AA	AA	AA	AA	AA	1	1	1	1	1	1
353	CH1MM118	1	139114404		BB	BB	BB	BB	BB	BB	BB	BB	1	1	1	1	1	1
136	CH1MM119BLK8	1	139200000	X	AA	BB	BB	BB	BB	BB	BB	BB	1	1	1	1	1	1
354	CH1MM120	1	140472264		AA	AA	AA	AA	AA	AA	AA	AA	1	1	1	1	1	1
1935	CH1MM121	1	142470600		AA	AA	AA	AA	AA	AA	AA	AA	1	1	1	1	1	1
355	CH1MM122BLK9	1	142911628	X	AA	BB	BB	BB	BB	BB	BB	BB	1	1	1	1	1	1
356	CH1MM123	1	144012938		AA	AA	AA	AA	AA	AA	AA	AA	1	1	1	1	1	1
1646	CH1MM124	1	144695531		AA	AA	AA	AA	AA	AA	AA	AA	1	1	1	1	1	1
1193	CH1MM125	1	145088382		BB	BB	BB	BB	BB	BB	BB	BB	1	1	1	1	1	1
357	CH1MM126	1	146776886		AA	AA	AA	AA	AA	AA	AA	AA	1	1	1	1	1	1
1982	CH1MM127	1	148913234		BB	BB	BB	BB	BB	BB	BB	BB	1	1	1	1	1	1
358	CH1MM128	1	150586221		AA	AA	AA	AA	AA	AA	AA	AA	1	1	1	1	1	1
1953	CH1MM129	1	150967309		BB	BB	BB	BB	BB	BB	BB	BB	1	1	1	1	1	1
26	CH1MM130	1	152747565		BB	BB	BB	BB	BB	BB	BB	BB	1	1	1	1	1	1
359	CH1MM131	1	154187910		BB	BB	BB	BB	BB	BB	BB	BB	1	1	1	1	1	1
1070	CH1MM132GM4	1	154676268		BB	BB	BB	BB	BB	BB	BB	BB	1	1	1	1	1	1
2040	CH1MM133	1	156044150		AA	AA	AA	AA	AA	AA	AA	AA	1	1	1	1	1	1
1222	CH1MM134	1	156461112		AA	AA	AA	AA	AA	AA	AA	AA	1	1	1	1	1	1
1467	CH1MM135	1	157158100		BB	BB	BB	BB	BB	BB	BB	BB	1	1	1	1	1	1
1947	CH1MM136	1	157898706		AA	AA	AA	AA	AA	AA	AA	AA	1	1	1	1	1	1
1296	CH1MM137	1	160294867		BB	BB	BB	BB	BB	BB	BB	BB	1	1	1	1	1	1
360	CH1MM138	1	162037129		AA	AA	AA	AA	AA	AA	AA	AA	1	1	1	1	1	1
1263	CH1MM139	1	162261064		AA	AA	AA	AA	AA	AA	AA	AA	1	1	1	1	1	1
2041	CH1MM140	1	164012960		BB	BB	BB	BB	BB	BB	BB	BB	1	1	1	1	1	1
1389	CH1MM141	1	164408862		BB	BB	BB	BB	BB	BB	BB	BB	1	1	1	1	1	1
361	CH1MM142	1	166263327		BB	BB	BB	BB	BB	BB	BB	BB	1	1	1	1	1	1
1962	CH1MM143	1	167289594		BB	BB	BB	BB	BB	BB	BB	BB	1	1	1	1	1	1
2007	CH1MM144	1	167367806		BB	BB	BB	BB	BB	BB	BB	BB	1	1	1	1	1	1
362	CH1MM145	1	169381101		BB	BB	BB	BB	BB	BB	BB	BB	1	1	1	1	1	1
2011	CH1MM146	1	171224975		BB	BB	BB	BB	BB	BB	BB	BB	1	1	1	1	1	1
1994	CH1MM147	1	171312251		BB	BB	BB	BB	BB	BB	BB	BB	1	1	1	1	1	1
318	CH1MM148	1	172206556		AA	AA	AA	AA	AA	AA	AA	AA	1	1	1	1	1	1
1393	CH1MM149	1	172614886		BB	BB	BB	BB	BB	BB	BB	BB	1	1	1	1	1	1
363	CH1MM150	1	174277920		BB	BB	BB	BB	BB	BB	BB	BB	1	1	1	1	1	1
1169	CH1MM151	1	174667766		AA	AA	AA	AA	AA	AA	AA	AA	1	1	1	1	1	1
1353	CH1MM152	1	176145762		AA	AA	AA	AA	AA	AA	AA	AA	1	1	1	1	1	1
364	CH1MM153	1	177399967		AA	AA	AA	AA	AA	AA	AA	AA	1	1	1	1	1	1
27	CH1MM154	1	178482360		BB	BB	BB	BB	BB	BB	BB	BB	1	1	1	1	1	1
365	CH1MM155	1	179633768		BB	BB	BB	BB	BB	BB	BB	BB	1	1	1	1	1	1
320	CH1MM156	1	180675247		AA	AA	AA	AA	AA	AA	AA	AA	1	1	1	1	1	1
366	CH1MM157	1	181495101		BB	BB	BB	BB	BB	BB	BB	BB	1	1	1	1	1	1
367	CH1MM158	1	182175880		BB	BB	BB	BB	BB	BB	BB	BB	1	1	1	1	1	1
2039	CH1MM159	1	182510631		BB	BB	BB	BB	BB	BB	BB	BB	1	1	1	1	1	1
1703	CH1MM160	1	183197685		BB	BB	BB	BB	BB	BB	BB	BB	1	1	1	1	1	1
241	CH1MM161	1	186531848		AA	AA	AA	AA	AA	AA	AA	AA	1	1	1	1	1	1
1164	CH1MM162	1	188355284		AA	AA	AA	AA	AA	AA	AA	AA	1	1	1	1	1	1
368	CH1MM163	1	190164592		BB	BB	BB	BB	BB	BB	BB	BB	1	1	1	1	1	1
1284	CH1MM164	1	192397145		AA	AA	AA	AA	AA	AA	AA	AA	1	1	1	1	1	1
932	CH1MM165	1	192857724		BB	BB	BB	BB	BB	BB	BB	BB	1	1	1	1	1	1
931	CH1MM166	1	192893335		AA	AA	AA	AA	AA	AA	AA	AA	1	1	1	1	1	1
940	CH1MM167	1	193134674		AA	AA	AA	AA	AA	AA	AA	AA	1	1	1	1	1	1
369	CH1MM168	1	194054190		BB	BB	BB	BB	BB	BB	BB	BB	1	1	1	1	1	1
261	CH1MM169	1	195161421		BB	BB	BB	BB	BB	BB	BB	BB	1	1	1	1	1	1
1436	CH2MM1	2	3181746		BB	BB	BB	BB	BB	BB	BB	BB	1	1	1	1	1	1
1757	CH2MM2	2	3972678		BB	BB	BB	BB	BB	BB	BB	BB	1	1	1	1	1	1
149	CH2MM3	2	4196507		AA	AA	AA	AA	AA	AA	AA	AA	1	1	1	1	1	1
1527	CH2MM4	2	4258057		BB	BB	BB	BB	BB	BB	BB	BB	1	1	1	1	1	1
1228	CH2MM5	2	5606042		BB	BB	BB	BB	BB	BB	BB	BB	1	1	1	1	1	1
370	CH2MM6	2	6048930		BB	BB	BB	BB	BB	BB	BB	BB	1	1	1	1	1	1
1908	CH2MM7	2	6694892		AA	AA	AA	AA	AA	AA	AA	AA	1	1	1	1	1	1
371	CH2MM8GM1	2	7326463		AA	AA	AA	AA	AA	AA	AA	AA	1	1	1	1	1	1
150	CH2MM9	2	9780767		BB	BB	BB	BB	BB	BB	BB	BB	1	1	1	1	1	1
1790	CH2MM10	2	10929543		BB	BB	BB	BB	BB	BB	BB	BB	1	1	1	1	1	1
1714	CH2MM11	2	12582823		AA	AA	AA	AA	AA	AA	AA	AA	1	1	1	1	1	1
1838	CH2MM12	2	13669366		BB	BB	BB	BB	BB	BB	BB	BB	1	1	1	1	1	1
139	CH2MM13	2	15618849		BB	BB	BB	BB	BB	BB	BB	BB	1	1	1	1	1	1
1968	CH2MM14	2	17856135		BB	BB	BB	BB	BB	BB	BB	BB	1	1	1	1	1	1
997	CH2MM15BLK4	2	18123731	X	AA	BB	BB	BB	BB	BB	BB	BB	1	1	1	1	1	1
372	CH2MM16GM3	2	19198385		AA	AA	AA	AA	AA	AA	AA	AA	1	1	1	1	1	1
373	CH2MM17	2	19454722		AA	AA	AA	AA	AA	AA	AA	AA	1	1	1	1	1	1
1021	CH2MM18	2	20371950		AA	AA	AA	AA	AA	AA	AA	AA	1	1	1	1	1	1
1711	CH2MM19	2	20814394		AA	AA	AA	AA	AA	AA	AA	AA	1	1	1	1	1	1
1971	CH2MM20	2	23451610		BB	BB	BB	BB	BB	BB	BB	BB	1	1	1	1	1	1
1835	CH2MM21	2	24495175		BB	BB	BB	BB	BB	BB	BB	BB	1	1	1	1	1	1
374	CH2MM22	2	26055543		AA	AA	AA	AA	AA	AA	AA	AA	1	1	1	1	1	1
375	CH2MM23	2	28249160		AA	AA	AA	AA	AA	AA	AA	AA	1	1	1	1	1	1
274	CH2MM24	2	30147850		BB	BB	BB	BB	BB	BB	BB	BB	1	1	1	1	1	1
250	CH2MM25	2	31240017		BB	BB	BB	BB	BB	BB	BB	BB	1	1	1	1	1	1
1724	CH2MM26	2	31660228		BB	BB	BB	BB	BB	BB	BB	BB	1	1	1	1	1	1
67	CH2MM27	2	32981944		BB	BB	BB	BB	BB	BB	BB	BB	1	1	1	1	1	1
151	CH2MM28	2	33954610		AA	AA	AA	AA	AA	AA	AA	AA	1	1	1	1	1	1
1812	CH2MM29	2	35804961		AA	AA	AA	AA	AA	AA	AA	AA	1	1	1	1	1	1
376	CH2MM30	2	38071109		BB	BB	BB	BB	BB	BB	BB	BB	1	1	1	1	1	1
377	CH2MM31GM4	2	39774491		BB	BB	BB	BB	BB	BB	BB	BB	1	1	1	1	1	1
378	CH2MM32	2	41244144		AA	AA	AA	AA	AA	AA	AA	AA	1	1	1	1	1	1
1849	CH2MM33	2	43034728		BB	BB	BB	BB	BB	BB	BB	BB	1	1	1	1	1	1
379	CH2MM34	2	44361765		BB	BB	BB	BB	BB	BB	BB	BB	1	1	1	1	1	1
380	CH2MM35	2	45653218		AA	AA	AA	AA	AA	AA	AA	AA	1	1	1	1	1	1
1815	CH2MM36	2	47124603		AA	AA	AA	AA	AA	AA	AA	AA	1	1	1	1	1	1
1802	CH2MM37	2	48633486		AA	AA	AA	AA	AA	AA	AA	AA	1	1	1	1	1	1
68	CH2MM38	2	50605053		BB	BB	BB	BB	BB	BB	BB	BB	1	1	1	1	1	1
381	CH2MM39	2	50749857		BB	BB	BB	BB	BB	BB	BB	BB	1	1	1	1	1	1
1511	CH2MM40	2	51425686		AA	AA	AA	AA	AA	AA	AA	AA	1	1	1	1	1	1
382	CH2MM41	2	53143600		BB	BB	BB	BB	BB	BB	BB	BB	1	1	1	1	1	1
383	CH2MM42	2	54765962		AA	AA	AA	AA	AA	AA	AA	AA	1	1	1	1	1	1
1669	CH2MM43	2	56335802		AA	AA	AA	AA	AA	AA	AA	AA	1	1	1	1	1	1
1462	CH2MM44	2	56489184		AA	AA	AA	AA	AA	AA	AA	AA	1	1	1	1	1	1
1194	CH2MM45	2	58142938		BB	BB	BB	BB	BB	BB	BB	BB	1	1	1	1	1	1
384	CH2MM46	2	59956872		BB	BB	BB	BB	BB	BB	BB	BB	1	1	1	1	1	1
1597	CH2MM47	2	61738719		AA	AA	AA	AA	AA	AA	AA	AA	1	1	1	1	1	1
69	CH2MM48	2	63143553		AA	AA	AA	AA	AA	AA	AA	AA	1	1	1	1	1	1
385	CH2MM49	2	63511489		BB	BB	BB	BB	BB	BB	BB	BB	1	1	1	1	1	1
1764	CH2MM50	2	65057687		AA	AA	AA	AA	AA	AA	AA	AA	1	1	1	1	1	1
386	CH2MM51	2	66906537		AA	AA	AA	AA	AA	AA	AA	AA	1	1	1	1	1	1
1933	CH2MM52	2	68188374		AA	AA	AA	AA	AA	AA	AA	AA	1	1	1	1	1	1
1918	CH2MM53	2	68927682		AA	AA	AA	AA	AA	AA	AA	AA	1	1	1	1	1	1
1526	CH2MM54	2	70090167		BB	BB	BB	BB	BB	BB	BB	BB	1	1	1	1	1	1
1150	CH2MM55	2	71492729		BB	BB	BB	BB	BB	BB	BB	BB	1	1	1	1	1	1
1668	CH2MM56	2	72190532		BB	BB	BB	BB	BB	BB	BB	BB	1	1	1	1	1	1
387	CH2MM57	2	73189220		AA	AA	AA	AA	AA	AA	AA	AA	1	1	1	1	1	1
70	CH2MM58	2	73370728		AA	AA	AA	AA	AA	AA	AA	AA	1	1	1	1	1	1
388	CH2MM59	2	74776032		BB	BB	BB	BB	BB	BB	BB	BB	1	1	1	1	1	1
152	CH2MM60	2	77549169		BB	BB	BB	BB	BB	BB	BB	BB	1	1	1	1	1	1
71	CH2MM61	2	79237503		BB	BB	BB	BB	BB	BB	BB	BB	1	1	1	1	1	1
1488	CH2MM62	2	80509747		AA	AA	AA	AA	AA	AA	AA	AA	1	1	1	1	1	1
1163	CH2MM63	2	82000176		BB	BB	BB	BB	BB	BB	BB	BB	1	1	1	1	1	1
2030	CH2MM64	2	84666393		AA	AA	AA	AA	AA	AA	AA	AA	1	1	1	1	1	1
2029	CH2MM65	2	84767106		BB	BB	BB	BB	BB	BB	BB	BB	1	1	1	1	1	1

1283	CH2MM66	2	85572783		BB	BB	BB	BB	BB	BB	BB	BB	1	1	1	1	1	1
389	CH2MM67	2	87247149		AA	AA	AA	AA	AA	AA	AA	AA	1	1	1	1	1	1
1974	CH2MM68	2	88899373		BB	BB	BB	BB	BB	BB	BB	BB	1	1	1	1	1	1
390	CH2MM69	2	91539303		AA	AA	AA	AA	AA	AA	AA	AA	1	1	1	1	1	1
979	CH2MM70	2	91632196		AA	AA	AA	AA	AA	AA	AA	AA	1	1	1	1	1	1
391	CH2MM71	2	92720804		AA	AA	AA	AA	AA	AA	AA	AA	1	1	1	1	1	1
1200	CH2MM72	2	94965895		AA	AA	AA	AA	AA	AA	AA	AA	1	1	1	1	1	1
392	CH2MM73	2	95929798		BB	BB	BB	BB	BB	BB	BB	BB	1	1	1	1	1	1
1867	CH2MM74	2	96787868		BB	BB	BB	BB	BB	BB	BB	BB	1	1	1	1	1	1
1698	CH2MM75BLK13	2	97043181	X	BB	AA	AA	AA	AA	AA	AA	AA	1	1	1	1	1	1
72	CH2MM76	2	98216543		BB	BB	BB	BB	BB	BB	BB	BB	1	1	1	1	1	1
1929	CH2MM77	2	99378126		BB	BB	BB	BB	BB	BB	BB	BB	1	1	1	1	1	1
1975	CH2MM78	2	100253963		AA	AA	AA	AA	AA	AA	AA	AA	1	1	1	1	1	1
393	CH2MM79	2	101217024		AA	AA	AA	AA	AA	AA	AA	AA	1	1	1	1	1	1
153	CH2MM80	2	103792940		AA	AA	AA	AA	AA	AA	AA	AA	1	1	1	1	1	1
1106	CH2MM81	2	104083849		AA	AA	AA	AA	AA	AA	AA	AA	1	1	1	1	1	1
394	CH2MM82	2	105190715		AA	AA	AA	AA	AA	AA	AA	AA	1	1	1	1	1	1
395	CH2MM83	2	107356662		AA	AA	AA	AA	AA	AA	AA	AA	1	1	1	1	1	1
396	CH2MM84	2	108396162		AA	AA	AA	AA	AA	AA	AA	AA	1	1	1	1	1	1
397	CH2MM85	2	111095530		AA	AA	AA	AA	AA	AA	AA	AA	1	1	1	1	1	1
64	CH2MM86	2	111981058		BB	BB	BB	BB	BB	BB	BB	BB	1	1	1	1	1	1
1533	CH2MM87	2	114161459		AA	AA	AA	AA	AA	AA	AA	AA	1	1	1	1	1	1
1336	CH2MM88	2	115671423		AA	AA	AA	AA	AA	AA	AA	AA	1	1	1	1	1	1
398	CH2MM89	2	117074373		BB	BB	BB	BB	BB	BB	BB	BB	1	1	1	1	1	1
2038	CH2MM90	2	118948317		AA	AA	AA	AA	AA	AA	AA	AA	1	1	1	1	1	1
1598	CH2MM91	2	120496388		BB	BB	BB	BB	BB	BB	BB	BB	1	1	1	1	1	1
1109	CH2MM92	2	120726441		AA	AA	AA	AA	AA	AA	AA	AA	1	1	1	1	1	1
1496	CH2MM93	2	122067606		AA	AA	AA	AA	AA	AA	AA	AA	1	1	1	1	1	1
1771	CH2MM94	2	123216313		AA	AA	AA	AA	AA	AA	AA	AA	1	1	1	1	1	1
1965	CH2MM95	2	125046796		AA	AA	AA	AA	AA	AA	AA	AA	1	1	1	1	1	1
2001	CH2MM96	2	125050910		AA	AA	AA	AA	AA	AA	AA	AA	1	1	1	1	1	1
399	CH2MM97	2	126890564		AA	AA	AA	AA	AA	AA	AA	AA	1	1	1	1	1	1
154	CH2MM98	2	127549770		BB	BB	BB	BB	BB	BB	BB	BB	1	1	1	1	1	1
1124	CH2MM99	2	129893838		AA	AA	AA	AA	AA	AA	AA	AA	1	1	1	1	1	1
1156	CH2MM100	2	130762932		BB	BB	BB	BB	BB	BB	BB	BB	1	1	1	1	1	1
1599	CH2MM101	2	131742558		BB	BB	BB	BB	BB	BB	BB	BB	1	1	1	1	1	1
400	CH2MM102	2	133610154		BB	BB	BB	BB	BB	BB	BB	BB	1	1	1	1	1	1
65	CH2MM103	2	135876979		BB	BB	BB	BB	BB	BB	BB	BB	1	1	1	1	1	1
1995	CH2MM104	2	136627476		BB	BB	BB	BB	BB	BB	BB	BB	1	1	1	1	1	1
1704	CH2MM105	2	138928693		BB	BB	BB	BB	BB	BB	BB	BB	1	1	1	1	1	1
401	CH2MM106	2	140555557		AA	AA	AA	AA	AA	AA	AA	AA	1	1	1	1	1	1
1012	CH2MM107	2	140793056		AA	AA	AA	AA	AA	AA	AA	AA	1	1	1	1	1	1
402	CH2MM108	2	142149749		BB	BB	BB	BB	BB	BB	BB	BB	1	1	1	1	1	1
964	CH2MM109GM5	2	143604657		BB	BB	BB	BB	BB	BB	BB	BB	1	1	1	1	1	1
963	CH2MM110	2	143777892		BB	BB	BB	BB	BB	BB	BB	BB	1	1	1	1	1	1
403	CH2MM111	2	144125632		BB	BB	BB	BB	BB	BB	BB	BB	1	1	1	1	1	1
1091	CH2MM112	2	144285357		AA	AA	AA	AA	AA	AA	AA	AA	1	1	1	1	1	1
1912	CH2MM113	2	145784318		AA	AA	AA	AA	AA	AA	AA	AA	1	1	1	1	1	1
1322	CH2MM114	2	147404525		BB	BB	BB	BB	BB	BB	BB	BB	1	1	1	1	1	1
1753	CH2MM115	2	147807884		AA	AA	AA	AA	AA	AA	AA	AA	1	1	1	1	1	1
404	CH2MM116	2	149488033		AA	AA	AA	AA	AA	AA	AA	AA	1	1	1	1	1	1
1796	CH2MM117	2	149614906		AA	AA	AA	AA	AA	AA	AA	AA	1	1	1	1	1	1
155	CH2MM118	2	151100533		AA	AA	AA	AA	AA	AA	AA	AA	1	1	1	1	1	1
1799	CH2MM119	2	152642098		BB	BB	BB	BB	BB	BB	BB	BB	1	1	1	1	1	1
1027	CH2MM120	2	153111014	X	AA	BB	BB	BB	BB	BB	BB	BB	1	1	1	1	1	1
1926	CH2MM121	2	154204634		AA	AA	AA	AA	AA	AA	AA	AA	1	1	1	1	1	1
405	CH2MM122	2	156330826		AA	AA	AA	AA	AA	AA	AA	AA	1	1	1	1	1	1
1734	CH2MM123	2	157693960		BB	BB	BB	BB	BB	BB	BB	BB	1	1	1	1	1	1
1306	CH2MM124	2	158068522		AA	AA	AA	AA	AA	AA	AA	AA	1	1	1	1	1	1
406	CH2MM125	2	159733176		BB	BB	BB	BB	BB	BB	BB	BB	1	1	1	1	1	1
1148	CH2MM126	2	161443571		AA	AA	AA	AA	AA	AA	AA	AA	1	1	1	1	1	1
1186	CH2MM127	2	163215888		BB	BB	BB	BB	BB	BB	BB	BB	1	1	1	1	1	1
407	CH2MM128	2	164370875		AA	AA	AA	AA	AA	AA	AA	AA	1	1	1	1	1	1
408	CH2MM129	2	165459397		AA	AA	AA	AA	AA	AA	AA	AA	1	1	1	1	1	1
1429	CH2MM130	2	166073326		AA	AA	AA	AA	AA	AA	AA	AA	1	1	1	1	1	1
409	CH2MM131	2	166374505		BB	BB	BB	BB	BB	BB	BB	BB	1	1	1	1	1	1
66	CH2MM132	2	168586738		AA	AA	AA	AA	AA	AA	AA	AA	1	1	1	1	1	1
1521	CH2MM133	2	169905098		BB	BB	BB	BB	BB	BB	BB	BB	1	1	1	1	1	1
410	CH2MM134	2	170651525		BB	BB	BB	BB	BB	BB	BB	BB	1	1	1	1	1	1
411	CH2MM135	2	171452147		BB	BB	BB	BB	BB	BB	BB	BB	1	1	1	1	1	1
412	CH2MM136	2	174162530		AA	AA	AA	AA	AA	AA	AA	AA	1	1	1	1	1	1
413	CH2MM137	2	174923839		AA	AA	AA	AA	AA	AA	AA	AA	1	1	1	1	1	1
414	CH2MM138	2	178315515		BB	BB	BB	BB	BB	BB	BB	BB	1	1	1	1	1	1
1726	CH2MM139	2	179210967		AA	AA	AA	AA	AA	AA	AA	AA	1	1	1	1	1	1
1233	CH2MM140	2	179814463		AA	AA	AA	AA	AA	AA	AA	AA	1	1	1	1	1	1
415	CH3MM1	3	3793662		BB	BB	BB	BB	BB	BB	BB	BB	1	1	1	1	1	1
416	CH3MM2	3	4378945		AA	AA	AA	AA	AA	AA	AA	AA	1	1	1	1	1	1
417	CH3MM3	3	5656063		AA	AA	AA	AA	AA	AA	AA	AA	1	1	1	1	1	1
418	CH3MM4	3	7703356		BB	BB	BB	BB	BB	BB	BB	BB	1	1	1	1	1	1
419	CH3MM5	3	9744167		BB	BB	BB	BB	BB	BB	BB	BB	1	1	1	1	1	1
1010	CH3MM6	3	9788274		AA	AA	AA	AA	AA	AA	AA	AA	1	1	1	1	1	1
420	CH3MM7	3	11073596		BB	BB	BB	BB	BB	BB	BB	BB	1	1	1	1	1	1
1800	CH3MM8	3	12161848		AA	AA	AA	AA	AA	AA	AA	AA	1	1	1	1	1	1
421	CH3MM9	3	14943619		BB	BB	BB	BB	BB	BB	BB	BB	1	1	1	1	1	1
1114	CH3MM10	3	16122429		AA	AA	AA	AA	AA	AA	AA	AA	1	1	1	1	1	1
156	CH3MM11	3	17691447		AA	AA	AA	AA	AA	AA	AA	AA	1	1	1	1	1	1
263	CH3MM12	3	18932166		AA	AA	AA	AA	AA	AA	AA	AA	1	1	1	1	1	1
1639	CH3MM13	3	19747594		BB	BB	BB	BB	BB	BB	BB	BB	1	1	1	1	1	1
1779	CH3MM14	3	21215374		AA	AA	AA	AA	AA	AA	AA	AA	1	1	1	1	1	1
1219	CH3MM15GM1	3	21221210		BB	BB	BB	BB	BB	BB	BB	BB	1	1	1	1	1	1
1446	CH3MM16	3	26405124		AA	AA	AA	AA	AA	AA	AA	AA	1	1	1	1	1	1
422	CH3MM17	3	27365675		BB	BB	BB	BB	BB	BB	BB	BB	1	1	1	1	1	1
1119	CH3MM18	3	30094761		AA	AA	AA	AA	AA	AA	AA	AA	1	1	1	1	1	1
12	CH3MM19BLK4	3	31168870	X	BB	AA	AA	AA	AA	AA	AA	AA	1	1	1	1	1	1
423	CH3MM20	3	31257023		BB	BB	BB	BB	BB	BB	BB	BB	1	1	1	1	1	1
424	CH3MM21GM2	3	32869483		AA	AA	AA	AA	AA	AA	AA	AA	1	1	1	1	1	1
157	CH3MM22	3	33096061		AA	AA	AA	AA	AA	AA	AA	AA	1	1	1	1	1	1
1600	CH3MM23	3	33860283		AA	AA	AA	AA	AA	AA	AA	AA	1	1	1	1	1	1
1919	CH3MM24	3	33929993		AA	AA	AA	AA	AA	AA	AA	AA	1	1	1	1	1	1
1797	CH3MM25	3	36110851		AA	AA	AA	AA	AA	AA	AA	AA	1	1	1	1	1	1
1823	CH3MM26	3	38689460		BB	BB	BB	BB	BB	BB	BB	BB	1	1	1	1	1	1
1877	CH3MM27	3	39835273		AA	AA	AA	AA	AA	AA	AA	AA	1	1	1	1	1	1
298	CH3MM28	3	41313315		BB	BB	BB	BB	BB	BB	BB	BB	1	1	1	1	1	1
1126	CH3MM29	3	41838640		AA	AA	AA	AA	AA	AA	AA	AA	1	1	1	1	1	1
425	CH3MM30	3	43361057		AA	AA	AA	AA	AA	AA	AA	AA	1	1	1	1	1	1
76	CH3MM31	3	45598106		BB	BB	BB	BB	BB	BB	BB	BB	1	1	1	1	1	1
77	CH3MM32	3	46558020		AA	AA	AA	AA	AA	AA	AA	AA	1	1	1	1	1	1
426	CH3MM33	3	48335184		BB	BB	BB	BB	BB	BB	BB	BB	1	1	1	1	1	1
1676	CH3MM34	3	50461067		AA	AA	AA	AA	AA	AA	AA	AA	1	1	1	1	1	1
1540	CH3MM35	3	50623988		BB	BB	BB	BB	BB	BB	BB	BB	1	1	1	1	1	1
1504	CH3MM36	3	50776152		BB	BB	BB	BB	BB	BB	BB	BB	1	1	1	1	1	1
1791	CH3MM37	3	52675823		AA	AA	AA	AA	AA	AA	AA	AA	1	1	1	1	1	1
1885	CH3MM38	3	53103565		AA	AA	AA	AA	AA	AA	AA	AA	1	1	1	1	1	1
1601	CH3MM39	3	55143939		AA	AA	AA	AA	AA	AA	AA	AA	1	1	1	1	1	1
427	CH3MM40	3	56753970		AA	AA	AA	AA	AA	AA	AA	AA	1	1	1	1	1	1
428	CH3MM41	3	57065840		AA	AA	AA	AA	AA	AA	AA	AA	1	1	1	1	1	1
1854	CH3MM42	3	58612475		AA	AA	AA	AA	AA	AA	AA	AA	1	1	1	1	1	1
1287	CH3MM43	3	59986297		AA	AA	AA	AA	AA	AA	AA	AA	1	1	1	1	1	1
429	CH3MM44	3	60037290		BB	BB	BB	BB	BB	BB	BB	BB	1	1	1	1	1	1
1785	CH3MM45	3	60673679		AA	AA	AA	AA	AA	AA	AA	AA	1	1	1	1	1	1
430	CH3MM46	3	61103349		AA	AA	AA	AA	AA	AA	AA	AA	1	1	1	1	1	1
431	CH3MM47	3	63787516		BB	BB	BB	BB	BB	BB	BB	BB	1	1	1	1	1	1
1756	CH3MM48	3	65222079		BB	BB	BB	BB	BB	BB	BB	BB	1	1	1	1	1	1
158	CH3MM49	3	66994152		BB	BB	BB	BB	BB	BB	BB	BB	1	1	1	1	1	1
432	CH3MM50	3	70089276		AA	AA	AA	AA	AA	AA	AA	AA	1	1	1	1	1	1
1743	CH3MM51	3	70361430		AA	AA	AA	AA	AA	AA	AA	AA	1	1	1	1	1	1
1813	CH3MM52	3	71858927		BB	BB	BB	BB	BB	BB	BB	BB	1	1	1	1	1	1

433	CH3MM53	3	75852874		BB	BB	BB	BB	BB	BB	BB	BB	1	1	1	1	1	1
1695	CH3MM54	3	76280569		AA	AA	AA	AA	AA	AA	AA	AA	1	1	1	1	1	1
434	CH3MM55	3	77646357		AA	AA	AA	AA	AA	AA	AA	AA	1	1	1	1	1	1
435	CH3MM56	3	79085242		AA	AA	AA	AA	AA	AA	AA	AA	1	1	1	1	1	1
1251	CH3MM57	3	79585796		BB	BB	BB	BB	BB	BB	BB	BB	1	1	1	1	1	1
436	CH3MM58	3	80321582		AA	AA	AA	AA	AA	AA	AA	AA	1	1	1	1	1	1
437	CH3MM59	3	81254705		AA	AA	AA	AA	AA	AA	AA	AA	1	1	1	1	1	1
438	CH3MM60	3	81758272		AA	AA	AA	AA	AA	AA	AA	AA	1	1	1	1	1	1
439	CH3MM61	3	83880528		BB	BB	BB	BB	BB	BB	BB	BB	1	1	1	1	1	1
1472	CH3MM62	3	85880745		AA	AA	AA	AA	AA	AA	AA	AA	1	1	1	1	1	1
1647	CH3MM63	3	86394356		AA	AA	AA	AA	AA	AA	AA	AA	1	1	1	1	1	1
1275	CH3MM64	3	86858364		AA	AA	AA	AA	AA	AA	AA	AA	1	1	1	1	1	1
1925	CH3MM65	3	87149248		BB	BB	BB	BB	BB	BB	BB	BB	1	1	1	1	1	1
1536	CH3MM66	3	88133448		AA	AA	AA	AA	AA	AA	AA	AA	1	1	1	1	1	1
2014	CH3MM67	3	89229687		BB	BB	BB	BB	BB	BB	BB	BB	1	1	1	1	1	1
440	CH3MM68	3	89418525		BB	BB	BB	BB	BB	BB	BB	BB	1	1	1	1	1	1
1755	CH3MM69	3	90650953		BB	BB	BB	BB	BB	BB	BB	BB	1	1	1	1	1	1
275	CH3MM70	3	91088111		AA	AA	AA	AA	AA	AA	AA	AA	1	1	1	1	1	1
441	CH3MM71	3	92344335		AA	AA	AA	AA	AA	AA	AA	AA	1	1	1	1	1	1
442	CH3MM72	3	93549679		AA	AA	AA	AA	AA	AA	AA	AA	1	1	1	1	1	1
957	CH3MM73	3	95459009		BB	BB	BB	BB	BB	BB	BB	BB	1	1	1	1	1	1
2002	CH3MM74	3	95491067		BB	BB	BB	BB	BB	BB	BB	BB	1	1	1	1	1	1
443	CH3MM75	3	95678878		AA	AA	AA	AA	AA	AA	AA	AA	1	1	1	1	1	1
1686	CH3MM76	3	95826691		BB	BB	BB	BB	BB	BB	BB	BB	1	1	1	1	1	1
1030	CH3MM77	3	97282711		AA	AA	AA	AA	AA	AA	AA	AA	1	1	1	1	1	1
1517	CH3MM78	3	98954988		AA	AA	AA	AA	AA	AA	AA	AA	1	1	1	1	1	1
1397	CH3MM79GM4	3	99088294		BB	BB	BB	BB	BB	BB	BB	BB	1	1	1	1	1	1
1064	CH3MM80	3	100657788		BB	BB	BB	BB	BB	BB	BB	BB	1	1	1	1	1	1
1537	CH3MM81	3	102619416		AA	AA	AA	AA	AA	AA	AA	AA	1	1	1	1	1	1
1359	CH3MM82	3	103564280		AA	AA	AA	AA	AA	AA	AA	AA	1	1	1	1	1	1
444	CH3MM83	3	105788245		BB	BB	BB	BB	BB	BB	BB	BB	1	1	1	1	1	1
1363	CH3MM84	3	107508882		AA	AA	AA	AA	AA	AA	AA	AA	1	1	1	1	1	1
445	CH3MM85	3	109585764		BB	BB	BB	BB	BB	BB	BB	BB	1	1	1	1	1	1
1214	CH3MM86	3	110649631		AA	AA	AA	AA	AA	AA	AA	AA	1	1	1	1	1	1
1333	CH3MM87	3	112624069		BB	BB	BB	BB	BB	BB	BB	BB	1	1	1	1	1	1
1412	CH3MM88	3	116000138		BB	BB	BB	BB	BB	BB	BB	BB	1	1	1	1	1	1
159	CH3MM89	3	116888086		AA	AA	AA	AA	AA	AA	AA	AA	1	1	1	1	1	1
446	CH3MM90	3	117248777		BB	BB	BB	BB	BB	BB	BB	BB	1	1	1	1	1	1
1404	CH3MM91	3	117343865		BB	BB	BB	BB	BB	BB	BB	BB	1	1	1	1	1	1
1399	CH3MM92	3	118002787		AA	AA	AA	AA	AA	AA	AA	AA	1	1	1	1	1	1
1680	CH3MM93BLK11	3	119108318	X	BB	AA	AA	AA	AA	AB	AB	AB	1	1	1	0,5	0,5	0,5
447	CH3MM94	3	119209671		AA	AA	AA	AA	AA	AA	AA	AA	1	1	1	1	1	1
73	CH3MM95	3	120379605		BB	BB	BB	BB	BB	BB	BB	BB	1	1	1	1	1	1
1112	CH3MM96	3	122002332		AA	AA	AA	AA	AA	AA	AA	AA	1	1	1	1	1	1
947	CH3MM97	3	122261784		AA	AA	AA	AA	AA	AA	AA	AA	1	1	1	1	1	1
946	CH3MM98	3	122266391		BB	BB	BB	BB	BB	BB	BB	BB	1	1	1	1	1	1
448	CH3MM99	3	123230785		BB	BB	BB	BB	BB	BB	BB	BB	1	1	1	1	1	1
255	CH3MM100	3	123767337		BB	BB	BB	BB	BB	BB	BB	BB	1	1	1	1	1	1
449	CH3MM101	3	125504930		AA	AA	AA	AA	AA	AA	AA	AA	1	1	1	1	1	1
1181	CH3MM102	3	126894715		AA	AA	AA	AA	AA	AA	AA	AA	1	1	1	1	1	1
1694	CH3MM103	3	127938746		BB	BB	BB	BB	BB	BB	BB	BB	1	1	1	1	1	1
1175	CH3MM104	3	128820535		BB	BB	BB	BB	BB	BB	BB	BB	1	1	1	1	1	1
450	CH3MM105	3	130952894		AA	AA	AA	AA	AA	AA	AA	AA	1	1	1	1	1	1
451	CH3MM106	3	132189425		BB	BB	BB	BB	BB	BB	BB	BB	1	1	1	1	1	1
74	CH3MM107	3	133070717		AA	AA	AA	AA	AA	AA	AA	AA	1	1	1	1	1	1
452	CH3MM108	3	134785250		BB	BB	BB	BB	BB	BB	BB	BB	1	1	1	1	1	1
1678	CH3MM109	3	134970473		BB	BB	BB	BB	BB	BB	BB	BB	1	1	1	1	1	1
453	CH3MM110	3	135476593		AA	AA	AA	AA	AA	AA	AA	AA	1	1	1	1	1	1
1208	CH3MM111	3	136766271		BB	BB	BB	BB	BB	BB	BB	BB	1	1	1	1	1	1
454	CH3MM112	3	138624013		BB	BB	BB	BB	BB	BB	BB	BB	1	1	1	1	1	1
455	CH3MM113	3	140515828		BB	BB	BB	BB	BB	BB	BB	BB	1	1	1	1	1	1
1057	CH3MM114	3	142598970		BB	BB	BB	BB	BB	BB	BB	BB	1	1	1	1	1	1
1843	CH3MM115	3	143193940		BB	BB	BB	BB	BB	BB	BB	BB	1	1	1	1	1	1
1976	CH3MM116	3	143619317		BB	BB	BB	BB	BB	BB	BB	BB	1	1	1	1	1	1
1653	CH3MM117	3	143871485		AA	AA	AA	AA	AA	AA	AA	AA	1	1	1	1	1	1
1413	CH3MM118	3	146841800		AA	AA	AA	AA	AA	AA	AA	AA	1	1	1	1	1	1
1096	CH3MM119	3	148942421		BB	BB	BB	BB	BB	BB	BB	BB	1	1	1	1	1	1
1045	CH3MM120GM5	3	150449620		BB	BB	BB	BB	BB	BB	BB	BB	1	1	1	1	1	1
456	CH3MM121	3	150745214		BB	BB	BB	BB	BB	BB	BB	BB	1	1	1	1	1	1
1505	CH3MM122	3	151923922		BB	BB	BB	BB	BB	BB	BB	BB	1	1	1	1	1	1
457	CH3MM123	3	152154652		BB	BB	BB	BB	BB	BB	BB	BB	1	1	1	1	1	1
458	CH3MM124	3	154922730		BB	BB	BB	BB	BB	BB	BB	BB	1	1	1	1	1	1
459	CH3MM125	3	155444424		AA	AA	AA	AA	AA	AA	AA	AA	1	1	1	1	1	1
160	CH3MM126	3	157755333		BB	BB	BB	BB	BB	BB	BB	BB	1	1	1	1	1	1
75	CH3MM127	3	159340478		AA	AA	AA	AA	AA	AA	AA	AA	1	1	1	1	1	1
1311	CH4MM1	4	3649824		AA	AA	AA	AA	AA	AA	AA	AA	1	1	1	1	1	1
161	CH4MM2	4	5013576		AA	AA	AA	AA	AA	AA	AA	AA	1	1	1	1	1	1
162	CH4MM3	4	5606396		AA	AA	AA	AA	AA	AA	AA	AA	1	1	1	1	1	1
460	CH4MM4GM1	4	6638138		BB	BB	BB	BB	BB	BB	BB	BB	1	1	1	1	1	1
1120	CH4MM5	4	7127435		BB	BB	BB	BB	BB	BB	BB	BB	1	1	1	1	1	1
461	CH4MM6GM2	4	8730860		AA	AA	AA	AA	AA	AA	AA	AA	1	1	1	1	1	1
2048	CH4MM7	4	10825412		BB	BB	BB	BB	BB	BB	BB	BB	1	1	1	1	1	1
1970	CH4MM8	4	11633492		BB	BB	BB	BB	BB	BB	BB	BB	1	1	1	1	1	1
462	CH4MM9	4	13764168		BB	BB	BB	BB	BB	BB	BB	BB	1	1	1	1	1	1
244	CH4MM10	4	15321444		AA	AA	AA	AA	AA	AA	AA	AA	1	1	1	1	1	1
1837	CH4MM11	4	18045313		BB	BB	BB	BB	BB	BB	BB	BB	1	1	1	1	1	1
78	CH4MM12	4	19508304		AA	AA	AA	AA	AA	AA	AA	AA	1	1	1	1	1	1
463	CH4MM13	4	20665789		BB	BB	BB	BB	BB	BB	BB	BB	1	1	1	1	1	1
163	CH4MM14	4	22305486		AA	AA	AA	AA	AA	AA	AA	AA	1	1	1	1	1	1
1079	CH4MM15	4	22884502		BB	BB	BB	BB	BB	BB	BB	BB	1	1	1	1	1	1
1650	CH4MM16	4	23496604		BB	BB	BB	BB	BB	BB	BB	BB	1	1	1	1	1	1
243	CH4MM17	4	24149712		AA	AA	AA	AA	AA	AA	AA	AA	1	1	1	1	1	1
464	CH4MM18	4	27105003		AA	AA	AA	AA	AA	AA	AA	AA	1	1	1	1	1	1
1356	CH4MM19	4	29487208		AA	AA	AA	AA	AA	AA	AA	AA	1	1	1	1	1	1
79	CH4MM20	4	30653207		AA	AA	AA	AA	AA	AA	AA	AA	1	1	1	1	1	1
1142	CH4MM21	4	31215491		BB	BB	BB	BB	BB	BB	BB	BB	1	1	1	1	1	1
1071	CH4MM22	4	31909897	X	BB	AA	AA	AA	AA	AA	AA	AA	1	1	1	1	1	1
1204	CH4MM23	4	32441103		BB	BB	BB	BB	BB	BB	BB	BB	1	1	1	1	1	1
465	CH4MM24	4	35074381		BB	BB	BB	BB	BB	BB	BB	BB	1	1	1	1	1	1
1470	CH4MM25	4	36221145		BB	BB	BB	BB	BB	BB	BB	BB	1	1	1	1	1	1
1257	CH4MM26	4	38414337		AA	AA	AA	AA	AA	AA	AA	AA	1	1	1	1	1	1
466	CH4MM27	4	39882315		AA	AA	AA	AA	AA	AA	AA	AA	1	1	1	1	1	1
80	CH4MM28	4	40541402		AA	AA	AA	AA	AA	AA	AA	AA	1	1	1	1	1	1
1535	CH4MM29	4	41748426		BB	BB	BB	BB	BB	BB	BB	BB	1	1	1	1	1	1
1330	CH4MM30	4	42429891		BB	BB	BB	BB	BB	BB	BB	BB	1	1	1	1	1	1
468	CH4MM31	4	44111689		AA	AA	AA	AA	AA	AA	AA	AA	1	1	1	1	1	1
469	CH4MM32	4	45420449		AA	AA	AA	AA	AA	AA	AA	AA	1	1	1	1	1	1
467	CH4MM33	4	45887915		BB	BB	BB	BB	BB	BB	BB	BB	1	1	1	1	1	1
1141	CH4MM34	4	47358427		AA	AA	AA	AA	AA	AA	AA	AA	1	1	1	1	1	1
1213	CH4MM35	4	51035410		BB	BB	BB	BB	BB	BB	BB	BB	1	1	1	1	1	1
470	CH4MM36	4	52679398		AA	AA	AA	AA	AA	AA	AA	AA	1	1	1	1	1	1
1098	CH4MM37	4	53909507		AA	AA	AA	AA	AA	AA	AA	AA	1	1	1	1	1	1
1225	CH4MM38	4	53964528		BB	BB	BB	BB	BB	BB	BB	BB	1	1	1	1	1	1
1788	CH4MM39	4	55765284		AA	AA	AA	AA	AA	AA	AA	AA	1	1	1	1	1	1
471	CH4MM40	4	57030578		AA	AA	AA	AA	AA	AA	AA	AA	1	1	1	1	1	1
1428	CH4MM41	4	59103430		BB	BB	BB	BB	BB	BB	BB	BB	1	1	1	1	1	1
1286	CH4MM42	4	61034761		BB	BB	BB	BB	BB	BB	BB	BB	1	1	1	1	1	1
1	CH4MM43GM4	4	61050478		AA	AA	AA	AA	AA	AA	AA	AA	1	1	1	1	1	1
1924	CH4MM44	4	61703299		BB	BB	BB	BB	BB	BB	BB	BB	1	1	1	1	1	1
1803	CH4MM45	4	62013092		AA	AA	AA	AA	AA	AA	AA	AA	1	1	1	1	1	1
1881	CH4MM46	4	62225528		BB	BB	BB	BB	BB	BB	BB	BB	1	1	1	1	1	1
1066	CH4MM47BLK5	4	62455748	X	BB	AA	AA	AA	AA	AA	AA	AA	1	1	1	1	1	1
472	CH4MM48	4	64811196		AA	AA	AA	AA	AA	AA	AA	AA	1	1	1	1	1	1
930	CH4MM49	4	66889112		BB	BB	BB	BB	BB	BB	BB	BB	1	1	1	1	1	1
473	CH4MM50	4	67604678		AA	AA	AA	AA	AA	AA	AA	AA	1	1	1	1	1	1
1409	CH4MM51	4	68727690		AA	AA	AA	AA	AA	AA	AA	AA	1	1	1	1	1	1
474	CH4MM52	4	69553312		AA	AA	AA	AA	AA	AA	AA	AA	1	1	1	1	1	1

1763	CH4MM53	4	70467819		AA	AA	AA	AA	AA	AA	AA	AA	1	1	1	1	1	1
475	CH4MM54	4	71868643		AA	AA	AA	AA	AA	AA	AA	AA	1	1	1	1	1	1
476	CH4MM55	4	74403260		BB	BB	BB	BB	BB	BB	BB	BB	1	1	1	1	1	1
1400	CH4MM56	4	75144974		AA	AA	AA	AA	AA	AA	AA	AA	1	1	1	1	1	1
81	CH4MM57	4	77952063		AA	AA	AA	AA	AA	AA	AA	AA	1	1	1	1	1	1
1394	CH4MM58	4	79579536		AA	AA	AA	AA	AA	AA	AA	AA	1	1	1	1	1	1
1538	CH4MM59	4	79969982		BB	BB	BB	BB	BB	BB	BB	BB	1	1	1	1	1	1
286	CH4MM60GM5-BM	4	80481305		BB	BB	BB	BB	BB	BB	BB	BB	1	1	1	1	1	1
1216	CH4MM61	4	81040027		BB	BB	BB	BB	BB	BB	BB	BB	1	1	1	1	1	1
1337	CH4MM62	4	81462943		AA	AA	AA	AA	AA	AA	AA	AA	1	1	1	1	1	1
477	CH4MM63	4	82384465		AA	AA	AA	AA	AA	AA	AA	AA	1	1	1	1	1	1
1421	CH4MM64	4	84506191		AA	AA	AA	AA	AA	AA	AA	AA	1	1	1	1	1	1
478	CH4MM65	4	87260441		AA	AA	AA	AA	AA	AA	AA	AA	1	1	1	1	1	1
1752	CH4MM66	4	88269130		BB	BB	BB	BB	BB	BB	BB	BB	1	1	1	1	1	1
479	CH4MM67	4	89450763		BB	BB	BB	BB	BB	BB	BB	BB	1	1	1	1	1	1
1820	CH4MM68	4	91308682		AA	AA	AA	AA	AA	AA	AA	AA	1	1	1	1	1	1
1111	CH4MM69	4	94278186		BB	BB	BB	BB	BB	BB	BB	BB	1	1	1	1	1	1
480	CH4MM70	4	95674511		AA	AA	AA	AA	AA	AA	AA	AA	1	1	1	1	1	1
1373	CH4MM71	4	97279502		AA	AA	AA	AA	AA	AA	AA	AA	1	1	1	1	1	1
481	CH4MM72	4	97650355		AA	AA	AA	AA	AA	AA	AA	AA	1	1	1	1	1	1
482	CH4MM73	4	99775886		BB	BB	BB	BB	BB	BB	BB	BB	1	1	1	1	1	1
1864	CH4MM74	4	101395000		BB	BB	BB	BB	BB	BB	BB	BB	1	1	1	1	1	1
282	CH4MM75GM6-BM	4	101553258		BB	BB	BB	BB	BB	BB	BB	BB	1	1	1	1	1	1
1498	CH4MM76	4	101657612		AA	AA	AA	AA	AA	AA	AA	AA	1	1	1	1	1	1
483	CH4MM77	4	102307704		BB	BB	BB	BB	BB	BB	BB	BB	1	1	1	1	1	1
1876	CH4MM78	4	104323754		AA	AA	AA	AA	AA	AA	AA	AA	1	1	1	1	1	1
1770	CH4MM79	4	107056497		BB	BB	BB	BB	BB	BB	BB	BB	1	1	1	1	1	1
1414	CH4MM80	4	108380717		AA	AA	AA	AA	AA	AA	AA	AA	1	1	1	1	1	1
1405	CH4MM81	4	109968331		BB	BB	BB	BB	BB	BB	BB	BB	1	1	1	1	1	1
1931	CH4MM82	4	114381685		BB	BB	BB	BB	BB	BB	BB	BB	1	1	1	1	1	1
1294	CH4MM83	4	116737689		BB	BB	BB	BB	BB	BB	BB	BB	1	1	1	1	1	1
1915	CH4MM84	4	117526748		AA	AA	AA	AA	AA	AA	AA	AA	1	1	1	1	1	1
1278	CH4MM85	4	118066883		AA	AA	AA	AA	AA	AA	AA	AA	1	1	1	1	1	1
1205	CH4MM86	4	119404209		BB	BB	BB	BB	BB	BB	BB	BB	1	1	1	1	1	1
484	CH4MM87	4	121322200		AA	AA	AA	AA	AA	AA	AA	AA	1	1	1	1	1	1
485	CH4MM88	4	122566268		BB	BB	BB	BB	BB	BB	BB	BB	1	1	1	1	1	1
1442	CH4MM89	4	122578236		BB	BB	BB	BB	BB	BB	BB	BB	1	1	1	1	1	1
486	CH4MM90	4	125082422		BB	BB	BB	BB	BB	BB	BB	BB	1	1	1	1	1	1
1022	CH4MM91BLK11	4	125168700	X	AA	BB	BB	BB	BB	BB	BB	BB	1	1	1	1	1	1
164	CH4MM92	4	126400415		BB	BB	BB	BB	BB	BB	BB	BB	1	1	1	1	1	1
1901	CH4MM93	4	128083691		AA	AA	AA	AA	AA	AA	AA	AA	1	1	1	1	1	1
487	CH4MM94	4	128323848		BB	BB	BB	BB	BB	BB	BB	BB	1	1	1	1	1	1
2049	CH4MM95	4	131705391		AA	AA	AA	AA	AA	AA	AA	AA	1	1	1	1	1	1
1144	CH4MM96	4	134187547		AA	AA	AA	AA	AA	AA	AA	AA	1	1	1	1	1	1
488	CH4MM97	4	135132664		BB	BB	BB	BB	BB	BB	BB	BB	1	1	1	1	1	1
1944	CH4MM98	4	138224144		AA	AA	AA	AA	AA	AA	AA	AA	1	1	1	1	1	1
1059	CH4MM99	4	138743913		AA	AA	AA	AA	AA	AA	AA	AA	1	1	1	1	1	1
1274	CH4MM100	4	140026440		AA	AA	AA	AA	AA	AA	AA	AA	1	1	1	1	1	1
1477	CH4MM101	4	140961583		AA	AA	AA	AA	AA	AA	AA	AA	1	1	1	1	1	1
1031	CH4MM102	4	141638718		AA	AA	AA	AA	AA	AA	AA	AA	1	1	1	1	1	1
1776	CH4MM103	4	145785696		AA	AA	AA	AA	AA	AA	AA	AA	1	1	1	1	1	1
1817	CH4MM104	4	149902058		AA	AA	AA	AA	AA	AA	AA	AA	1	1	1	1	1	1
491	CH4MM105	4	152316000		BB	BB	BB	BB	BB	BB	BB	BB	1	1	1	1	1	1
489	CH4MM106	4	153104114		AA	AA	AA	AA	AA	AA	AA	AA	1	1	1	1	1	1
490	CH4MM107	4	153388518		AA	AA	AA	AA	AA	AA	AA	AA	1	1	1	1	1	1
1688	CH4MM108	4	153505697		BB	BB	BB	BB	BB	BB	BB	BB	1	1	1	1	1	1
1829	CH4MM109	4	154069720		AA	AA	AA	AA	AA	AA	AA	AA	1	1	1	1	1	1
265	CH5MM1	5	3061523		AA	AA	AA	AA	AA	AA	AA	AA	1	1	1	1	1	1
492	CH5MM2	5	3601413		AA	AA	AA	AA	AA	AA	AA	AA	1	1	1	1	1	1
493	CH5MM3GM1	5	3974781		AA	AA	AA	AA	AA	AA	AA	AA	1	1	1	1	1	1
494	CH5MM4	5	4310159		BB	BB	BB	BB	BB	BB	BB	BB	1	1	1	1	1	1
1149	CH5MM5	5	4474737		AA	AA	AA	AA	AA	AA	AA	AA	1	1	1	1	1	1
85	CH5MM6	5	4604586		AA	AA	AA	AA	AA	AA	AA	AA	1	1	1	1	1	1
1969	CH5MM7	5	5438798		BB	BB	BB	BB	BB	BB	BB	BB	1	1	1	1	1	1
1794	CH5MM8GM2	5	7469880		BB	BB	BB	BB	BB	BB	BB	BB	1	1	1	1	1	1
1857	CH5MM9	5	7652759		AA	AA	AA	AA	AA	AA	AA	AA	1	1	1	1	1	1
495	CH5MM10	5	9748059		AA	AA	AA	AA	AA	AA	AA	AA	1	1	1	1	1	1
1440	CH5MM11	5	11417181		BB	BB	BB	BB	BB	BB	BB	BB	1	1	1	1	1	1
1271	CH5MM12	5	13389454		BB	BB	BB	BB	BB	BB	BB	BB	1	1	1	1	1	1
1895	CH5MM13	5	13875606		AA	AA	AA	AA	AA	AA	AA	AA	1	1	1	1	1	1
1290	CH5MM14	5	16224015		AA	AA	AA	AA	AA	AA	AA	AA	1	1	1	1	1	1
1778	CH5MM15	5	17667733		AA	AA	AA	AA	AA	AA	AA	AA	1	1	1	1	1	1
1629	CH5MM16	5	19122639		AA	AA	AA	AA	AA	AA	AA	AA	1	1	1	1	1	1
496	CH5MM17	5	19993162		AA	AA	AA	AA	AA	AA	AA	AA	1	1	1	1	1	1
497	CH5MM18	5	21398479		AA	AA	AA	AA	AA	AA	AA	AA	1	1	1	1	1	1
1894	CH5MM19	5	23273638		AA	AA	AA	AA	AA	AA	AA	AA	1	1	1	1	1	1
1382	CH5MM20	5	23524916		AA	AA	AA	AA	AA	AA	AA	AA	1	1	1	1	1	1
83	CH5MM21	5	24211033		BB	BB	BB	BB	BB	BB	BB	BB	1	1	1	1	1	1
498	CH5MM22	5	25501830		AA	AA	AA	AA	AA	AA	AA	AA	1	1	1	1	1	1
1320	CH5MM23	5	26095730		AA	AA	AA	AA	AA	AA	AA	AA	1	1	1	1	1	1
1705	CH5MM24	5	26815473		AA	AA	AA	AA	AA	AA	AA	AA	1	1	1	1	1	1
1237	CH5MM25	5	27162034		BB	BB	BB	BB	BB	BB	BB	BB	1	1	1	1	1	1
960	CH5MM26	5	27460781		BB	BB	BB	BB	BB	BB	BB	BB	1	1	1	1	1	1
951	CH5MM27	5	27720997		BB	BB	BB	BB	BB	BB	BB	BB	1	1	1	1	1	1
961	CH5MM28GM3	5	27781564		BB	BB	BB	BB	BB	BB	BB	BB	1	1	1	1	1	1
319	CH5MM29	5	29486094		AA	AA	AA	AA	AA	AA	AA	AA	1	1	1	1	1	1
1350	CH5MM30	5	31071103		AA	AA	AA	AA	AA	AA	AA	AA	1	1	1	1	1	1
1104	CH5MM31	5	31765822		AA	AA	AA	AA	AA	AA	AA	AA	1	1	1	1	1	1
299	CH5MM32	5	33353887		AA	AA	AA	AA	AA	AA	AA	AA	1	1	1	1	1	1
1198	CH5MM33	5	33963604		AA	AA	AA	AA	AA	AA	AA	AA	1	1	1	1	1	1
499	CH5MM34	5	35473596		BB	BB	BB	BB	BB	BB	BB	BB	1	1	1	1	1	1
1888	CH5MM35	5	35567326		BB	BB	BB	BB	BB	BB	BB	BB	1	1	1	1	1	1
1663	CH5MM36	5	38260635		AA	AA	AA	AA	AA	AA	AA	AA	1	1	1	1	1	1
1805	CH5MM37	5	38327673		AA	AA	AA	AA	AA	AA	AA	AA	1	1	1	1	1	1
500	CH5MM38	5	39608047		AA	AA	AA	AA	AA	AA	AA	AA	1	1	1	1	1	1
1760	CH5MM39	5	42914749		BB	BB	BB	BB	BB	BB	BB	BB	1	1	1	1	1	1
501	CH5MM40	5	43586480		AA	AA	BB	AB	BB	BB	BB	BB	0	0,5	0	0	0	0
502	CH5MM41	5	43971357		BB	BB	BB	BB	BB	BB	BB	BB	1	1	1	1	1	1
1113	CH5MM42	5	44700900		BB	BB	BB	BB	BB	BB	BB	BB	1	1	1	1	1	1
84	CH5MM43	5	45872918		BB	BB	BB	BB	BB	BB	BB	BB	1	1	1	1	1	1
1733	CH5MM44	5	47975043		AA	AA	AA	AA	AA	AA	AA	AA	1	1	1	1	1	1
1437	CH5MM45	5	49310150		AA	AA	AA	AA	AA	AA	AA	AA	1	1	1	1	1	1
1648	CH5MM46	5	50389015		AA	AA	AA	AA	AA	AA	AA	AA	1	1	1	1	1	1
1121	CH5MM47	5	51028268		BB	BB	BB	BB	BB	BB	BB	BB	1	1	1	1	1	1
1146	CH5MM48	5	52283097		AA	AA	AA	AA	AA	AA	AA	AA	1	1	1	1	1	1
249	CH5MM49	5	53853311		BB	BB	BB	BB	BB	BB	BB	BB	1	1	1	1	1	1
86	CH5MM50	5	56167948		AA	AA	AA	AA	AA	AA	AA	AA	1	1	1	1	1	1
1075	CH5MM51BLK5	5	58076839	X	AA	BB	BB	BB	BB	BB	BB	BB	1	1	1	1	1	1
1058	CH5MM52GM4	5	58891807		BB	BB	BB	BB	BB	BB	BB	BB	1	1	1	1	1	1
1898	CH5MM53	5	60155561		AA	AA	AA	AA	AA	AA	AA	AA	1	1	1	1	1	1
1725	CH5MM54	5	61437749		AA	AA	AA	AA	AA	AA	AA	AA	1	1	1	1	1	1
251	CH5MM55	5	62818443		AA	AA	AA	AA	AA	AA	AA	AA	1	1	1	1	1	1
1603	CH5MM56	5	65265272		BB	BB	BB	BB	BB	BB	BB	BB	1	1	1	1	1	1
1602	CH5MM57	5	65265514		AA	AA	AA	AA	AA	AA	AA	AA	1	1	1	1	1	1
503	CH5MM58	5	65750331		BB	BB	BB	BB	BB	BB	BB	BB	1	1	1	1	1	1
165	CH5MM59	5	65952319		BB	BB	BB	BB	BB	BB	BB	BB	1	1	1	1	1	1
1305	CH5MM60	5	69049778		BB	BB	BB	BB	BB	BB	BB	BB	1	1	1	1	1	1
1887	CH5MM61	5	71436027		BB	BB	BB	BB	BB	BB	BB	BB	1	1	1	1	1	1
1707	CH5MM62	5	73093296		AA	AA	AA	AA	AA	AA	AA	AA	1	1	1	1	1	1
166	CH5MM63	5	73573998		BB	BB	BB	BB	BB	BB	BB	BB	1	1	1	1	1	1
245	CH5MM64	5	75065363		BB	BB	BB	BB	BB	BB	BB	BB	1	1	1	1	1	1
504	CH5MM65	5	76181811		BB	BB	BB	BB	BB	BB	BB	BB	1	1	1	1	1	1
87	CH5MM66	5	79059370		BB	BB	BB	BB	BB	BB	BB	BB	1	1	1	1	1	1
505	CH5MM67	5	79902641		BB	BB	BB	BB	BB	BB	BB	BB	1	1	1	1	1	1
1485	CH5MM68	5	81282125		AA	AA	AA	AA	AA	AA	AA	AA	1	1	1	1	1	1
1166	CH5MM69	5	81819770		AA	AA	AA	AA	AA	AA	AA	AA	1	1	1	1	1	1
300	CH5MM70	5	85884364		AA	AA	AA	AA	AA	AA	AA	AA	1	1	1	1	1	1

88	CH5MM71	5	87173557		AA	AA	AA	AA	AA	AA	AA	AA	1	1	1	1	1	1
167	CH5MM72	5	88364567		AA	AA	AA	AA	AA	AA	AA	AA	1	1	1	1	1	1
1197	CH5MM73	5	88498569		BB	BB	BB	BB	BB	BB	BB	BB	1	1	1	1	1	1
506	CH5MM74	5	89674255		AA	AA	AA	AA	AA	AA	AA	AA	1	1	1	1	1	1
1777	CH5MM75	5	90936845		AA	AA	AA	AA	AA	AA	AA	AA	1	1	1	1	1	1
1073	CH5MM76	5	92081078	X	BB	AA	AA	AA	AA	AA	AA	AA	1	1	1	1	1	1
507	CH5MM77	5	93496397		BB	BB	BB	BB	BB	BB	BB	BB	1	1	1	1	1	1
89	CH5MM78	5	93652588		BB	BB	BB	BB	BB	BB	BB	BB	1	1	1	1	1	1
90	CH5MM79	5	93945748		BB	BB	BB	BB	BB	BB	BB	BB	1	1	1	1	1	1
1123	CH5MM80	5	95154383		BB	BB	BB	BB	BB	BB	BB	BB	1	1	1	1	1	1
1374	CH5MM81	5	96618852		BB	BB	BB	BB	BB	BB	BB	BB	1	1	1	1	1	1
508	CH5MM82	5	101291108		BB	BB	BB	BB	BB	BB	BB	BB	1	1	1	1	1	1
1518	CH5MM83	5	105832285		AA	AA	AA	AA	AA	AA	AA	AA	1	1	1	1	1	1
509	CH5MM84	5	106102700		AA	AA	AA	AA	AA	AA	AA	AA	1	1	1	1	1	1
314	CH5MM85	5	107435156		BB	BB	BB	BB	BB	BB	BB	BB	1	1	1	1	1	1
291	CH5MM86GM5-BM	5	108661000		BB	BB	BB	BB	BB	BB	BB	BB	1	1	1	1	1	1
2051	CH5MM87	5	108678291		BB	BB	BB	BB	BB	BB	BB	BB	1	1	1	1	1	1
168	CH5MM88	5	109421273		AA	AA	AA	AA	AA	AA	AA	AA	1	1	1	1	1	1
510	CH5MM89	5	111603432		AA	AA	AA	AA	AA	AA	AA	AA	1	1	1	1	1	1
169	CH5MM90	5	113532900		AA	AA	AA	AA	AA	AA	AA	AA	1	1	1	1	1	1
1138	CH5MM91	5	114196024		AA	AA	AA	AA	AA	AA	AA	AA	1	1	1	1	1	1
1130	CH5MM92BLK10	5	114905705	X	BB	AA	AA	AA	AA	AA	AA	AA	1	1	1	1	1	1
511	CH5MM93	5	115405250		BB	BB	BB	BB	BB	BB	BB	BB	1	1	1	1	1	1
82	CH5MM94	5	117374791		BB	BB	BB	BB	BB	BB	BB	BB	1	1	1	1	1	1
1988	CH5MM95	5	119078055		AA	AA	AA	AA	AA	AA	AA	AA	1	1	1	1	1	1
1641	CH5MM96	5	119114567		BB	BB	BB	BB	BB	BB	BB	BB	1	1	1	1	1	1
512	CH5MM97	5	119747337		AA	AA	AA	AA	AA	AA	AA	AA	1	1	1	1	1	1
513	CH5MM98	5	121338564		BB	BB	BB	BB	BB	BB	BB	BB	1	1	1	1	1	1
1182	CH5MM99	5	122343598		AA	AA	AA	AA	AA	AA	AA	AA	1	1	1	1	1	1
170	CH5MM100	5	123654667		BB	BB	BB	BB	BB	BB	BB	BB	1	1	1	1	1	1
171	CH5MM101	5	124102640		BB	BB	BB	BB	BB	BB	BB	BB	1	1	1	1	1	1
514	CH5MM102	5	125334324		AA	AA	AA	AA	AA	AA	AA	AA	1	1	1	1	1	1
515	CH5MM103	5	126818001		AA	AA	AA	AA	AA	AA	AA	AA	1	1	1	1	1	1
172	CH5MM104	5	127936509		BB	BB	BB	BB	BB	BB	BB	BB	1	1	1	1	1	1
1279	CH5MM105GM6	5	129120319		AA	AA	AA	AA	AA	AA	AA	AA	1	1	1	1	1	1
516	CH5MM106	5	131766121		AA	AA	AA	AA	AA	AA	AA	AA	1	1	1	1	1	1
517	CH5MM107	5	133978719		BB	BB	BB	BB	BB	BB	BB	BB	1	1	1	1	1	1
935	CH5MM108	5	134295595		AA	AA	AA	AA	AA	AA	AA	AA	1	1	1	1	1	1
936	CH5MM109	5	134403691		AA	AA	AA	AA	AA	AA	AA	AA	1	1	1	1	1	1
925	CH5MM110	5	134613310		AA	AA	AA	AA	AA	AA	AA	AA	1	1	1	1	1	1
926	CH5MM111	5	134659511		BB	BB	BB	BB	BB	BB	BB	BB	1	1	1	1	1	1
937	CH5MM112	5	134777746		AA	AA	AA	AA	AA	AA	AA	AA	1	1	1	1	1	1
927	CH5MM113	5	134958116		BB	BB	BB	BB	BB	BB	BB	BB	1	1	1	1	1	1
928	CH5MM114	5	135108981		BB	BB	BB	BB	BB	BB	BB	BB	1	1	1	1	1	1
938	CH5MM115	5	135130636		AA	AA	AA	AA	AA	AA	AA	AA	1	1	1	1	1	1
929	CH5MM116	5	135298261		BB	BB	BB	BB	BB	BB	BB	BB	1	1	1	1	1	1
939	CH5MM117	5	135641949		AA	AA	AA	AA	AA	AA	AA	AA	1	1	1	1	1	1
1604	CH5MM118	5	135836567		AA	AA	AA	AA	AA	AA	AA	AA	1	1	1	1	1	1
518	CH5MM119	5	136901638		BB	BB	BB	BB	BB	BB	BB	BB	1	1	1	1	1	1
1009	CH5MM120	5	137132978		AA	AA	AA	AA	AA	AA	AA	AA	1	1	1	1	1	1
1852	CH5MM121	5	137173297		AA	AA	AA	AA	AA	AA	AA	AA	1	1	1	1	1	1
1093	CH5MM122	5	139206611		BB	BB	BB	BB	BB	BB	BB	BB	1	1	1	1	1	1
519	CH5MM123	5	141567331		AA	AA	AA	AA	AA	AA	AA	AA	1	1	1	1	1	1
2052	CH5MM124	5	142025032		AA	AA	AA	AA	AA	AA	AA	AA	1	1	1	1	1	1
520	CH5MM125	5	142689128		AA	AA	AA	AA	AA	AA	AA	AA	1	1	1	1	1	1
1465	CH5MM126	5	147051702		BB	BB	BB	BB	BB	BB	BB	BB	1	1	1	1	1	1
943	CH5MM127	5	149987068		AA	AA	AA	AA	AA	AA	AA	AA	1	1	1	1	1	1
933	CH5MM128	5	150296116		BB	BB	BB	BB	BB	BB	BB	BB	1	1	1	1	1	1
942	CH5MM129	5	150297141		BB	BB	BB	BB	BB	BB	BB	BB	1	1	1	1	1	1
941	CH5MM130	5	150313944		AA	AA	AA	AA	AA	AA	AA	AA	1	1	1	1	1	1
1128	CH6MM1	6	3167392		BB	BB	BB	BB	BB	BB	BB	BB	1	1	1	1	1	1
521	CH6MM2	6	3547547		BB	BB	BB	BB	BB	BB	BB	BB	1	1	1	1	1	1
522	CH6MM3GM1	6	4162327		AA	AA	AA	AA	AA	AA	AA	AA	1	1	1	1	1	1
1715	CH6MM4	6	4362632		AA	AA	AA	AA	AA	AA	AA	AA	1	1	1	1	1	1
1684	CH6MM5	6	5897994		AA	AA	AA	AA	AA	AA	AA	AA	1	1	1	1	1	1
1344	CH6MM6	6	6161470		AA	AA	AA	AA	AA	AA	AA	AA	1	1	1	1	1	1
523	CH6MM7GM2	6	7747436		AA	AA	AA	AA	AA	AA	AA	AA	1	1	1	1	1	1
524	CH6MM8GM3	6	8907544		AA	AA	AA	AA	AA	AA	AA	AA	1	1	1	1	1	1
525	CH6MM9	6	9890882		AA	AA	AA	AA	AA	AA	AA	AA	1	1	1	1	1	1
526	CH6MM10	6	11055603		AA	AA	AA	AA	AA	AA	AA	AA	1	1	1	1	1	1
1691	CH6MM11	6	13947306		AA	AA	AA	AA	AA	AA	AA	AA	1	1	1	1	1	1
1231	CH6MM12	6	14045168		AA	AA	AA	AA	AA	AA	AA	AA	1	1	1	1	1	1
527	CH6MM13	6	15816033		BB	BB	BB	BB	BB	BB	BB	BB	1	1	1	1	1	1
1086	CH6MM14	6	17715100		BB	BB	BB	BB	BB	BB	BB	BB	1	1	1	1	1	1
528	CH6MM15	6	18376687		AA	AA	AA	AA	AA	AA	AA	AA	1	1	1	1	1	1
529	CH6MM16	6	21755667		BB	BB	BB	BB	BB	BB	BB	BB	1	1	1	1	1	1
1260	CH6MM17	6	24248321		BB	BB	BB	BB	BB	BB	BB	BB	1	1	1	1	1	1
1185	CH6MM18	6	25646449		BB	BB	BB	BB	BB	BB	BB	BB	1	1	1	1	1	1
530	CH6MM19	6	27804113		AA	AA	AA	AA	AA	AA	AA	AA	1	1	1	1	1	1
13	CH6MM20BLK2	6	28665963	X	AA	BB	BB	BB	BB	BB	BB	BB	1	1	1	1	1	1
281	CH6MM21GM4-BM	6	29020950		BB	BB	BB	BB	BB	BB	BB	BB	1	1	1	1	1	1
173	CH6MM22	6	29269458		BB	BB	BB	BB	BB	BB	BB	BB	1	1	1	1	1	1
531	CH6MM23	6	32730020		AA	AA	AA	AA	AA	AA	AA	AA	1	1	1	1	1	1
532	CH6MM24	6	34409100		BB	BB	BB	BB	BB	BB	BB	BB	1	1	1	1	1	1
1369	CH6MM25	6	36040358		BB	BB	BB	BB	BB	BB	BB	BB	1	1	1	1	1	1
1884	CH6MM26	6	37461200		AA	AA	AA	AA	AA	AA	AA	AA	1	1	1	1	1	1
533	CH6MM27	6	37618507		BB	BB	BB	BB	BB	BB	BB	BB	1	1	1	1	1	1
534	CH6MM28	6	38474383		BB	BB	BB	BB	BB	BB	BB	BB	1	1	1	1	1	1
174	CH6MM29	6	40733942		BB	BB	BB	BB	BB	BB	BB	BB	1	1	1	1	1	1
535	CH6MM30	6	43778689		AA	AA	AA	AA	AA	AA	AA	AA	1	1	1	1	1	1
536	CH6MM31	6	44147269		BB	BB	BB	BB	BB	BB	BB	BB	1	1	1	1	1	1
537	CH6MM32	6	44523705		BB	BB	BB	BB	BB	BB	BB	BB	1	1	1	1	1	1
1783	CH6MM33	6	45234635		AA	AA	AA	AA	AA	AA	AA	AA	1	1	1	1	1	1
538	CH6MM34	6	45420260		AA	AA	AA	AA	AA	AA	AA	AA	1	1	1	1	1	1
539	CH6MM35	6	48964507		BB	BB	BB	BB	BB	BB	BB	BB	1	1	1	1	1	1
1693	CH6MM36	6	49606212		AA	AA	AA	AA	AA	AA	AA	AA	1	1	1	1	1	1
1539	CH6MM37	6	49813889		AA	AA	AA	AA	AA	AA	AA	AA	1	1	1	1	1	1
540	CH6MM38	6	51145321		AA	AA	AA	AA	AA	AA	AA	AA	1	1	1	1	1	1
541	CH6MM39	6	53702681		BB	BB	BB	BB	BB	BB	BB	BB	1	1	1	1	1	1
1900	CH6MM40	6	56316458		BB	BB	BB	BB	BB	BB	BB	BB	1	1	1	1	1	1
1028	CH6MM41GM5	6	56497874		AA	AA	AA	AA	AA	AA	AA	AA	1	1	1	1	1	1
92	CH6MM42	6	57082524		BB	BB	BB	BB	BB	BB	BB	BB	1	1	1	1	1	1
542	CH6MM43	6	59609561		BB	BB	BB	BB	BB	BB	BB	BB	1	1	1	1	1	1
1930	CH6MM44	6	63916748		AA	AA	AA	AA	AA	AA	AA	AA	1	1	1	1	1	1
543	CH6MM45	6	65211036		AA	AA	AA	AA	AA	AA	AA	AA	1	1	1	1	1	1
1896	CH6MM46	6	67548202		AA	AA	AA	AA	AA	AA	AA	AA	1	1	1	1	1	1
1738	CH6MM47	6	69590221		BB	BB	BB	BB	BB	BB	BB	BB	1	1	1	1	1	1
1523	CH6MM48	6	71134510		AA	AA	AA	AA	AA	AA	AA	AA	1	1	1	1	1	1
280	CH6MM49	6	71532444		AA	AA	AA	AA	AA	AA	AA	AA	1	1	1	1	1	1
544	CH6MM50	6	73538842		BB	BB	BB	BB	BB	BB	BB	BB	1	1	1	1	1	1
1187	CH6MM51	6	75631345		BB	BB	BB	BB	BB	BB	BB	BB	1	1	1	1	1	1
1339	CH6MM52	6	76679118		AA	AA	AA	AA	AA	AA	AA	AA	1	1	1	1	1	1
1376	CH6MM53	6	77343585		BB	BB	BB	BB	BB	BB	BB	BB	1	1	1	1	1	1
545	CH6MM54	6	78477103		BB	BB	BB	BB	BB	BB	BB	BB	1	1	1	1	1	1
1927	CH6MM55	6	80032286		AA	AA	AA	AA	AA	AA	AA	AA	1	1	1	1	1	1
1660	CH6MM56BLK10	6	80305689	X	BB	AA	AA	AA	AA	AA	AA	AA	1	1	1	1	1	1
1719	CH6MM57	6	81674296		AA	AA	AA	AA	AA	AA	AA	AA	1	1	1	1	1	1
93	CH6MM58	6	83434907		AA	AA	AA	AA	AA	AA	AA	AA	1	1	1	1	1	1
301	CH6MM59	6	85136988		AA	AA	AA	AB	AB	AA	AB	AB	1	0,5	0,5	1	0,5	0,5
94	CH6MM60	6	86289708		BB	BB	BB	AB	AB	BB	AB	AB	1	0,5	0,5	1	0,5	0,5
1816	CH6MM61	6	87214811		AA	AA	AA	AB	AB	AA	AB	AB	1	0,5	0,5	1	0,5	0,5
546	CH6MM62	6	90003224		AA	AA	AA	AA	AA	AA	AA	AA	1	1	1	1	1	1
1632	CH6MM63	6	91775036		BB	BB	BB	BB	BB	BB	BB	BB	1	1	1	1	1	1
996	CH6MM64	6	92163048		AA	AA	AA	AA	AA	AA	AA	AA	1	1	1	1	1	1
547	CH6MM65	6	92361613		AA	AA	AA	AA	AA	AA	AA	AA	1	1	1	1	1	1
1833	CH6MM66	6	93892091		BB	BB	BB	BB	BB	BB	BB	BB	1	1	1	1	1	1
1784	CH6MM67	6	94413050		BB	BB	BB	AB	AB	BB	AB	AB	1	0,5	0,5	1	0,5	0,5

548	CH6MM68	6	95758062		AA	AA	AA	AA	AA	AA	AA	AA	1	1	1	1	1	1
1893	CH6MM69	6	97121415		BB	BB	BB	BB	BB	BB	BB	BB	1	1	1	1	1	1
549	CH6MM70	6	98304818		AA	AA	AA	AA	AA	AA	AA	AA	1	1	1	1	1	1
1534	CH6MM71	6	98790763		AA	AA	AA	AA	AA	AA	AA	AA	1	1	1	1	1	1
1464	CH6MM72	6	101122513		AA	AA	AA	AB	AB	AA	AB	AB	1	0,5	0,5	1	0,5	0,5
550	CH6MM73	6	104128964		AA	AA	AA	AB	AB	AA	AB	AB	1	0,5	0,5	1	0,5	0,5
1751	CH6MM74	6	105270851		BB	BB	BB	BB	BB	BB	BB	BB	1	1	1	1	1	1
1655	CH6MM75	6	105584047		AA	AA	AA	AA	AA	AA	AA	AA	1	1	1	1	1	1
551	CH6MM76	6	106695156		BB	BB	BB	BB	BB	BB	BB	BB	1	1	1	1	1	1
1177	CH6MM77	6	110397055		AA	AA	AA	AA	AA	AA	AA	AA	1	1	1	1	1	1
297	CH6MM78	6	110740281		AA	AA	AA	AA	AA	AA	AA	AA	1	1	1	1	1	1
552	CH6MM79	6	111749805		BB	BB	BB	BB	BB	BB	BB	BB	1	1	1	1	1	1
553	CH6MM80	6	113036388		AA	AA	AA	AA	AA	AA	AA	AA	1	1	1	1	1	1
1954	CH6MM81	6	114932370		BB	BB	BB	AB	BB	BB	BB	BB	1	0,5	1	1	1	1
1966	CH6MM82	6	116477552		AA	AA	AA	AB	AA	AA	AA	AA	1	0,5	1	1	1	1
246	CH6MM83	6	117147180		AA	AA	AA	AB	AA	AA	AA	AA	1	0,5	1	1	1	1
554	CH6MM84	6	118461935		AA	AA	AA	AB	AA	AA	AA	AA	1	0,5	1	1	1	1
1312	CH6MM85	6	120403722		AA	AA	AA	AB	AA	AA	AA	AA	1	0,5	1	1	1	1
1062	CH6MM86	6	120978168		BB	BB	BB	AB	BB	BB	BB	BB	1	0,5	1	1	1	1
273	CH6MM87	6	121702158		AA	AA	AA	AB	AA	AA	AA	AA	1	0,5	1	1	1	1
175	CH6MM88	6	124630688		BB	BB	BB	BB	BB	BB	BB	BB	1	1	1	1	1	1
555	CH6MM89	6	125666374		AA	AA	AA	AA	AA	AA	AA	AA	1	1	1	1	1	1
1745	CH6MM90	6	126340874		AA	AA	AA	AB	AA	AB	AA	AA	1	0,5	1	0,5	1	1
1032	CH6MM91	6	127873902		AA	AA	AA	AA	AA	AA	AA	AA	1	1	1	1	1	1
1241	CH6MM92	6	128654310		AA	AA	AA	AA	AA	AA	AA	AA	1	1	1	1	1	1
91	CH6MM93	6	130920075		AA	AA	AA	AA	AA	AA	AA	AA	1	1	1	1	1	1
1489	CH6MM94	6	131616421		BB	BB	BB	BB	BB	BB	BB	BB	1	1	1	1	1	1
1367	CH6MM95	6	132322704		BB	BB	BB	BB	BB	BB	BB	BB	1	1	1	1	1	1
1886	CH6MM96	6	134019679		AA	AA	AA	AA	AA	AA	AA	AA	1	1	1	1	1	1
556	CH6MM97	6	134303922		AA	AA	AA	AA	AA	AA	AA	AA	1	1	1	1	1	1
1033	CH6MM98	6	135439528		BB	BB	BB	BB	BB	BB	BB	BB	1	1	1	1	1	1
557	CH6MM99	6	136451163		BB	BB	BB	BB	BB	BB	BB	BB	1	1	1	1	1	1
994	CH6MM100	6	137536205		AA	AA	AA	AA	AA	AA	AA	AA	1	1	1	1	1	1
993	CH6MM101	6	137536366		AA	AA	AA	AA	AA	AA	AA	AA	1	1	1	1	1	1
977	CH6MM102	6	137549593		AA	AA	AA	AA	AA	AA	AA	AA	1	1	1	1	1	1
978	CH6MM103	6	137550371		AA	AA	AA	AA	AA	AA	AA	AA	1	1	1	1	1	1
987	CH6MM104	6	137580655		AA	AA	AA	AA	AA	AA	AA	AA	1	1	1	1	1	1
975	CH6MM105	6	137588543		AA	AA	AA	AA	AA	AA	AA	AA	1	1	1	1	1	1
976	CH6MM106	6	137590505		AA	AA	AA	AA	AA	AA	AA	AA	1	1	1	1	1	1
992	CH6MM107	6	137594459		AA	AA	AA	AA	AA	AA	AA	AA	1	1	1	1	1	1
974	CH6MM108	6	137600087		AA	AA	AA	AA	AA	AA	AA	AA	1	1	1	1	1	1
973	CH6MM109	6	137600249		AA	AA	AA	AA	AA	AA	AA	AA	1	1	1	1	1	1
991	CH6MM110	6	137618316		AA	AA	AA	AA	AA	AA	AA	AA	1	1	1	1	1	1
985	CH6MM111	6	137629384		BB	BB	BB	BB	BB	BB	BB	BB	1	1	1	1	1	1
983	CH6MM112	6	137632839		BB	BB	BB	BB	BB	BB	BB	BB	1	1	1	1	1	1
986	CH6MM113	6	137632902		BB	BB	BB	BB	BB	BB	BB	BB	1	1	1	1	1	1
990	CH6MM114	6	137633746		BB	BB	BB	BB	BB	BB	BB	BB	1	1	1	1	1	1
984	CH6MM115	6	137635555		BB	BB	BB	BB	BB	BB	BB	BB	1	1	1	1	1	1
980	CH6MM116	6	137641047		BB	BB	BB	BB	BB	BB	BB	BB	1	1	1	1	1	1
981	CH6MM117	6	137641188		BB	BB	BB	BB	BB	BB	BB	BB	1	1	1	1	1	1
989	CH6MM118	6	137641717		AA	AA	AA	AA	AA	AA	AA	AA	1	1	1	1	1	1
988	CH6MM119	6	137641887		BB	BB	BB	BB	BB	BB	BB	BB	1	1	1	1	1	1
982	CH6MM120	6	137649664		AA	AA	AA	AA	AA	AA	AA	AA	1	1	1	1	1	1
558	CH6MM121	6	138359897		BB	BB	BB	BB	BB	BB	BB	BB	1	1	1	1	1	1
1195	CH6MM122	6	139965472		BB	BB	BB	BB	BB	BB	BB	BB	1	1	1	1	1	1
1883	CH6MM123	6	142567031		AA	AA	AA	AA	AA	AA	AA	AA	1	1	1	1	1	1
559	CH6MM124	6	144187491		BB	BB	BB	BB	BB	BB	BB	BB	1	1	1	1	1	1
1108	CH6MM125	6	144465978		AA	AA	AA	AA	AA	AA	AA	AA	1	1	1	1	1	1
1946	CH6MM126	6	145879903		AA	AA	AA	AA	AA	AA	AA	AA	1	1	1	1	1	1
560	CH6MM127GM6	6	145916231		AA	AA	AA	AA	AA	AA	AA	AA	1	1	1	1	1	1
1814	CH6MM128	6	147380171		AA	AA	AA	AA	AA	AA	AA	AA	1	1	1	1	1	1
1416	CH6MM129	6	148456534		AA	AA	AA	AA	AA	AA	AA	AA	1	1	1	1	1	1
176	CH6MM130	6	149418695		AA	AA	AA	AA	AA	AA	AA	AA	1	1	1	1	1	1
2	CH7MM1GM1	7	1377		AA	AA	AA	AA	AA	AA	AA	AA	1	1	1	1	1	1
1016	CH7MM2	7	3515934		BB	BB	BB	BB	BB	BB	BB	BB	1	1	1	1	1	1
258	CH7MM3	7	4906617		BB	BB	BB	BB	BB	BB	BB	BB	1	1	1	1	1	1
98	CH7MM4	7	5627457		BB	BB	BB	BB	BB	BB	BB	BB	1	1	1	1	1	1
1207	CH7MM5	7	7713719		BB	BB	BB	BB	BB	BB	BB	BB	1	1	1	1	1	1
1503	CH7MM6	7	7950972		BB	BB	BB	BB	BB	BB	BB	BB	1	1	1	1	1	1
561	CH7MM7	7	8241486		AA	AA	AA	AA	AA	AA	AA	AA	1	1	1	1	1	1
1910	CH7MM8	7	9296425		AA	AA	AA	AA	AA	AA	AA	AA	1	1	1	1	1	1
1280	CH7MM9	7	10546409		BB	BB	BB	BB	BB	BB	BB	BB	1	1	1	1	1	1
177	CH7MM10	7	10926083		AA	AA	AA	AA	AA	AA	AA	AA	1	1	1	1	1	1
1065	CH7MM11GM2	7	12204262		AA	AA	AA	AA	AA	AA	AA	AA	1	1	1	1	1	1
1444	CH7MM12	7	12206182		AA	AA	AA	AA	AA	AA	AA	AA	1	1	1	1	1	1
1938	CH7MM13	7	12916620		AA	AA	AA	AA	AA	AA	AA	AA	1	1	1	1	1	1
562	CH7MM14	7	13677268		AA	AA	AA	AA	AA	AA	AA	AA	1	1	1	1	1	1
950	CH7MM15GM3	7	15155585		AA	AA	AA	AA	AA	AA	AA	AA	1	1	1	1	1	1
96	CH7MM16	7	17114061		AA	AA	AA	AA	AA	AA	AA	AA	1	1	1	1	1	1
1605	CH7MM17	7	17800749		BB	BB	BB	BB	BB	BB	BB	BB	1	1	1	1	1	1
277	CH7MM18	7	17994561		BB	BB	BB	BB	BB	BB	BB	BB	1	1	1	1	1	1
1606	CH7MM19	7	18758740		BB	BB	BB	BB	BB	BB	BB	BB	1	1	1	1	1	1
1060	CH7MM20	7	19496283		AA	AA	AA	AA	AA	AA	AA	AA	1	1	1	1	1	1
1471	CH7MM21	7	22647606		BB	BB	BB	BB	BB	BB	BB	BB	1	1	1	1	1	1
1786	CH7MM22	7	23113384		AA	AA	AA	AA	AA	AA	AA	AA	1	1	1	1	1	1
949	CH7MM23	7	23342989		BB	BB	BB	BB	BB	BB	BB	BB	1	1	1	1	1	1
2035	CH7MM24	7	24291067		BB	BB	BB	BB	BB	BB	BB	BB	1	1	1	1	1	1
563	CH7MM25	7	24555108		BB	BB	BB	BB	BB	BB	BB	BB	1	1	1	1	1	1
1478	CH7MM26	7	25217824		AA	AA	AA	AA	AA	AA	AA	AA	1	1	1	1	1	1
1110	CH7MM27	7	25331885		BB	BB	BB	BB	BB	BB	BB	BB	1	1	1	1	1	1
953	CH7MM28BLK2	7	26946160	X	BB	AA	AA	AA	AA	AA	AA	AA	1	1	1	1	1	1
1180	CH7MM29	7	27871533		BB	BB	BB	BB	BB	BB	BB	BB	1	1	1	1	1	1
178	CH7MM30	7	28379711		AA	AA	AA	AA	AA	AA	AA	AA	1	1	1	1	1	1
1628	CH7MM31	7	28755779		AA	AA	AA	AA	AA	AA	AA	AA	1	1	1	1	1	1
970	CH7MM32	7	30187962		BB	BB	BB	BB	BB	BB	BB	BB	1	1	1	1	1	1
971	CH7MM33	7	30190730		AA	AA	AA	AA	AA	AA	AA	AA	1	1	1	1	1	1
954	CH7MM34	7	30192437		BB	BB	BB	BB	BB	BB	BB	BB	1	1	1	1	1	1
955	CH7MM35	7	30192822		BB	BB	BB	BB	BB	BB	BB	BB	1	1	1	1	1	1
2020	CH7MM36	7	30423354		BB	BB	BB	BB	BB	BB	BB	BB	1	1	1	1	1	1
2019	CH7MM37	7	30424935		BB	BB	BB	BB	BB	BB	BB	BB	1	1	1	1	1	1
1136	CH7MM38GM4	7	30432017		BB	BB	BB	BB	BB	BB	BB	BB	1	1	1	1	1	1
1469	CH7MM39	7	31933809		AA	AA	AA	AA	AA	AA	AA	AA	1	1	1	1	1	1
1772	CH7MM40	7	32285087		AA	AA	AA	AA	AA	AA	AA	AA	1	1	1	1	1	1
1034	CH7MM41	7	33634131		BB	BB	BB	BB	BB	BB	BB	BB	1	1	1	1	1	1
2018	CH7MM42	7	33835314		AA	AA	AA	AA	AA	AA	AA	AA	1	1	1	1	1	1
97	CH7MM43	7	36725559		AA	AA	AA	AA	AA	AA	AA	AA	1	1	1	1	1	1
1103	CH7MM44	7	37176366		AA	AA	AA	AA	AA	AA	AA	AA	1	1	1	1	1	1
1345	CH7MM45	7	38476481		BB	BB	BB	BB	BB	BB	BB	BB	1	1	1	1	1	1
1347	CH7MM46	7	38982522		AA	AA	AA	AA	AA	AA	AA	AA	1	1	1	1	1	1
564	CH7MM47	7	39863901		BB	BB	BB	BB	BB	BB	BB	BB	1	1	1	1	1	1
1461	CH7MM48	7	41170581		AA	AA	AA	AA	AA	AA	AA	AA	1	1	1	1	1	1
1140	CH7MM49GM5	7	43718739		BB	BB	BB	BB	BB	BB	BB	BB	1	1	1	1	1	1
995	CH7MM50	7	43997477		BB	BB	BB	BB	BB	BB	BB	BB	1	1	1	1	1	1
1314	CH7MM51	7	44701332		AA	AA	AA	AA	AA	AA	AA	AA	1	1	1	1	1	1
1067	CH7MM52GM6	7	45173762		AA	AA	AA	AA	AA	AA	AA	AA	1	1	1	1	1	1
2000	CH7MM53	7	46099541		AA	AA	AA	AA	AA	AA	AA	AA	1	1	1	1	1	1
565	CH7MM54	7	46313106		AA	AA	AA	AA	AA	AA	AA	AA	1	1	1	1	1	1
253	CH7MM55	7	47949709		AA	AA	AA	AA	AA	AA	AA	AA	1	1	1	1	1	1
1651	CH7MM56	7	49980287		BB	BB	BB	BB	BB	BB	BB	BB	1	1	1	1	1	1
1234	CH7MM57	7	51392430		AA	AA	AA	AA	AA	AA	AA	AA	1	1	1	1	1	1
1443	CH7MM58	7	51544526		BB	BB	BB	BB	BB	BB	BB	BB	1	1	1	1	1	1
1706	CH7MM59	7	53312532		BB	BB	BB	BB	BB	BB	BB	BB	1	1	1	1	1	1
1371	CH7MM60	7	55557090		BB	BB	BB	BB	BB	BB	BB	BB	1	1	1	1	1	1
566	CH7MM61	7	56830620		AA	AA	AA	AA	AA	AA	AA	AA	1	1	1	1	1	1
2017	CH7MM62	7	57727397		AA	AA	AA	AA	AA	AA	AA	AA	1	1	1	1	1	1
567	CH7MM63	7	57878499		AA	AA	AA	AA	AA	AA	AA	AA	1	1	1	1	1	1
568	CH7MM64	7	58334612		BB	BB	BB	BB	BB	BB	BB	BB	1	1	1	1	1	1

1211	CH7MM65	7	59722158		BB	BB	BB	BB	BB	BB	BB	BB	1	1	1	1	1	1
569	CH7MM66	7	61105319		BB	BB	BB	BB	BB	BB	BB	BB	1	1	1	1	1	1
1223	CH7MM67	7	61680107		BB	BB	BB	BB	BB	BB	BB	BB	1	1	1	1	1	1
570	CH7MM68	7	63356038		BB	BB	BB	BB	BB	BB	BB	BB	1	1	1	1	1	1
571	CH7MM69	7	65348455		BB	BB	BB	BB	BB	BB	BB	BB	1	1	1	1	1	1
572	CH7MM70	7	66776784		AA	AA	AA	AA	AA	AA	AA	AA	1	1	1	1	1	1
573	CH7MM71	7	68228570		AA	AA	AA	AA	AA	AA	AA	AA	1	1	1	1	1	1
1758	CH7MM72	7	69422276		AA	AA	AA	AA	AA	AA	AA	AA	1	1	1	1	1	1
574	CH7MM73	7	71406934		AA	AA	AA	AA	AA	AA	AA	AA	1	1	1	1	1	1
1015	CH7MM74	7	72820733		AA	AA	AA	AA	AA	AA	AA	AA	1	1	1	1	1	1
575	CH7MM75	7	74292260		AA	AA	AA	AA	AA	AA	AA	AA	1	1	1	1	1	1
576	CH7MM76	7	75869041		AA	AA	AA	AA	AA	AA	AA	AA	1	1	1	1	1	1
577	CH7MM77	7	77234500		BB	BB	BB	BB	BB	BB	BB	BB	1	1	1	1	1	1
99	CH7MM78	7	77850273		AA	AA	AA	AA	AA	AA	AA	AA	1	1	1	1	1	1
1657	CH7MM79	7	79257238		BB	BB	BB	BB	BB	BB	BB	BB	1	1	1	1	1	1
1607	CH7MM80	7	80158909		BB	BB	BB	BB	BB	BB	BB	BB	1	1	1	1	1	1
1830	CH7MM81	7	80917494		AA	AA	AA	AA	AA	AA	AA	AA	1	1	1	1	1	1
579	CH7MM82	7	82727616		BB	BB	BB	BB	BB	BB	BB	BB	1	1	1	1	1	1
1335	CH7MM83	7	83322675		BB	BB	BB	BB	BB	BB	BB	BB	1	1	1	1	1	1
1387	CH7MM84	7	85992410		BB	BB	BB	BB	BB	BB	BB	BB	1	1	1	1	1	1
580	CH7MM85	7	87305015		BB	BB	BB	BB	BB	BB	BB	BB	1	1	1	1	1	1
1433	CH7MM86	7	89115174		AA	AA	AA	AA	AA	AA	AA	AA	1	1	1	1	1	1
1107	CH7MM87GM7	7	89489356		BB	BB	BB	BB	BB	BB	BB	BB	1	1	1	1	1	1
1903	CH7MM88	7	89751283		BB	BB	BB	BB	BB	BB	BB	BB	1	1	1	1	1	1
578	CH7MM89GM8	7	91697692		AA	AA	AA	AA	AA	AA	AA	AA	1	1	1	1	1	1
1199	CH7MM90	7	94344049		AA	AA	AA	AA	AA	AA	AA	AA	1	1	1	1	1	1
581	CH7MM91	7	94635643		AA	AA	AA	AA	AA	AA	AA	AA	1	1	1	1	1	1
285	CH7MM92GM9-BM	7	94641553		AA	AA	AA	AA	AA	AA	AA	AA	1	1	1	1	1	1
1943	CH7MM93	7	94870318		BB	BB	BB	BB	BB	BB	BB	BB	1	1	1	1	1	1
582	CH7MM94	7	96728523		AA	AA	AA	AA	AA	AA	AA	AA	1	1	1	1	1	1
1631	CH7MM95	7	96778482		AA	AA	AA	AA	AA	AA	AA	AA	1	1	1	1	1	1
962	CH7MM96	7	98626884		BB	BB	BB	BB	BB	BB	BB	BB	1	1	1	1	1	1
583	CH7MM97	7	99220204		BB	BB	BB	BB	BB	BB	BB	BB	1	1	1	1	1	1
1396	CH7MM98	7	99278326		BB	BB	BB	BB	BB	BB	BB	BB	1	1	1	1	1	1
1506	CH7MM99	7	101325771		BB	BB	BB	BB	BB	BB	BB	BB	1	1	1	1	1	1
1736	CH7MM100	7	101647757		BB	BB	BB	BB	BB	BB	BB	BB	1	1	1	1	1	1
584	CH7MM101	7	104533846		BB	BB	BB	BB	BB	BB	BB	BB	1	1	1	1	1	1
1035	CH7MM102	7	106579694		AA	AA	AA	AA	AA	AA	AA	AA	1	1	1	1	1	1
266	CH7MM103	7	107252093		BB	BB	BB	BB	BB	BB	BB	BB	1	1	1	1	1	1
1406	CH7MM104	7	107424656		BB	BB	BB	BB	BB	BB	BB	BB	1	1	1	1	1	1
959	CH7MM105	7	109028859		AA	AA	AA	AA	AA	AA	AA	AA	1	1	1	1	1	1
585	CH7MM106	7	109865120		AA	AA	AA	AA	AA	AA	AA	AA	1	1	1	1	1	1
1825	CH7MM107	7	111285662		BB	BB	BB	BB	BB	BB	BB	BB	1	1	1	1	1	1
586	CH7MM108	7	112152410		BB	BB	BB	BB	BB	BB	BB	BB	1	1	1	1	1	1
1671	CH7MM109	7	113779795		BB	BB	BB	BB	BB	BB	BB	BB	1	1	1	1	1	1
1243	CH7MM110	7	113814867		BB	BB	BB	BB	BB	BB	BB	BB	1	1	1	1	1	1
587	CH7MM111	7	114579244		BB	BB	BB	BB	BB	BB	BB	BB	1	1	1	1	1	1
588	CH7MM112	7	115461663		AA	AA	AA	AA	AA	AA	AA	AA	1	1	1	1	1	1
1069	CH7MM113GM10	7	115892950		BB	BB	BB	BB	BB	BB	BB	BB	1	1	1	1	1	1
589	CH7MM114BLK8	7	116540646	X	BB	AA	AA	AA	AA	AA	AA	AA	1	1	1	1	1	1
1827	CH7MM115GM11	7	118143195		AA	AA	AA	AA	AA	AA	AA	AA	1	1	1	1	1	1
590	CH7MM116	7	119192048		BB	BB	BB	BB	BB	BB	BB	BB	1	1	1	1	1	1
1450	CH7MM117	7	120621563		AA	AA	AA	AA	AA	AA	AA	AA	1	1	1	1	1	1
1644	CH7MM118	7	120693856		BB	BB	BB	BB	BB	BB	BB	BB	1	1	1	1	1	1
95	CH7MM119	7	122752866		BB	BB	BB	BB	BB	BB	BB	BB	1	1	1	1	1	1
591	CH7MM120	7	124913079		AA	AA	AA	AA	AA	AA	AA	AA	1	1	1	1	1	1
1145	CH7MM121GM12	7	126965988		BB	BB	BB	BB	BB	BB	BB	BB	1	1	1	1	1	1
1667	CH7MM122	7	128389117		AA	AA	AA	AA	AA	AA	AA	AA	1	1	1	1	1	1
2016	CH7MM123	7	130979090		BB	BB	BB	BB	BB	BB	BB	BB	1	1	1	1	1	1
1761	CH7MM124	7	132894685		BB	BB	BB	BB	BB	BB	BB	BB	1	1	1	1	1	1
948	CH7MM125	7	142377517		AA	AA	AA	AA	AA	AA	AA	AA	1	1	1	1	1	1
1992	CH7MM126	7	144933893		AA	AA	AA	AA	AA	AA	AA	AA	1	1	1	1	1	1
1993	CH7MM127	7	144934224		BB	BB	BB	BB	BB	BB	BB	BB	1	1	1	1	1	1
1422	CH8MM1	8	4193008		AA	AA	AA	AA	AA	AA	AA	AA	1	1	1	1	1	1
1909	CH8MM2	8	6795642		BB	BB	BB	BB	BB	BB	BB	BB	1	1	1	1	1	1
1822	CH8MM3	8	7842210		AA	AA	AA	AA	AA	AA	AA	AA	1	1	1	1	1	1
1700	CH8MM4	8	8294541		AA	AA	AA	AA	AA	AA	AA	AA	1	1	1	1	1	1
1690	CH8MM5	8	8846707		BB	BB	BB	BB	BB	BB	BB	BB	1	1	1	1	1	1
592	CH8MM6	8	11675148		AA	AA	AA	AA	AA	AA	AA	AA	1	1	1	1	1	1
1979	CH8MM7	8	14327885		BB	BB	BB	BB	BB	BB	BB	BB	1	1	1	1	1	1
593	CH8MM8	8	15319466		BB	BB	BB	BB	BB	BB	BB	BB	1	1	1	1	1	1
594	CH8MM9	8	16273703		BB	BB	BB	BB	BB	BB	BB	BB	1	1	1	1	1	1
1099	CH8MM10	8	16576750		BB	BB	BB	BB	BB	BB	BB	BB	1	1	1	1	1	1
179	CH8MM11	8	18169937		BB	BB	BB	BB	BB	BB	BB	BB	1	1	1	1	1	1
1541	CH8MM12	8	21285113		BB	BB	BB	BB	BB	BB	BB	BB	1	1	1	1	1	1
180	CH8MM13	8	21995114		AA	AA	AA	AA	AA	AA	AA	AA	1	1	1	1	1	1
1127	CH8MM14	8	22251497		BB	BB	BB	BB	BB	BB	BB	BB	1	1	1	1	1	1
595	CH8MM15	8	24309617		AA	AA	AA	AA	AA	AA	AA	AA	1	1	1	1	1	1
1368	CH8MM16	8	25083165		AA	AA	AA	AA	AA	AA	AA	AA	1	1	1	1	1	1
100	CH8MM17	8	25963079		BB	BB	BB	BB	BB	BB	BB	BB	1	1	1	1	1	1
14	CH8MM18BLK4	8	26496123	X	BB	AA	AA	AA	AA	AA	AA	AA	1	1	1	1	1	1
181	CH8MM19	8	27236274		AA	AA	AA	AA	AA	AA	AA	AA	1	1	1	1	1	1
596	CH8MM20	8	28075869		BB	BB	BB	BB	BB	BB	BB	BB	1	1	1	1	1	1
597	CH8MM21GM1	8	29641108		AA	AA	AA	AA	AA	AA	AA	AA	1	1	1	1	1	1
1608	CH8MM22	8	30204827		AA	AA	AA	AA	AA	AA	AA	AA	1	1	1	1	1	1
1385	CH8MM23	8	30816540		BB	BB	BB	BB	BB	BB	BB	BB	1	1	1	1	1	1
1300	CH8MM24	8	33507144		AA	AA	AA	AA	AA	AA	AA	AA	1	1	1	1	1	1
101	CH8MM25	8	33812776		AA	AA	AA	AA	AA	AA	AA	AA	1	1	1	1	1	1
1153	CH8MM26	8	35164032		AA	AA	AA	AA	AA	AA	AA	AA	1	1	1	1	1	1
1168	CH8MM27	8	37536463		BB	BB	BB	BB	BB	BB	BB	BB	1	1	1	1	1	1
598	CH8MM28	8	38775830		BB	BB	BB	BB	BB	BB	BB	BB	1	1	1	1	1	1
1366	CH8MM29	8	39770568		BB	BB	BB	BB	BB	BB	BB	BB	1	1	1	1	1	1
1159	CH8MM30	8	42081791		AA	AA	AA	AA	AA	AA	AA	AA	1	1	1	1	1	1
1941	CH8MM31	8	43934856		AA	AA	AA	AA	AA	AA	AA	AA	1	1	1	1	1	1
599	CH8MM32	8	44684826		AA	AA	AA	AA	AA	AA	AA	AA	1	1	1	1	1	1
1473	CH8MM33	8	48804736		BB	BB	BB	BB	BB	BB	BB	BB	1	1	1	1	1	1
600	CH8MM34	8	49714626		AA	AA	AA	AA	AA	AA	AA	AA	1	1	1	1	1	1
601	CH8MM35	8	50117838		AA	AA	AA	AA	AA	AA	AA	AA	1	1	1	1	1	1
102	CH8MM36	8	51076519		AA	AA	AA	AA	AA	AA	AA	AA	1	1	1	1	1	1
103	CH8MM37	8	51607005		BB	BB	BB	BB	BB	BB	BB	BB	1	1	1	1	1	1
602	CH8MM38	8	53532210		BB	BB	BB	BB	BB	BB	BB	BB	1	1	1	1	1	1
1076	CH8MM39GM2	8	54059055		AA	AA	AA	AA	AA	AA	AA	AA	1	1	1	1	1	1
603	CH8MM40	8	55177326		AA	AA	AA	AA	AA	AA	AA	AA	1	1	1	1	1	1
1510	CH8MM41	8	56141367		BB	BB	BB	BB	BB	BB	BB	BB	1	1	1	1	1	1
1261	CH8MM42	8	56548348		BB	BB	BB	BB	BB	BB	BB	BB	1	1	1	1	1	1
604	CH8MM43GM3	8	57536068		BB	BB	BB	BB	BB	BB	BB	BB	1	1	1	1	1	1
1390	CH8MM44	8	57724335		BB	BB	BB	BB	BB	BB	BB	BB	1	1	1	1	1	1
1192	CH8MM45	8	60120308		BB	BB	BB	BB	BB	BB	BB	BB	1	1	1	1	1	1
605	CH8MM46	8	60901130		AA	AA	AA	AA	AA	AA	AA	AA	1	1	1	1	1	1
1165	CH8MM47	8	62920755		AA	AA	AA	AA	AA	AA	AA	AA	1	1	1	1	1	1
1922	CH8MM48	8	63043201		AA	AA	AA	AA	AA	AA	AA	AA	1	1	1	1	1	1
606	CH8MM49	8	64586401		AA	AA	AA	AA	AA	AA	AA	AA	1	1	1	1	1	1
1638	CH8MM50	8	65821143		BB	BB	BB	BB	BB	BB	BB	BB	1	1	1	1	1	1
607	CH8MM51	8	66034595		AA	AA	AA	AA	AA	AA	AA	AA	1	1	1	1	1	1
1095	CH8MM52	8	67222604		AA	AA	AA	AA	AA	AA	AA	AA	1	1	1	1	1	1
1052	CH8MM53	8	68083563		AA	AA	AA	AA	AA	AA	AA	AA	1	1	1	1	1	1
1340	CH8MM54	8	68996069		BB	BB	BB	BB	BB	BB	BB	BB	1	1	1	1	1	1
2004	CH8MM55	8	69827200		AA	AA	AA	AA	AA	AA	AA	AA	1	1	1	1	1	1
1167	CH8MM56	8	70497527		BB	BB	BB	BB	BB	BB	BB	BB	1	1	1	1	1	1
608	CH8MM57	8	72013995		BB	BB	BB	BB	BB	BB	BB	BB	1	1	1	1	1	1
1609	CH8MM58	8	74193741		AA	AA	AA	AA	AA	AA	AA	AA	1	1	1	1	1	1
1318	CH8MM59	8	74806749		BB	BB	BB	BB	BB	BB	BB	BB	1	1	1	1	1	1
1664	CH8MM60	8	75293484		AA	AA	AA	AA	AA	AA	AA	AA	1	1	1	1	1	1
1329	CH8MM61	8	77144203		AA	AA	AA	AA	AA	AA	AA	AA	1	1	1	1	1	1
140	CH8MM62	8	78291790		BB	BB	BB	BB	BB	BB	BB	BB	1	1	1	1	1	1
1850	CH8MM63	8	79152010		AA	AA	AA	AA	AA	AA	AA	AA	1	1	1	1	1	1
1610	CH8MM64	8	80165835		AA	AA	AA	AA	AA	AA	AA	AA	1	1	1	1	1	1

1002	CH8MM65	8	82055179		AA	AA	AA	AA	AA	AA	AA	AA	1	1	1	1	1	1
609	CH8MM66	8	82188194		BB	BB	BB	BB	BB	BB	BB	BB	1	1	1	1	1	1
610	CH8MM67	8	84344531		BB	BB	BB	BB	BB	BB	BB	BB	1	1	1	1	1	1
1808	CH8MM68	8	84826788		AA	AA	AA	AA	AA	AA	AA	AA	1	1	1	1	1	1
611	CH8MM69	8	86040626		AA	AA	AA	AA	AA	AA	AA	AA	1	1	1	1	1	1
612	CH8MM70	8	86397354		BB	BB	BB	BB	BB	BB	BB	BB	1	1	1	1	1	1
613	CH8MM71	8	86954362		BB	BB	BB	BB	BB	BB	BB	BB	1	1	1	1	1	1
1951	CH8MM72	8	89169668		AA	AA	BB	AA	BB	AB	AB	BB	0	1	0	0,5	0,5	0
1072	CH8MM73BLK12	8	90227633	X	BB	AA	BB	AA	BB	AB	AB	BB	0	1	0	0,5	0,5	0
614	CH8MM74	8	91879489		BB	BB	AA	BB	AA	AB	AB	AA	0	1	0	0,5	0,5	0
1530	CH8MM75	8	94037444		AA	AA	AA	AA	AA	AA	AA	AA	1	1	1	1	1	1
1834	CH8MM76	8	95323284		AA	AA	BB	AB	BB	AB	AB	BB	0	0,5	0	0,5	0,5	0
1129	CH8MM77	8	95633139		BB	BB	BB	BB	BB	BB	BB	BB	1	1	1	1	1	1
1836	CH8MM78	8	96735663		AA	AA	BB	AB	BB	AB	AB	BB	0	0,5	0	0,5	0,5	0
615	CH8MM79	8	98654267		AA	AA	BB	AB	BB	AB	AB	BB	0	0,5	0	0,5	0,5	0
1377	CH8MM80	8	100766333		BB	BB	AA	AB	AA	AB	AB	AA	0	0,5	0	0,5	0,5	0
1170	CH8MM81	8	101399186		AA	AA	BB	AB	BB	AB	AB	BB	0	0,5	0	0,5	0,5	0
616	CH8MM82	8	101681071		BB	BB	AA	AB	AA	AB	AB	AA	0	0,5	0	0,5	0,5	0
1372	CH8MM83	8	102686478		AA	AA	AA	AA	AA	AA	AA	AA	1	1	1	1	1	1
182	CH8MM84	8	106117382		AA	AA	AA	AA	AA	AA	AA	AA	1	1	1	1	1	1
183	CH8MM85	8	108081601		BB	BB	BB	BB	BB	BB	BB	BB	1	1	1	1	1	1
1134	CH8MM86	8	108727362		AA	AA	AA	AA	AA	AA	AA	AA	1	1	1	1	1	1
1782	CH8MM87	8	109333466		AA	AA	AA	AA	AA	AA	AA	AA	1	1	1	1	1	1
617	CH8MM88	8	113066370		AA	AA	AA	AA	AA	AA	AA	AA	1	1	1	1	1	1
1611	CH8MM89	8	113744792		BB	BB	BB	BB	BB	BB	BB	BB	1	1	1	1	1	1
184	CH8MM90	8	116198601		AA	AA	AA	AA	AA	AA	AA	AA	1	1	1	1	1	1
1017	CH8MM91	8	116907245		BB	BB	BB	BB	BB	BB	BB	BB	1	1	1	1	1	1
618	CH8MM92	8	118001545		BB	BB	BB	BB	BB	BB	BB	BB	1	1	1	1	1	1
1395	CH8MM93	8	121235325		AA	AA	AA	AA	AA	AA	AA	AA	1	1	1	1	1	1
1928	CH8MM94	8	121825605		BB	BB	BB	BB	BB	BB	BB	BB	1	1	1	1	1	1
1375	CH8MM95	8	123350039		AA	AA	AA	AA	AA	AA	AA	AA	1	1	1	1	1	1
1654	CH8MM96	8	125496048		BB	BB	BB	BB	BB	BB	BB	BB	1	1	1	1	1	1
1963	CH8MM97	8	125966003		AA	AA	AA	AA	AA	AA	AA	AA	1	1	1	1	1	1
1327	CH8MM98	8	127236890		AA	AA	AA	AA	AA	AA	AA	AA	1	1	1	1	1	1
1077	CH9MM1BLK1	9	6238770	X	BB	AA	AA	AA	AA	AA	AA	AA	1	1	1	1	1	1
247	CH9MM2	9	6793269		BB	BB	BB	BB	BB	BB	BB	BB	1	1	1	1	1	1
965	CH9MM3	9	8898245		BB	BB	BB	BB	BB	BB	BB	BB	1	1	1	1	1	1
619	CH9MM4	9	9289357		BB	BB	BB	BB	BB	BB	BB	BB	1	1	1	1	1	1
276	CH9MM5	9	10587580		AA	AA	AA	AA	AA	AA	AA	AA	1	1	1	1	1	1
267	CH9MM6	9	12387092		BB	BB	BB	BB	BB	BB	BB	BB	1	1	1	1	1	1
620	CH9MM7	9	13255595		BB	BB	BB	BB	BB	BB	BB	BB	1	1	1	1	1	1
293	CH9MM8	9	14023134		AA	AA	AA	AA	AA	AA	AA	AA	1	1	1	1	1	1
185	CH9MM9	9	17881329		BB	BB	BB	BB	BB	BB	BB	BB	1	1	1	1	1	1
621	CH9MM10	9	18988599		AA	AA	AA	AA	AA	AA	AA	AA	1	1	1	1	1	1
2026	CH9MM11	9	21900659		BB	BB	BB	BB	BB	BB	BB	BB	1	1	1	1	1	1
622	CH9MM12	9	23359729		BB	BB	BB	BB	BB	BB	BB	BB	1	1	1	1	1	1
623	CH9MM13	9	24740593		AA	AA	AA	AA	AA	AA	AA	AA	1	1	1	1	1	1
1049	CH9MM14	9	24949186		AA	AA	AA	AA	AA	AA	AA	AA	1	1	1	1	1	1
624	CH9MM15	9	26555143		BB	BB	BB	BB	BB	BB	BB	BB	1	1	1	1	1	1
1268	CH9MM16	9	26774587		AA	AA	AA	AA	AA	AA	AA	AA	1	1	1	1	1	1
1973	CH9MM17	9	28016429		AA	AA	AA	AA	AA	AA	AA	AA	1	1	1	1	1	1
1939	CH9MM18	9	29608871		AA	AA	AA	AA	AA	AA	AA	AA	1	1	1	1	1	1
1689	CH9MM19	9	29828396		BB	BB	BB	BB	BB	BB	BB	BB	1	1	1	1	1	1
104	CH9MM20	9	29909656		AA	AA	AA	AA	AA	AA	AA	AA	1	1	1	1	1	1
966	CH9MM21	9	31107154		AA	AA	AA	AA	AA	AA	AA	AA	1	1	1	1	1	1
3	CH9MM22GM2	9	32742143		AA	AA	AA	AA	AA	AA	AA	AA	1	1	1	1	1	1
1155	CH9MM23	9	32825296		BB	BB	BB	BB	BB	BB	BB	BB	1	1	1	1	1	1
292	CH9MM24	9	33481487		BB	BB	BB	BB	BB	BB	BB	BB	1	1	1	1	1	1
1092	CH9MM25	9	33657166		AA	AA	AA	AA	AA	AA	AA	AA	1	1	1	1	1	1
1423	CH9MM26	9	34437206		AA	AA	AA	AA	AA	AA	AA	AA	1	1	1	1	1	1
2053	CH9MM27	9	35184595		AA	AA	AA	AA	AA	AA	AA	AA	1	1	1	1	1	1
625	CH9MM28	9	36959921		AA	AA	AA	AA	AA	AA	AA	AA	1	1	1	1	1	1
1749	CH9MM29	9	37083126		AA	AA	AA	AA	AA	AA	AA	AA	1	1	1	1	1	1
1983	CH9MM30	9	37941444		BB	BB	BB	BB	BB	BB	BB	BB	1	1	1	1	1	1
626	CH9MM31	9	39740553		BB	BB	BB	BB	BB	BB	BB	BB	1	1	1	1	1	1
1441	CH9MM32	9	40768895		BB	BB	BB	BB	BB	BB	BB	BB	1	1	1	1	1	1
1524	CH9MM33	9	43060131		AA	AA	AA	AA	AA	AA	AA	AA	1	1	1	1	1	1
317	CH9MM34	9	44740915		AA	AA	AA	AA	AA	AA	AA	AA	1	1	1	1	1	1
1692	CH9MM35	9	44908589		BB	BB	BB	BB	BB	BB	BB	BB	1	1	1	1	1	1
2034	CH9MM36	9	45183767		BB	BB	BB	BB	BB	BB	BB	BB	1	1	1	1	1	1
627	CH9MM37	9	46633516		AA	AA	AA	AA	AA	AA	AA	AA	1	1	1	1	1	1
1957	CH9MM38	9	47519917		AA	AA	AA	AA	AA	AA	AA	AA	1	1	1	1	1	1
186	CH9MM39	9	48315615		AA	AA	AA	AA	AA	AA	AA	AA	1	1	1	1	1	1
105	CH9MM40	9	49183636		AA	AA	AA	AA	AA	AA	AA	AA	1	1	1	1	1	1
1407	CH9MM41	9	49384481		AA	AA	AA	AA	AA	AA	AA	AA	1	1	1	1	1	1
628	CH9MM42	9	50318725		BB	BB	BB	BB	BB	BB	BB	BB	1	1	1	1	1	1
1497	CH9MM43	9	52682809		AA	AA	AA	AA	AA	AA	AA	AA	1	1	1	1	1	1
629	CH9MM44	9	55395056		BB	BB	BB	BB	BB	BB	BB	BB	1	1	1	1	1	1
1160	CH9MM45	9	56790950		BB	BB	BB	BB	BB	BB	BB	BB	1	1	1	1	1	1
630	CH9MM46	9	58024426		BB	BB	BB	BB	BB	BB	BB	BB	1	1	1	1	1	1
1768	CH9MM47	9	60196412		AA	AA	AA	AA	AA	AA	AA	AA	1	1	1	1	1	1
1905	CH9MM48GM3	9	60303255		AA	AA	AA	AA	AA	AA	AA	AA	1	1	1	1	1	1
1438	CH9MM49	9	61377354		BB	BB	BB	BB	BB	BB	BB	BB	1	1	1	1	1	1
187	CH9MM50	9	63239299		AA	AA	AA	AA	AA	AA	AA	AA	1	1	1	1	1	1
1090	CH9MM51	9	65704935		AA	AA	AA	AA	AA	AA	AA	AA	1	1	1	1	1	1
1717	CH9MM52	9	68381485		BB	BB	BB	BB	BB	BB	BB	BB	1	1	1	1	1	1
1483	CH9MM53	9	71328971		BB	BB	BB	BB	BB	BB	BB	BB	1	1	1	1	1	1
1297	CH9MM54	9	71973325		AA	AA	AA	AA	AA	AA	AA	AA	1	1	1	1	1	1
631	CH9MM55	9	73145678		BB	BB	BB	BB	BB	BB	BB	BB	1	1	1	1	1	1
1508	CH9MM56	9	75221496		BB	BB	BB	BB	BB	BB	BB	BB	1	1	1	1	1	1
632	CH9MM57	9	76908789		BB	BB	BB	BB	BB	BB	BB	BB	1	1	1	1	1	1
2010	CH9MM58	9	78333554		AA	AA	AA	AA	AA	AA	AA	AA	1	1	1	1	1	1
1029	CH9MM59BLK9	9	78899165	X	AA	BB	BB	BB	BB	BB	BB	BB	1	1	1	1	1	1
1807	CH9MM60	9	79059621		AA	AA	AA	AA	AA	AA	AA	AA	1	1	1	1	1	1
188	CH9MM61	9	80458981		BB	BB	BB	BB	BB	BB	BB	BB	1	1	1	1	1	1
1767	CH9MM62	9	82203633		BB	BB	BB	BB	BB	BB	BB	BB	1	1	1	1	1	1
1209	CH9MM63	9	83548399		BB	BB	BB	BB	BB	BB	BB	BB	1	1	1	1	1	1
633	CH9MM64	9	84554360		AA	AA	AA	AA	AA	AA	AA	AA	1	1	1	1	1	1
1304	CH9MM65	9	85584599		BB	BB	BB	BB	BB	BB	BB	BB	1	1	1	1	1	1
1349	CH9MM66	9	86745093		AA	AA	AA	AA	AA	AA	AA	AA	1	1	1	1	1	1
634	CH9MM67	9	87865881		AA	AA	AA	AA	AA	AA	AA	AA	1	1	1	1	1	1
635	CH9MM68	9	88301228		BB	BB	BB	BB	BB	BB	BB	BB	1	1	1	1	1	1
1174	CH9MM69	9	88325825		AA	AA	AA	AA	AA	AA	AA	AA	1	1	1	1	1	1
1828	CH9MM70	9	88646923		BB	BB	BB	BB	BB	BB	BB	BB	1	1	1	1	1	1
189	CH9MM71	9	90513305		BB	BB	BB	BB	BB	BB	BB	BB	1	1	1	1	1	1
636	CH9MM72	9	92060032		AA	AA	AA	AA	AA	AA	AA	AA	1	1	1	1	1	1
1846	CH9MM73GM4	9	92892376		AA	AA	AA	AA	AA	AA	AA	AA	1	1	1	1	1	1
637	CH9MM74	9	94137054		BB	BB	BB	BB	BB	BB	BB	BB	1	1	1	1	1	1
106	CH9MM75	9	95875215		BB	BB	BB	BB	BB	BB	BB	BB	1	1	1	1	1	1
1281	CH9MM76	9	96256381		AA	AA	AA	AA	AA	AA	AA	AA	1	1	1	1	1	1
638	CH9MM77	9	98418600		BB	BB	BB	BB	BB	BB	BB	BB	1	1	1	1	1	1
1940	CH9MM78	9	99710289		AA	AA	AA	AA	AA	AA	AA	AA	1	1	1	1	1	1
1457	CH9MM79	9	101878542		AA	AA	AA	AA	AA	AA	AA	AA	1	1	1	1	1	1
1048	CH9MM80	9	103527865		AA	AA	AA	AA	AA	AA	AA	AA	1	1	1	1	1	1
639	CH9MM81	9	103546788		AA	AA	AA	AA	AA	AA	AA	AA	1	1	1	1	1	1
1417	CH9MM82	9	105418025		AA	AA	AA	AA	AA	AA	AA	AA	1	1	1	1	1	1
1670	CH9MM83	9	105985762		AA	AA	AA	AA	AA	AA	AA	AA	1	1	1	1	1	1
640	CH9MM84	9	106229354		BB	BB	BB	BB	BB	BB	BB	BB	1	1	1	1	1	1
945	CH9MM85	9	108095951		BB	BB	BB	BB	BB	BB	BB	BB	1	1	1	1	1	1
641	CH9MM86	9	108690630		AA	AA	AA	AA	AA	AA	AA	AA	1	1	1	1	1	1
642	CH9MM87	9	109861337		AA	AA	AA	AA	AA	AA	AA	AA	1	1	1	1	1	1
643	CH9MM88	9	111862825		BB	BB	BB	BB	BB	BB	BB	BB	1	1	1	1	1	1
190	CH9MM89	9	113307145		BB	BB	BB	BB	BB	BB	BB	BB	1	1	1	1	1	1
1303	CH9MM90	9	114041353		AA	AA	AA	AA	AA	AA	AA	AA	1	1	1	1	1	1
1699	CH9MM91	9	114111475		BB	BB	BB	BB	BB	BB	BB	BB	1	1	1	1	1	1
1873	CH9MM92	9	115090216		AA	AA	AA	AA	AA	AA	AA	AA	1	1	1	1	1	1
1173	CH9MM93	9	117891342		BB	BB	BB	BB	BB	BB	BB	BB	1	1	1	1	1	1

302	CH9MM94	9	121920390		AA	AA	AA	AA	AA	AA	AA	AA	1	1	1	1	1	1
1855	CH9MM95	9	123162408		BB	BB	BB	BB	BB	BB	BB	BB	1	1	1	1	1	1
1486	CH10MM1	10	3343120		BB	BB	BB	BB	BB	BB	BB	BB	1	1	1	1	1	1
33	CH10MM2	10	3989939		AA	AA	AA	AA	AA	AA	AA	AA	1	1	1	1	1	1
644	CH10MM3	10	4778214		AA	AA	AA	AA	AA	AA	AA	AA	1	1	1	1	1	1
1723	CH10MM4	10	5384467		BB	BB	BB	BB	BB	BB	BB	BB	1	1	1	1	1	1
4	CH10MM5GM1	10	6504894		BB	BB	BB	BB	BB	BB	BB	BB	1	1	1	1	1	1
2008	CH10MM6	10	6788687		BB	BB	BB	BB	BB	BB	BB	BB	1	1	1	1	1	1
1147	CH10MM7	10	9286770		AA	AA	AA	AA	AA	AA	AA	AA	1	1	1	1	1	1
1262	CH10MM8	10	10540887		AA	AA	AA	AA	AA	AA	AA	AA	1	1	1	1	1	1
1612	CH10MM9	10	12719980		BB	BB	BB	BB	BB	BB	BB	BB	1	1	1	1	1	1
1613	CH10MM10	10	14115727		AA	AA	AA	AA	AA	AA	AA	AA	1	1	1	1	1	1
1267	CH10MM11GM2	10	14116427		AA	AA	AA	AA	AA	AA	AA	AA	1	1	1	1	1	1
1341	CH10MM12	10	15499377		BB	BB	BB	BB	BB	BB	BB	BB	1	1	1	1	1	1
1427	CH10MM13	10	17639020		AA	AA	AA	AA	AA	AA	AA	AA	1	1	1	1	1	1
1315	CH10MM14	10	17883074		AA	AA	AA	AA	AA	AA	AA	AA	1	1	1	1	1	1
1643	CH10MM15	10	18812801		BB	BB	BB	BB	BB	BB	BB	BB	1	1	1	1	1	1
645	CH10MM16	10	19318252		BB	BB	BB	BB	BB	BB	BB	BB	1	1	1	1	1	1
303	CH10MM17	10	20027450		AA	AA	AA	AA	AA	AA	AA	AA	1	1	1	1	1	1
32	CH10MM18	10	20959105		BB	BB	BB	BB	BB	BB	BB	BB	1	1	1	1	1	1
1232	CH10MM19	10	22685808		AA	AA	AA	AA	AA	AA	AA	AA	1	1	1	1	1	1
646	CH10MM20	10	24247475		AA	AA	AA	AA	AA	AA	AA	AA	1	1	1	1	1	1
1025	CH10MM21BLK4	10	25210608	X	BB	AA	AA	AA	AA	AA	AA	AA	1	1	1	1	1	1
1384	CH10MM22GM3	10	25226069		BB	BB	BB	BB	BB	BB	BB	BB	1	1	1	1	1	1
647	CH10MM23	10	25713665		BB	BB	BB	BB	BB	BB	BB	BB	1	1	1	1	1	1
304	CH10MM24	10	26575945		BB	BB	BB	BB	BB	BB	BB	BB	1	1	1	1	1	1
648	CH10MM25	10	27924915		AA	AA	AA	AA	AA	AA	AA	AA	1	1	1	1	1	1
1952	CH10MM26	10	28239358		BB	BB	BB	BB	BB	BB	BB	BB	1	1	1	1	1	1
191	CH10MM27	10	28746498		AA	AA	AA	AA	AA	AA	AA	AA	1	1	1	1	1	1
649	CH10MM28	10	30500013		BB	BB	BB	BB	BB	BB	BB	BB	1	1	1	1	1	1
650	CH10MM29GM4	10	30794652		AA	AA	AA	AA	AA	AA	AA	AA	1	1	1	1	1	1
651	CH10MM30	10	34410603		BB	BB	BB	BB	BB	BB	BB	BB	1	1	1	1	1	1
652	CH10MM31	10	36058713		AA	AA	AA	AA	AA	AA	AA	AA	1	1	1	1	1	1
653	CH10MM32	10	38833025		BB	BB	BB	BB	BB	BB	BB	BB	1	1	1	1	1	1
1453	CH10MM33	10	42609040		AA	AA	AA	AA	AA	AA	AA	AA	1	1	1	1	1	1
654	CH10MM34	10	44227379		BB	BB	BB	BB	BB	BB	BB	BB	1	1	1	1	1	1
34	CH10MM35	10	46671493		AA	AA	AA	AA	AA	AA	AA	AA	1	1	1	1	1	1
1215	CH10MM36	10	46923056		BB	BB	BB	BB	BB	BB	BB	BB	1	1	1	1	1	1
1005	CH10MM37	10	48216244		BB	BB	BB	BB	BB	BB	BB	BB	1	1	1	1	1	1
1934	CH10MM38	10	48683209		AA	AA	AA	AA	AA	AA	AA	AA	1	1	1	1	1	1
192	CH10MM39	10	50989663		AA	AA	AA	AA	AA	AA	AA	AA	1	1	1	1	1	1
1316	CH10MM40	10	53783742		AA	AA	AA	AA	AA	AA	AA	AA	1	1	1	1	1	1
1728	CH10MM41	10	55832573		AA	AA	AA	AA	AA	AA	AA	AA	1	1	1	1	1	1
1697	CH10MM42	10	57565714		BB	BB	BB	BB	BB	BB	BB	BB	1	1	1	1	1	1
1923	CH10MM43	10	60860895		BB	BB	BB	BB	BB	BB	BB	BB	1	1	1	1	1	1
1907	CH10MM44	10	64294902		AA	AA	AA	AA	AA	AA	AA	AA	1	1	1	1	1	1
1494	CH10MM45	10	66179274		BB	BB	BB	BB	BB	BB	BB	BB	1	1	1	1	1	1
655	CH10MM46	10	67595648		BB	BB	BB	BB	BB	BB	BB	BB	1	1	1	1	1	1
1247	CH10MM47	10	67966008		BB	BB	BB	BB	BB	BB	BB	BB	1	1	1	1	1	1
656	CH10MM48	10	69220086		AA	AA	AA	AA	AA	AA	AA	AA	1	1	1	1	1	1
1244	CH10MM49	10	69818779		AA	AA	AA	AA	AA	AA	AA	AA	1	1	1	1	1	1
1018	CH10MM50	10	71183811		BB	BB	BB	BB	BB	BB	BB	BB	1	1	1	1	1	1
35	CH10MM51	10	71736974		BB	BB	BB	BB	BB	BB	BB	BB	1	1	1	1	1	1
657	CH10MM52	10	74437771		BB	BB	BB	BB	BB	BB	BB	BB	1	1	1	1	1	1
658	CH10MM53	10	76584143		AA	AA	AA	AA	AA	AA	AA	AA	1	1	1	1	1	1
15	CH10MM54BLK11	10	80198494	X	BB	AA	AA	AA	AA	AA	AA	AA	1	1	1	1	1	1
659	CH10MM55	10	80556929		BB	BB	BB	BB	BB	BB	BB	BB	1	1	1	1	1	1
1456	CH10MM56	10	82649121		AA	AA	AA	AA	AA	AA	AA	AA	1	1	1	1	1	1
660	CH10MM57	10	84352470		BB	BB	BB	BB	BB	BB	BB	BB	1	1	1	1	1	1
1236	CH10MM58	10	85007769		BB	BB	BB	BB	BB	BB	BB	BB	1	1	1	1	1	1
661	CH10MM59	10	85606566		AA	AA	AA	AA	AA	AA	AA	AA	1	1	1	1	1	1
2012	CH10MM60	10	86034834		BB	BB	BB	BB	BB	BB	BB	BB	1	1	1	1	1	1
36	CH10MM61	10	86322095		BB	BB	BB	BB	BB	BB	BB	BB	1	1	1	1	1	1
1053	CH10MM62	10	89439033		BB	BB	BB	BB	BB	BB	BB	BB	1	1	1	1	1	1
999	CH10MM63	10	90138346		BB	BB	BB	BB	BB	BB	BB	BB	1	1	1	1	1	1
662	CH10MM64	10	90830897		BB	BB	BB	BB	BB	BB	BB	BB	1	1	1	1	1	1
193	CH10MM65	10	92745604		AA	AA	AA	AA	AA	AA	AA	AA	1	1	1	1	1	1
663	CH10MM66	10	93288075		BB	BB	BB	BB	BB	BB	BB	BB	1	1	1	1	1	1
2013	CH10MM67	10	94146810		BB	BB	BB	BB	BB	BB	BB	BB	1	1	1	1	1	1
664	CH10MM68	10	94290709		AA	AA	AA	AA	AA	AA	AA	AA	1	1	1	1	1	1
665	CH10MM69	10	97074107		BB	BB	BB	BB	BB	BB	BB	BB	1	1	1	1	1	1
666	CH10MM70	10	97430180		AA	AA	AA	AA	AA	AA	AA	AA	1	1	1	1	1	1
1675	CH10MM71	10	98541661		AA	AA	AA	AA	AA	AA	AA	AA	1	1	1	1	1	1
1718	CH10MM72	10	98639384		AA	AA	AA	AA	AA	AA	AA	AA	1	1	1	1	1	1
1253	CH10MM73	10	99061427		BB	BB	BB	BB	BB	BB	BB	BB	1	1	1	1	1	1
1739	CH10MM74	10	99352909		AA	AA	AA	AA	AA	AA	AA	AA	1	1	1	1	1	1
1410	CH10MM75	10	99892920		AA	AA	AA	AA	AA	AA	AA	AA	1	1	1	1	1	1
667	CH10MM76	10	102081906		AA	AA	AA	AA	AA	AA	AA	AA	1	1	1	1	1	1
668	CH10MM77	10	103764301		BB	BB	BB	BB	BB	BB	BB	BB	1	1	1	1	1	1
1273	CH10MM78	10	104194765		BB	BB	BB	BB	BB	BB	BB	BB	1	1	1	1	1	1
669	CH10MM79	10	105674831		AA	AA	AA	AA	AA	AA	AA	AA	1	1	1	1	1	1
1083	CH10MM80	10	106878678		BB	BB	BB	BB	BB	BB	BB	BB	1	1	1	1	1	1
1842	CH10MM81	10	107672808		AA	AA	AA	AA	AA	AA	AA	AA	1	1	1	1	1	1
1792	CH10MM82	10	108416966		AA	AA	AA	AA	AA	AA	AA	AA	1	1	1	1	1	1
1239	CH10MM83	10	114181187		BB	BB	BB	BB	BB	BB	BB	BB	1	1	1	1	1	1
670	CH10MM84	10	114518973		BB	BB	BB	BB	BB	BB	BB	BB	1	1	1	1	1	1
1746	CH10MM85	10	114650095		BB	BB	BB	BB	BB	BB	BB	BB	1	1	1	1	1	1
671	CH10MM86	10	115590088		AA	AA	AA	AA	AA	AA	AA	AA	1	1	1	1	1	1
1806	CH10MM87	10	117348939		BB	BB	BB	BB	BB	BB	BB	BB	1	1	1	1	1	1
264	CH10MM88	10	117613736		AA	AA	AA	AA	AA	AA	AA	AA	1	1	1	1	1	1
279	CH10MM89	10	119011153		BB	BB	BB	BB	BB	BB	BB	BB	1	1	1	1	1	1
31	CH10MM90	10	119602638		AA	AA	AA	AA	AA	AA	AA	AA	1	1	1	1	1	1
672	CH10MM91	10	122911418		AA	AA	AA	AA	AA	AA	AA	AA	1	1	1	1	1	1
240	CH10MM92	10	126086653		BB	BB	BB	BB	BB	BB	BB	BB	1	1	1	1	1	1
1326	CH10MM93	10	126634957		AA	AA	AA	AA	AA	AA	AA	AA	1	1	1	1	1	1
1212	CH10MM94	10	128337313		BB	BB	BB	BB	BB	BB	BB	BB	1	1	1	1	1	1
673	CH11MM1	11	3448985		BB	BB	BB	BB	BB	BB	BB	BB	1	1	1	1	1	1
674	CH11MM2	11	3781731		AA	AA	AA	AA	AA	AA	AA	AA	1	1	1	1	1	1
1246	CH11MM3	11	4575120		BB	BB	BB	BB	BB	BB	BB	BB	1	1	1	1	1	1
1595	CH11MM4	11	5679435		AA	AA	AA	AA	AA	AA	AA	AA	1	1	1	1	1	1
1729	CH11MM5	11	6306994		AA	AA	AA	AA	AA	AA	AA	AA	1	1	1	1	1	1
675	CH11MM6	11	7518795		AA	AA	AA	AA	AA	AA	AA	AA	1	1	1	1	1	1
676	CH11MM7	11	9188410		BB	BB	BB	BB	BB	BB	BB	BB	1	1	1	1	1	1
1036	CH11MM8	11	11171143		AA	AA	AA	AA	AA	AA	AA	AA	1	1	1	1	1	1
1143	CH11MM9	11	12188090		AA	AA	AA	AA	AA	AA	AA	AA	1	1	1	1	1	1
677	CH11MM10	11	12190600		AA	AA	AA	AA	AA	AA	AA	AA	1	1	1	1	1	1
1640	CH11MM11	11	13513096		BB	BB	BB	BB	BB	BB	BB	BB	1	1	1	1	1	1
37	CH11MM12	11	14992925		AA	AA	AA	AA	AA	AA	AA	AA	1	1	1	1	1	1
1861	CH11MM13	11	15647995		AA	AA	AA	AA	AA	AA	AA	AA	1	1	1	1	1	1
16	CH11MM14	11	17499495	X	BB	AA	AA	AA	AA	AA	AA	AA	1	1	1	1	1	1
1454	CH11MM15	11	17999950		BB	BB	BB	BB	BB	BB	BB	BB	1	1	1	1	1	1
194	CH11MM16	11	18007841		AA	AA	AA	AA	AA	AA	AA	AA	1	1	1	1	1	1
1500	CH11MM17	11	19364495		AA	AA	AA	AA	AA	AA	AA	AA	1	1	1	1	1	1
1991	CH11MM18	11	20321291		BB	BB	BB	BB	BB	BB	BB	BB	1	1	1	1	1	1
1037	CH11MM19	11	20848871		AA	AA	AA	AA	AA	AA	AA	AA	1	1	1	1	1	1
678	CH11MM20	11	22709634		BB	BB	BB	BB	BB	BB	BB	BB	1	1	1	1	1	1
679	CH11MM21	11	25014537		AA	AA	AA	AA	AA	AA	AA	AA	1	1	1	1	1	1
1501	CH11MM22	11	26517914		AA	AA	AA	AA	AA	AA	AA	AA	1	1	1	1	1	1
1383	CH11MM23	11	28310427		AA	AA	AA	AA	AA	AA	AA	AA	1	1	1	1	1	1
1352	CH11MM24	11	30266839		BB	BB	BB	BB	BB	BB	BB	BB	1	1	1	1	1	1
305	CH11MM25	11	30954469		AA	AA	AA	AA	AA	AA	AA	AA	1	1	1	1	1	1
1220	CH11MM26	11	32323270		BB	BB	BB	BB	BB	BB	BB	BB	1	1	1	1	1	1
1499	CH11MM27	11	32946888		AA	AA	AA	AA	AA	AA	AA	AA	1	1	1	1	1	1
1789	CH11MM28	11	34700816		AA	AA	AA	AA	AA	AA	AA	AA	1	1	1	1	1	1
1672	CH11MM29BLK3	11	35421130	X	BB	AA	AA	AA	AA	AA	AA	AA	1	1	1	1	1	1
680	CH11MM30	11	36134794		AA	AA	AA	AA	AA	AA	AA	AA	1	1	1	1	1	1
681	CH11MM31	11	37545902		BB	BB	BB	BB	BB	BB	BB	BB	1	1	1	1	1	1

1906	CH11MM32	11	38177953		AA	AA	AA	AA	AA	AA	AA	AA	1	1	1	1	1	1
1710	CH11MM33	11	39929979		AA	AA	AA	AA	AA	AA	AA	AA	1	1	1	1	1	1
1870	CH11MM34GM1	11	40270583		AA	AA	AA	AA	AA	AA	AA	AA	1	1	1	1	1	1
1360	CH11MM35	11	40967464		AA	AA	AA	AA	AA	AA	AA	AA	1	1	1	1	1	1
1630	CH11MM36	11	41212113		BB	BB	BB	BB	BB	BB	BB	BB	1	1	1	1	1	1
1082	CH11MM37	11	43847855		AA	AA	AA	AA	AA	AA	AA	AA	1	1	1	1	1	1
682	CH11MM38	11	46168151		BB	BB	BB	BB	BB	BB	BB	BB	1	1	1	1	1	1
683	CH11MM39	11	46568199		BB	BB	BB	BB	BB	BB	BB	BB	1	1	1	1	1	1
1744	CH11MM40	11	50385006		AA	AA	AA	AA	AA	AA	AA	AA	1	1	1	1	1	1
924	CH11MM41GM2	11	53423138		AA	AA	AA	AA	AA	AA	AA	AA	1	1	1	1	1	1
934	CH11MM42	11	54023122		BB	BB	BB	BB	BB	BB	BB	BB	1	1	1	1	1	1
684	CH11MM43	11	54361586		BB	BB	BB	BB	BB	BB	BB	BB	1	1	1	1	1	1
685	CH11MM44	11	54538623		AA	AA	AA	AA	AA	AA	AA	AA	1	1	1	1	1	1
1614	CH11MM45	11	55034293		AA	AA	AA	AA	AA	AA	AA	AA	1	1	1	1	1	1
1256	CH11MM46	11	56050650		BB	BB	BB	BB	BB	BB	BB	BB	1	1	1	1	1	1
1050	CH11MM47	11	56155810		AA	AA	AA	AA	AA	AA	AA	AA	1	1	1	1	1	1
686	CH11MM48	11	57856845		BB	BB	BB	BB	BB	BB	BB	BB	1	1	1	1	1	1
1328	CH11MM49	11	58180077		BB	BB	BB	BB	BB	BB	BB	BB	1	1	1	1	1	1
1635	CH11MM50	11	59099048		BB	BB	BB	BB	BB	BB	BB	BB	1	1	1	1	1	1
1418	CH11MM51	11	61065104		AA	AA	AA	AA	AA	AA	AA	AA	1	1	1	1	1	1
687	CH11MM52	11	62605165		BB	BB	BB	BB	BB	BB	BB	BB	1	1	1	1	1	1
688	CH11MM53	11	64813366		AA	AA	AA	AA	AA	AA	AA	AA	1	1	1	1	1	1
689	CH11MM54	11	66331400		AA	AA	AA	AA	AA	AA	AA	AA	1	1	1	1	1	1
690	CH11MM55	11	68383073		BB	BB	BB	BB	BB	BB	BB	BB	1	1	1	1	1	1
691	CH11MM56	11	69432934		BB	BB	BB	BB	BB	BB	BB	BB	1	1	1	1	1	1
1742	CH11MM57	11	69789936		BB	BB	BB	BB	BB	BB	BB	BB	1	1	1	1	1	1
1301	CH11MM58GM3	11	70522958		AA	AA	AA	AA	AA	AA	AA	AA	1	1	1	1	1	1
692	CH11MM59	11	72446973		BB	BB	BB	BB	BB	BB	BB	BB	1	1	1	1	1	1
1357	CH11MM60	11	72876179		AA	AA	AA	AA	AA	AA	AA	AA	1	1	1	1	1	1
693	CH11MM61	11	73722822		BB	BB	BB	BB	BB	BB	BB	BB	1	1	1	1	1	1
694	CH11MM62	11	76207708		BB	BB	BB	BB	BB	BB	BB	BB	1	1	1	1	1	1
968	CH11MM63	11	76784919		AA	AA	AA	AA	AA	AA	AA	AA	1	1	1	1	1	1
969	CH11MM64	11	76797712		BB	BB	BB	BB	BB	BB	BB	BB	1	1	1	1	1	1
967	CH11MM65	11	76809317		BB	BB	BB	BB	BB	BB	BB	BB	1	1	1	1	1	1
142	CH11MM66GM4-BM	11	78184701		BB	BB	BB	BB	BB	BB	BB	BB	1	1	1	1	1	1
17	CH11MM67BLK10	11	79068425	X	AA	BB	BB	BB	BB	BB	BB	BB	1	1	1	1	1	1
695	CH11MM68	11	79159755		BB	BB	BB	BB	BB	BB	BB	BB	1	1	1	1	1	1
1811	CH11MM69	11	82221268		AA	AA	AA	AA	AA	AA	AA	AA	1	1	1	1	1	1
696	CH11MM70	11	83014191		AA	AA	AA	AA	AA	AA	AA	AA	1	1	1	1	1	1
1125	CH11MM71	11	84334033		AA	AA	AA	AA	AA	AA	AA	AA	1	1	1	1	1	1
1088	CH11MM72	11	86362842		BB	BB	BB	BB	BB	BB	BB	BB	1	1	1	1	1	1
1475	CH11MM73	11	86560546		BB	BB	BB	BB	BB	BB	BB	BB	1	1	1	1	1	1
697	CH11MM74	11	88037356		BB	BB	BB	BB	BB	BB	BB	BB	1	1	1	1	1	1
1277	CH11MM75	11	90207658		AA	AA	AA	AA	AA	AA	AA	AA	1	1	1	1	1	1
698	CH11MM76	11	92132381		BB	BB	BB	BB	BB	BB	BB	BB	1	1	1	1	1	1
1051	CH11MM77	11	93164961		BB	BB	BB	BB	BB	BB	BB	BB	1	1	1	1	1	1
699	CH11MM78	11	95299225		AA	AA	AA	AA	AA	AA	AA	AA	1	1	1	1	1	1
1408	CH11MM79	11	96196554		AA	AA	AA	AA	AA	AA	AA	AA	1	1	1	1	1	1
700	CH11MM80	11	96275988		AA	AA	AA	AA	AA	AA	AA	AA	1	1	1	1	1	1
195	CH11MM81	11	98152651		AA	AA	AA	AA	AA	AA	AA	AA	1	1	1	1	1	1
1122	CH11MM82	11	98590564		AA	AA	AA	AA	AA	AA	AA	AA	1	1	1	1	1	1
1055	CH11MM83	11	99721952		AA	AA	AA	AA	AA	AA	AA	AA	1	1	1	1	1	1
1955	CH11MM84	11	103272243		AA	AA	AA	AA	AA	AA	AA	AA	1	1	1	1	1	1
1331	CH11MM85	11	104908478		AA	AA	AA	AA	AA	AA	AA	AA	1	1	1	1	1	1
1942	CH11MM86	11	106649550		AA	AA	AA	AA	AA	AA	AA	AA	1	1	1	1	1	1
1917	CH11MM87	11	109065922		BB	BB	BB	BB	BB	BB	BB	BB	1	1	1	1	1	1
701	CH11MM88	11	110120256		BB	BB	BB	BB	BB	BB	BB	BB	1	1	1	1	1	1
702	CH11MM89	11	110832288		AA	AA	AA	AA	AA	AA	AA	AA	1	1	1	1	1	1
1191	CH11MM90	11	111905327		AA	AA	AA	AA	AA	AA	AA	AA	1	1	1	1	1	1
1334	CH11MM91	11	113674817		AA	AA	AA	AA	AA	AA	AA	AA	1	1	1	1	1	1
703	CH11MM92	11	115572156		AA	AA	AA	AA	AA	AA	AA	AA	1	1	1	1	1	1
1430	CH11MM93	11	120738678		BB	BB	BB	BB	BB	BB	BB	BB	1	1	1	1	1	1
5	CH12MM1GM1	12	3411972		AA	AA	AA	AA	AA	AA	AA	AA	1	1	1	1	1	1
1434	CH12MM2	12	4720216		BB	BB	BB	BB	BB	BB	BB	BB	1	1	1	1	1	1
1080	CH12MM3	12	5131626		BB	BB	BB	BB	BB	BB	BB	BB	1	1	1	1	1	1
704	CH12MM4	12	5648778		AA	AA	AA	AA	AA	AA	AA	AA	1	1	1	1	1	1
1227	CH12MM5	12	7371530		BB	BB	BB	BB	BB	BB	BB	BB	1	1	1	1	1	1
1403	CH12MM6	12	9687267		AA	AA	AA	AA	AA	AA	AA	AA	1	1	1	1	1	1
1847	CH12MM7	12	10028881		AA	AA	AA	AA	AA	AA	AA	AA	1	1	1	1	1	1
705	CH12MM8	12	10691032		AA	AA	AA	AA	AA	AA	AA	AA	1	1	1	1	1	1
706	CH12MM9	12	12036755		AA	AA	AA	AA	AA	AA	AA	AA	1	1	1	1	1	1
1466	CH12MM10	12	12114801		BB	BB	BB	BB	BB	BB	BB	BB	1	1	1	1	1	1
1084	CH12MM11	12	13507742		BB	BB	BB	BB	BB	BB	BB	BB	1	1	1	1	1	1
707	CH12MM12	12	14987941		BB	BB	BB	BB	BB	BB	BB	BB	1	1	1	1	1	1
1379	CH12MM13	12	16713381		AA	AA	AA	AA	AA	AA	AA	AA	1	1	1	1	1	1
708	CH12MM14	12	16811399		BB	BB	BB	BB	BB	BB	BB	BB	1	1	1	1	1	1
709	CH12MM15	12	17701648		BB	BB	BB	BB	BB	BB	BB	BB	1	1	1	1	1	1
1458	CH12MM16	12	19689285		BB	BB	BB	BB	BB	BB	BB	BB	1	1	1	1	1	1
710	CH12MM17	12	22949944		BB	BB	BB	BB	BB	BB	BB	BB	1	1	1	1	1	1
1054	CH12MM18	12	23976362		AA	AA	AA	AA	AA	AA	AA	AA	1	1	1	1	1	1
2042	CH12MM19	12	24901541		BB	BB	BB	BB	BB	BB	BB	BB	1	1	1	1	1	1
711	CH12MM20	12	25180902		BB	BB	BB	BB	BB	BB	BB	BB	1	1	1	1	1	1
1769	CH12MM21	12	25870085		BB	BB	BB	BB	BB	BB	BB	BB	1	1	1	1	1	1
1879	CH12MM22	12	26133320		AA	AA	AA	AA	AA	AA	AA	AA	1	1	1	1	1	1
1007	CH12MM23	12	26161896		BB	BB	BB	BB	BB	BB	BB	BB	1	1	1	1	1	1
1765	CH12MM24	12	29204179		BB	BB	BB	BB	BB	BB	BB	BB	1	1	1	1	1	1
1420	CH12MM25	12	30453856		BB	BB	BB	BB	BB	BB	BB	BB	1	1	1	1	1	1
712	CH12MM26	12	30659819		BB	BB	BB	BB	BB	BB	BB	BB	1	1	1	1	1	1
1020	CH12MM27	12	31531592		AA	AA	AA	AA	AA	AA	AA	AA	1	1	1	1	1	1
1859	CH12MM28	12	32032739		AA	AA	AA	AA	AA	AA	AA	AA	1	1	1	1	1	1
1101	CH12MM29	12	33110635		AA	AA	AA	AA	AA	AA	AA	AA	1	1	1	1	1	1
713	CH12MM30	12	34850909		AA	AA	AA	AA	AA	AA	AA	AA	1	1	1	1	1	1
714	CH12MM31	12	34954150		AA	AA	AA	AA	AA	AA	AA	AA	1	1	1	1	1	1
715	CH12MM32	12	35475720		AA	AA	AA	AA	AA	AA	AA	AA	1	1	1	1	1	1
39	CH12MM33	12	38788391		AA	AA	AA	AA	AA	AA	AA	AA	1	1	1	1	1	1
1001	CH12MM34BLK4	12	38867910	X	BB	AA	AA	AA	AA	AA	AA	AA	1	1	1	1	1	1
1516	CH12MM35	12	39432172		BB	BB	BB	BB	BB	BB	BB	BB	1	1	1	1	1	1
40	CH12MM36	12	40646532		BB	BB	BB	BB	BB	BB	BB	BB	1	1	1	1	1	1
196	CH12MM37	12	43135664		BB	BB	BB	BB	BB	BB	BB	BB	1	1	1	1	1	1
716	CH12MM38	12	45537591		AA	AA	AA	AA	AA	AA	AA	AA	1	1	1	1	1	1
717	CH12MM39	12	47738222		AA	AA	AA	AA	AA	AA	AA	AA	1	1	1	1	1	1
2043	CH12MM40	12	47778721		BB	BB	BB	BB	BB	BB	BB	BB	1	1	1	1	1	1
1179	CH12MM41	12	48181013		BB	BB	BB	BB	BB	BB	BB	BB	1	1	1	1	1	1
1354	CH12MM42	12	49142988		BB	BB	BB	BB	BB	BB	BB	BB	1	1	1	1	1	1
718	CH12MM43	12	51192106		AA	AA	AA	AA	AA	AA	AA	AA	1	1	1	1	1	1
1378	CH12MM44	12	53150903		BB	BB	BB	BB	BB	BB	BB	BB	1	1	1	1	1	1
237	CH12MM45	12	54143643		BB	BB	BB	BB	BB	BB	BB	BB	1	1	1	1	1	1
719	CH12MM46	12	57054274		AA	AA	AA	AA	AA	AA	AA	AA	1	1	1	1	1	1
197	CH12MM47	12	58421210		BB	BB	BB	BB	BB	BB	BB	BB	1	1	1	1	1	1
1221	CH12MM48	12	59848542		AA	AA	AA	AA	AA	AA	AA	AA	1	1	1	1	1	1
720	CH12MM49	12	62474249		BB	BB	BB	BB	BB	BB	BB	BB	1	1	1	1	1	1
1890	CH12MM50	12	63022076		AA	AA	AA	AA	AA	AA	AA	AA	1	1	1	1	1	1
1386	CH12MM51	12	64055557		AA	AA	AA	AA	AA	AA	AA	AA	1	1	1	1	1	1
721	CH12MM52	12	64869269		BB	BB	BB	BB	BB	BB	BB	BB	1	1	1	1	1	1
1740	CH12MM53	12	65498422		AA	AA	AA	AA	AA	AA	AA	AA	1	1	1	1	1	1
722	CH12MM54	12	66729742		AA	AA	AA	AA	AA	AA	AA	AA	1	1	1	1	1	1
723	CH12MM55	12	68166409		BB	BB	BB	BB	BB	BB	BB	BB	1	1	1	1	1	1
1089	CH12MM56	12	70010856		AA	AA	AA	AA	AA	AA	AA	AA	1	1	1	1	1	1
1936	CH12MM57GM2	12	70079425		BB	BB	BB	BB	BB	BB	BB	BB	1	1	1	1	1	1
724	CH12MM58	12	71665916		AA	AA	AA	AA	AA	AA	AA	AA	1	1	1	1	1	1
198	CH12MM59	12	73900965		BB	BB	BB	BB	BB	BB	BB	BB	1	1	1	1	1	1
1133	CH12MM60	12	74558132		BB	BB	BB	BB	BB	BB	BB	BB	1	1	1	1	1	1
725	CH12MM61	12	75962840		BB	BB	BB	BB	BB	BB	BB	BB	1	1	1	1	1	1
726	CH12MM62	12	77468587		AA	AA	AA	AA	AA	AA	AA	AA	1	1	1	1	1	1
727	CH12MM63	12	79092079		AA	AA	AA	AA	AA	AA	AA	AA	1	1	1	1	1	1
1451	CH12MM64	12	82379744		AA	AA	AA	AA	AA	AA	AA	AA	1	1	1	1	1	1
296	CH12MM65	12	84315897		BB	BB	BB	BB	BB	BB	BB	BB	1	1	1	1	1	1

41	CH12MM66	12	84750094		AA	AA	AA	AA	AA	AA	AA	AA	1	1	1	1	1	1
1176	CH12MM67	12	85932990		AA	AA	AA	AA	AA	AA	AA	AA	1	1	1	1	1	1
18	CH12MM68BLK10	12	86534440	X	AA	BB	BB	BB	BB	BB	BB	BB	1	1	1	1	1	1
728	CH12MM69	12	86574038		BB	BB	BB	BB	BB	BB	BB	BB	1	1	1	1	1	1
1750	CH12MM70	12	88966851		AA	AA	AA	AA	AA	AA	AA	AA	1	1	1	1	1	1
1673	CH12MM71	12	90002245		AA	AA	AA	AA	AA	AA	AA	AA	1	1	1	1	1	1
729	CH12MM72	12	90714740		AA	AA	AA	AA	AA	AA	AA	AA	1	1	1	1	1	1
730	CH12MM73	12	92693591		AA	AA	AA	AA	AA	AA	AA	AA	1	1	1	1	1	1
731	CH12MM74	12	94195303		BB	BB	BB	BB	BB	BB	BB	BB	1	1	1	1	1	1
1449	CH12MM75	12	94501426		AA	AA	AA	AA	AA	AA	AA	AA	1	1	1	1	1	1
732	CH12MM76	12	95793494		BB	BB	BB	BB	BB	BB	BB	BB	1	1	1	1	1	1
1346	CH12MM77	12	96438694		BB	BB	BB	BB	BB	BB	BB	BB	1	1	1	1	1	1
733	CH12MM78	12	97207111		AA	AA	AA	AA	AA	AA	AA	AA	1	1	1	1	1	1
2028	CH12MM79	12	99106508		BB	BB	BB	BB	BB	BB	BB	BB	1	1	1	1	1	1
734	CH12MM80	12	101121028		AA	AA	AA	AA	AA	AA	AA	AA	1	1	1	1	1	1
1950	CH12MM81	12	103432150		AA	AA	AA	AA	AA	AA	AA	AA	1	1	1	1	1	1
2027	CH12MM82	12	104240580		BB	BB	BB	BB	BB	BB	BB	BB	1	1	1	1	1	1
38	CH12MM83	12	104545022		AA	AA	AA	AA	AA	AA	AA	AA	1	1	1	1	1	1
735	CH12MM84	12	106863515		BB	BB	BB	BB	BB	BB	BB	BB	1	1	1	1	1	1
736	CH12MM85	12	107255256		BB	BB	BB	BB	BB	BB	BB	BB	1	1	1	1	1	1
1024	CH12MM86GM3	12	108727612		BB	BB	BB	BB	BB	BB	BB	BB	1	1	1	1	1	1
1043	CH12MM87GM4	12	111163171		BB	BB	BB	BB	BB	BB	BB	BB	1	1	1	1	1	1
1292	CH12MM88	12	112704261		AA	AA	AA	AA	AA	AA	AA	AA	1	1	1	1	1	1
1882	CH12MM89	12	114227574		AA	AA	AA	AA	AA	AA	AA	AA	1	1	1	1	1	1
1759	CH13MM1	13	3556871		BB	BB	BB	BB	BB	BB	BB	BB	1	1	1	1	1	1
737	CH13MM2	13	4242635		BB	BB	BB	BB	BB	BB	BB	BB	1	1	1	1	1	1
738	CH13MM3GM1	13	5183784		BB	BB	BB	BB	BB	BB	BB	BB	1	1	1	1	1	1
739	CH13MM4	13	5374001		BB	BB	BB	BB	BB	BB	BB	BB	1	1	1	1	1	1
740	CH13MM5BLK1	13	6304055	X	BB	AA	AA	AB	AB	AA	BB	AB	1	0,5	0,5	1	0	0,5
1853	CH13MM6	13	7490090		BB	BB	BB	BB	BB	BB	BB	BB	1	1	1	1	1	1
741	CH13MM7	13	9267297		BB	BB	BB	BB	BB	BB	BB	BB	1	1	1	1	1	1
742	CH13MM8	13	10721776		AA	AA	AA	AA	AA	AA	AA	AA	1	1	1	1	1	1
743	CH13MM9	13	12961456		BB	BB	BB	BB	BB	BB	BB	BB	1	1	1	1	1	1
1892	CH13MM10	13	14769173		BB	BB	BB	BB	BB	BB	BB	BB	1	1	1	1	1	1
744	CH13MM11	13	15697964		BB	BB	BB	BB	BB	BB	BB	BB	1	1	1	1	1	1
998	CH13MM12	13	16362951		BB	BB	BB	BB	BB	BB	BB	BB	1	1	1	1	1	1
1229	CH13MM13	13	16665395		BB	BB	BB	BB	BB	BB	BB	BB	1	1	1	1	1	1
1487	CH13MM14	13	16752051		BB	BB	BB	BB	BB	BB	BB	BB	1	1	1	1	1	1
745	CH13MM15	13	18523821		AA	AA	AA	AA	AA	AA	AA	AA	1	1	1	1	1	1
1863	CH13MM16	13	19481485		AA	AA	AA	AA	AA	AA	AA	AA	1	1	1	1	1	1
1235	CH13MM17	13	20906467		BB	BB	BB	BB	BB	BB	BB	BB	1	1	1	1	1	1
2021	CH13MM18	13	21288276		AA	AA	AA	AA	AA	AA	AA	AA	1	1	1	1	1	1
1139	CH13MM19	13	22465317		AA	AA	AA	AA	AA	AA	AA	AA	1	1	1	1	1	1
2022	CH13MM20	13	23705492		BB	BB	BB	BB	BB	BB	BB	BB	1	1	1	1	1	1
1824	CH13MM21	13	25304682		AA	AA	AA	AA	AA	AA	AA	AA	1	1	1	1	1	1
42	CH13MM22	13	27061395		BB	BB	BB	BB	BB	BB	BB	BB	1	1	1	1	1	1
746	CH13MM23	13	30057195		BB	BB	BB	BB	BB	BB	BB	BB	1	1	1	1	1	1
1411	CH13MM24	13	30653000		AA	AA	AA	AA	AA	AA	AA	AA	1	1	1	1	1	1
1419	CH13MM25	13	31374009		AA	AA	AA	AA	AA	AA	AA	AA	1	1	1	1	1	1
956	CH13MM26	13	32861685		AA	AA	AA	AA	AA	AA	AA	AA	1	1	1	1	1	1
1388	CH13MM27	13	33614779		BB	BB	BB	BB	BB	BB	BB	BB	1	1	1	1	1	1
747	CH13MM28GM2	13	34222242		AA	AA	AA	AA	AA	AA	AA	AA	1	1	1	1	1	1
1509	CH13MM29	13	34496200		BB	BB	BB	BB	BB	BB	BB	BB	1	1	1	1	1	1
748	CH13MM30	13	35994885		AA	AA	AA	AA	AA	AA	AA	AA	1	1	1	1	1	1
1809	CH13MM31	13	36689418		AA	AA	AA	AA	AA	AA	AA	AA	1	1	1	1	1	1
199	CH13MM32	13	36959616		BB	BB	BB	BB	BB	BB	BB	BB	1	1	1	1	1	1
200	CH13MM33	13	39478973		BB	BB	BB	BB	BB	BB	BB	BB	1	1	1	1	1	1
1004	CH13MM34	13	41494375		AA	AA	AA	AA	AA	AA	AA	AA	1	1	1	1	1	1
749	CH13MM35	13	42287337		BB	BB	BB	BB	BB	BB	BB	BB	1	1	1	1	1	1
1431	CH13MM36	13	42620079		AA	AA	AA	AA	AA	AA	AA	AA	1	1	1	1	1	1
23	CH13MM37GM3	13	43126831		AA	AA	AA	AA	AA	AA	AA	AA	1	1	1	1	1	1
1272	CH13MM38	13	44823434		AA	AA	AA	AA	AA	AA	AA	AA	1	1	1	1	1	1
1980	CH13MM39	13	46624183		BB	BB	BB	BB	BB	BB	BB	BB	1	1	1	1	1	1
1793	CH13MM40	13	47310947		AA	AA	AA	AA	AA	AA	AA	AA	1	1	1	1	1	1
750	CH13MM41	13	50702277		BB	BB	BB	BB	BB	BB	BB	BB	1	1	1	1	1	1
1299	CH13MM42	13	54042656		BB	BB	BB	BB	BB	BB	BB	BB	1	1	1	1	1	1
1479	CH13MM43	13	55330594		BB	BB	BB	BB	BB	BB	BB	BB	1	1	1	1	1	1
1351	CH13MM44	13	56358945		AA	AA	AA	AA	AA	AA	AA	AA	1	1	1	1	1	1
201	CH13MM45	13	57848844		AA	AA	AA	AA	AA	AA	AA	AA	1	1	1	1	1	1
202	CH13MM46	13	58589465		AA	AA	AA	AA	AA	AA	AA	AA	1	1	1	1	1	1
751	CH13MM47	13	59560600		BB	BB	BB	BB	BB	BB	BB	BB	1	1	1	1	1	1
1285	CH13MM48	13	59860578		AA	AA	AA	AA	AA	AA	AA	AA	1	1	1	1	1	1
1425	CH13MM49	13	63566794		AA	AA	AA	AA	AA	AA	AA	AA	1	1	1	1	1	1
1615	CH13MM50	13	63888326		BB	BB	BB	BB	BB	BB	BB	BB	1	1	1	1	1	1
1463	CH13MM51	13	65760534		BB	BB	BB	BB	BB	BB	BB	BB	1	1	1	1	1	1
752	CH13MM52	13	67736821		AA	AA	AA	AA	AA	AA	AA	AA	1	1	1	1	1	1
753	CH13MM53	13	70810674		AA	AA	AA	AA	AA	AA	AA	AA	1	1	1	1	1	1
754	CH13MM54	13	71955117		AA	AA	AA	AA	AA	AA	AA	AA	1	1	1	1	1	1
755	CH13MM55	13	74156236		BB	BB	BB	BB	BB	BB	BB	BB	1	1	1	1	1	1
2023	CH13MM56	13	75132382		AA	AA	AA	AA	AA	AA	AA	AA	1	1	1	1	1	1
203	CH13MM57	13	77019901		AA	AA	AA	AA	AA	AA	AA	AA	1	1	1	1	1	1
756	CH13MM58	13	78683446		BB	BB	BB	BB	BB	BB	BB	BB	1	1	1	1	1	1
268	CH13MM59	13	81332738		BB	BB	BB	BB	BB	BB	BB	BB	1	1	1	1	1	1
1795	CH13MM60	13	82197498		BB	BB	BB	BB	BB	BB	BB	BB	1	1	1	1	1	1
1265	CH13MM61	13	83174585		BB	BB	BB	BB	BB	BB	BB	BB	1	1	1	1	1	1
204	CH13MM62	13	84796434		BB	BB	BB	BB	BB	BB	BB	BB	1	1	1	1	1	1
1085	CH13MM63	13	86241392		BB	BB	BB	BB	BB	BB	BB	BB	1	1	1	1	1	1
1920	CH13MM64	13	87331201		BB	BB	BB	BB	BB	BB	BB	BB	1	1	1	1	1	1
1840	CH13MM65	13	87807463		AA	AA	AA	AA	AA	AA	AA	AA	1	1	1	1	1	1
205	CH13MM66	13	88548023		BB	BB	BB	BB	BB	BB	BB	BB	1	1	1	1	1	1
1865	CH13MM67	13	88817196		BB	BB	BB	BB	BB	BB	BB	BB	1	1	1	1	1	1
206	CH13MM68	13	89447298		BB	BB	BB	BB	BB	BB	BB	BB	1	1	1	1	1	1
757	CH13MM69GM4	13	89726949		BB	BB	BB	BB	BB	BB	BB	BB	1	1	1	1	1	1
758	CH13MM70	13	94000606		BB	BB	BB	BB	BB	BB	BB	BB	1	1	1	1	1	1
1774	CH13MM71GM5	13	95528816		BB	BB	BB	BB	BB	BB	BB	BB	1	1	1	1	1	1
759	CH13MM72	13	95596507		BB	BB	BB	BB	BB	BB	BB	BB	1	1	1	1	1	1
1616	CH13MM73	13	97200857		AA	AA	AA	AA	AA	AA	AA	AA	1	1	1	1	1	1
760	CH13MM74	13	100181409		AA	AA	AA	AA	AA	AA	AA	AA	1	1	1	1	1	1
1948	CH13MM75	13	100595006		BB	BB	BB	BB	BB	BB	BB	BB	1	1	1	1	1	1
761	CH13MM76	13	102442609		AA	AA	AA	AA	AA	AA	AA	AA	1	1	1	1	1	1
762	CH13MM77	13	103563881		BB	BB	BB	BB	BB	BB	BB	BB	1	1	1	1	1	1
763	CH13MM78	13	105593959		BB	BB	BB	BB	BB	BB	BB	BB	1	1	1	1	1	1
764	CH13MM79	13	107432985		AA	AA	AA	AA	AA	AA	AA	AA	1	1	1	1	1	1
1026	CH13MM80BLK13	13	108979531	X	BB	AA	AA	AA	AA	AA	AA	AA	1	1	1	1	1	1
765	CH13MM81	13	109707882		AA	AA	AA	AA	AA	AA	AA	AA	1	1	1	1	1	1
207	CH13MM82	13	110861665		AA	AA	AA	AA	AA	AA	AA	AA	1	1	1	1	1	1
1798	CH13MM83	13	112118515		AA	AA	AA	AA	AA	AA	AA	AA	1	1	1	1	1	1
1866	CH13MM84	13	112888923		BB	BB	BB	BB	BB	BB	BB	BB	1	1	1	1	1	1
766	CH13MM85	13	113209165		AA	AA	AA	AA	AA	AA	AA	AA	1	1	1	1	1	1
767	CH13MM86	13	115056838		BB	BB	BB	BB	BB	BB	BB	BB	1	1	1	1	1	1
287	CH13MM87BLK14GM6-BM	13	120164258	X	NC	BB	BB	BB	BB	BB	BB	BB	1	1	1	1	1	1
288	CH13MM88BLK15GM7-BM	13	120164565	X	BB	NC	NC	NC	NC	NC	NC	NC	NA	NA	NA	NA	NA	NA
1617	CH14MM1	14	3890426		AA	AA	AA	AA	AA	AA	AA	AA	1	1	1	1	1	1
768	CH14MM2GM1	14	4816550		AA	AA	AA	AA	AA	AA	AA	AA	1	1	1	1	1	1
1875	CH14MM3	14	5945767		AA	AA	AA	AA	AA	AA	AA	AA	1	1	1	1	1	1
1061	CH14MM4	14	7401248		BB	BB	BB	BB	BB	BB	BB	BB	1	1	1	1	1	1
1190	CH14MM5	14	7512933		AA	AA	AA	AA	AA	AA	AA	AA	1	1	1	1	1	1
1687	CH14MM6	14	8435084		AA	AA	AA	AA	AA	AA	AA	AA	1	1	1	1	1	1
769	CH14MM7	14	8848582		BB	BB	BB	BB	BB	BB	BB	BB	1	1	1	1	1	1
1844	CH14MM8	14	9149500		AA	AA	AA	AA	AA	AA	AA	AA	1	1	1	1	1	1
1476	CH14MM9	14	12874743		BB	BB	BB	BB	BB	BB	BB	BB	1	1	1	1	1	1
1270	CH14MM10	14	15973497		AA	AA	AA	AA	AA	AA	AA	AA	1	1	1	1	1	1
1317	CH14MM11	14	17362990		AA	AA	AA	AA	AA	AA	AA	AA	1	1	1	1	1	1
770	CH14MM12	14	19006267		BB	BB	BB	BB	BB	BB	BB	BB	1	1	1	1	1	1
1291	CH14MM13	14	20092583		AA	AA	AA	AA	AA	AA	AA	AA	1	1	1	1	1	1
208	CH14MM14	14	21555237		AA	AA	AA	AA	AA	AA	AA	AA	1	1	1	1	1	1
1649	CH14MM15	14	22146531		BB	BB	BB	BB	BB	BB	BB	BB	1	1	1	1	1	1

771	CH14MM16	14	22364262		AA	AA	AA	AA	AA	AA	AA	AA	1	1	1	1	1	1
1959	CH14MM17	14	24042337		BB	BB	BB	BB	BB	BB	BB	BB	1	1	1	1	1	1
772	CH14MM18	14	25516466		BB	BB	BB	BB	BB	BB	BB	BB	1	1	1	1	1	1
1618	CH14MM19	14	27218513		AA	AA	AA	AA	AA	AA	AA	AA	1	1	1	1	1	1
773	CH14MM20	14	27701835		AA	AA	AA	AA	AA	AA	AA	AA	1	1	1	1	1	1
1862	CH14MM21	14	28306368		AA	AA	AA	AA	AA	AA	AA	AA	1	1	1	1	1	1
1307	CH14MM22	14	29838823		BB	BB	BB	BB	BB	BB	BB	BB	1	1	1	1	1	1
774	CH14MM23	14	31429298		AA	AA	AA	AA	AA	AA	AA	AA	1	1	1	1	1	1
1681	CH14MM24	14	31484473		BB	BB	BB	BB	BB	BB	BB	BB	1	1	1	1	1	1
1361	CH14MM25	14	33391532		BB	BB	BB	BB	BB	BB	BB	BB	1	1	1	1	1	1
775	CH14MM26	14	35194216		AA	AA	AA	AA	AA	AA	AA	AA	1	1	1	1	1	1
776	CH14MM27	14	35609633		BB	BB	BB	BB	BB	BB	BB	BB	1	1	1	1	1	1
777	CH14MM28	14	37242020		BB	BB	BB	BB	BB	BB	BB	BB	1	1	1	1	1	1
778	CH14MM29	14	38359897		BB	BB	BB	BB	BB	BB	BB	BB	1	1	1	1	1	1
209	CH14MM30	14	39508866		AA	AA	AA	AA	AA	AA	AA	AA	1	1	1	1	1	1
779	CH14MM31	14	41201261		AA	AA	AA	AA	AA	AA	AA	AA	1	1	1	1	1	1
269	CH14MM32	14	42906842		BB	BB	BB	BB	BB	BB	BB	BB	1	1	1	1	1	1
1161	CH14MM33	14	44880408		BB	BB	BB	BB	BB	BB	BB	BB	1	1	1	1	1	1
1984	CH14MM34	14	46391975		BB	BB	BB	BB	BB	BB	BB	BB	1	1	1	1	1	1
1685	CH14MM35	14	46613044		AA	AA	AA	AA	AA	AA	AA	AA	1	1	1	1	1	1
780	CH14MM36	14	46619173		BB	BB	BB	BB	BB	BB	BB	BB	1	1	1	1	1	1
781	CH14MM37	14	47596815		BB	BB	BB	BB	BB	BB	BB	BB	1	1	1	1	1	1
782	CH14MM38	14	47801247		AA	AA	AA	AA	AA	AA	AA	AA	1	1	1	1	1	1
1295	CH14MM39	14	50807707		AA	AA	AA	AA	AA	AA	AA	AA	1	1	1	1	1	1
210	CH14MM40	14	52829293		AA	AA	AA	AA	AA	AA	AA	AA	1	1	1	1	1	1
783	CH14MM41	14	53885592		BB	BB	BB	BB	BB	BB	BB	BB	1	1	1	1	1	1
1634	CH14MM42	14	54793166		AA	AA	AA	AA	AA	AA	AA	AA	1	1	1	1	1	1
1364	CH14MM43	14	54913181		BB	BB	BB	BB	BB	BB	BB	BB	1	1	1	1	1	1
784	CH14MM44	14	58969551		BB	BB	BB	BB	BB	BB	BB	BB	1	1	1	1	1	1
1189	CH14MM45	14	60335045		AA	AA	AA	AA	AA	AA	AA	AA	1	1	1	1	1	1
785	CH14MM46	14	61543475		BB	BB	BB	BB	BB	BB	BB	BB	1	1	1	1	1	1
1619	CH14MM47GM2	14	62380282		AA	AA	AA	AA	AA	AA	AA	AA	1	1	1	1	1	1
1495	CH14MM48	14	63041573		AA	AA	AA	AA	AA	AA	AA	AA	1	1	1	1	1	1
786	CH14MM49	14	63674408		AA	AA	AA	AA	AA	AA	AA	AA	1	1	1	1	1	1
1358	CH14MM50	14	64968413		AA	AA	AA	AA	AA	AA	AA	AA	1	1	1	1	1	1
1309	CH14MM51	14	65580751		BB	BB	BB	BB	BB	BB	BB	BB	1	1	1	1	1	1
1701	CH14MM52	14	66473686		AA	AA	AA	AA	AA	AA	AA	AA	1	1	1	1	1	1
306	CH14MM53	14	67681669		BB	BB	BB	BB	BB	BB	BB	BB	1	1	1	1	1	1
787	CH14MM54	14	68180077		AA	AA	AA	AA	AA	AA	AA	AA	1	1	1	1	1	1
788	CH14MM55	14	70793464		BB	BB	BB	BB	BB	BB	BB	BB	1	1	1	1	1	1
1038	CH14MM56	14	71540901		AA	AA	AA	AA	AA	AA	AA	AA	1	1	1	1	1	1
1683	CH14MM57BLK4	14	72408389	X	AA	BB	BB	BB	BB	BB	BB	BB	1	1	1	1	1	1
43	CH14MM58	14	73162771		BB	BB	BB	BB	BB	BB	BB	BB	1	1	1	1	1	1
789	CH14MM59	14	74092548		BB	BB	BB	BB	BB	BB	BB	BB	1	1	1	1	1	1
1871	CH14MM60	14	75261976		AA	AA	AA	AA	AA	AA	AA	AA	1	1	1	1	1	1
952	CH14MM61	14	75264080		BB	BB	BB	BB	BB	BB	BB	BB	1	1	1	1	1	1
790	CH14MM62	14	78061668		BB	BB	BB	BB	BB	BB	BB	BB	1	1	1	1	1	1
1401	CH14MM63	14	78686463		BB	BB	BB	BB	BB	BB	BB	BB	1	1	1	1	1	1
791	CH14MM64	14	80784363		BB	BB	BB	BB	BB	BB	BB	BB	1	1	1	1	1	1
1958	CH14MM65	14	82944063		BB	BB	BB	BB	BB	BB	BB	BB	1	1	1	1	1	1
1845	CH14MM66	14	83887230		BB	BB	BB	BB	BB	BB	BB	BB	1	1	1	1	1	1
792	CH14MM67	14	85287050		AA	AA	AA	AA	AA	AA	AA	AA	1	1	1	1	1	1
793	CH14MM68	14	89372338		BB	BB	BB	BB	BB	BB	BB	BB	1	1	1	1	1	1
1293	CH14MM69	14	92002849		BB	BB	BB	BB	BB	BB	BB	BB	1	1	1	1	1	1
1731	CH14MM70	14	92434091		BB	BB	BB	BB	BB	BB	BB	BB	1	1	1	1	1	1
44	CH14MM71	14	94845626		AA	AA	AA	AA	AA	AA	AA	AA	1	1	1	1	1	1
794	CH14MM72	14	98188914		AA	AA	AA	AA	AA	AA	AA	AA	1	1	1	1	1	1
1081	CH14MM73	14	98578208		AA	AA	AA	AA	AA	AA	AA	AA	1	1	1	1	1	1
1226	CH14MM74	14	98713773		AA	AA	AA	AA	AA	AA	AA	AA	1	1	1	1	1	1
795	CH14MM75	14	99047545		BB	BB	BB	BB	BB	BB	BB	BB	1	1	1	1	1	1
211	CH14MM76	14	99774290		BB	BB	BB	BB	BB	BB	BB	BB	1	1	1	1	1	1
1633	CH14MM77BLK8	14	102368939	X	BB	AA	AA	AA	AA	AA	AA	AA	1	1	1	1	1	1
1402	CH14MM78	14	103068000		AA	AA	AA	AA	AA	AA	AA	AA	1	1	1	1	1	1
1620	CH14MM79GM3	14	103363409		BB	BB	BB	BB	BB	BB	BB	BB	1	1	1	1	1	1
796	CH14MM80	14	103981340		AA	AA	AA	AA	AA	AA	AA	AA	1	1	1	1	1	1
1515	CH14MM81	14	104244696		BB	BB	BB	BB	BB	BB	BB	BB	1	1	1	1	1	1
1804	CH14MM82	14	106108556		BB	BB	BB	BB	BB	BB	BB	BB	1	1	1	1	1	1
1713	CH14MM83	14	109578622		AA	AA	AA	AA	AA	AA	AA	AA	1	1	1	1	1	1
797	CH14MM84	14	110900259		BB	BB	BB	BB	BB	BB	BB	BB	1	1	1	1	1	1
1157	CH14MM85	14	111354233		AA	AA	AA	AA	AA	AA	AA	AA	1	1	1	1	1	1
798	CH14MM86	14	112310948		BB	BB	BB	BB	BB	BB	BB	BB	1	1	1	1	1	1
1332	CH14MM87	14	113996232		BB	BB	BB	BB	BB	BB	BB	BB	1	1	1	1	1	1
799	CH14MM88	14	114388484		AA	AA	AA	AA	AA	AA	AA	AA	1	1	1	1	1	1
311	CH15MM1GM1	15	3104758		BB	BB	BB	BB	BB	BB	BB	BB	1	1	1	1	1	1
1679	CH15MM2	15	3345023		AA	AA	AA	AA	AA	AA	AA	AA	1	1	1	1	1	1
800	CH15MM3	15	3498960		AA	AA	AA	AA	AA	AA	AA	AA	1	1	1	1	1	1
312	CH15MM4	15	3802916		AA	AA	AA	AA	AA	AA	AA	AA	1	1	1	1	1	1
49	CH15MM5	15	4222769		BB	BB	BB	BB	BB	BB	BB	BB	1	1	1	1	1	1
1158	CH15MM6	15	5744460		BB	BB	BB	BB	BB	BB	BB	BB	1	1	1	1	1	1
51	CH15MM7	15	8331158		AA	AA	AA	AA	AA	AA	AA	AA	1	1	1	1	1	1
52	CH15MM8	15	9687257		AA	AA	AA	AA	AA	AA	AA	AA	1	1	1	1	1	1
801	CH15MM9	15	10037310		AA	AA	AA	AA	AA	AA	AA	AA	1	1	1	1	1	1
802	CH15MM10	15	11218285		AA	AA	AA	AA	AA	AA	AA	AA	1	1	1	1	1	1
1014	CH15MM11BLK2	15	11336383	X	AA	BB	BB	BB	BB	BB	BB	BB	1	1	1	1	1	1
19	CH15MM12	15	11377555	X	AA	BB	BB	BB	BB	BB	BB	BB	1	1	1	1	1	1
1424	CH15MM13	15	13122871		AA	AA	AA	AA	AA	AA	AA	AA	1	1	1	1	1	1
212	CH15MM14	15	14296154		BB	BB	BB	BB	BB	BB	BB	BB	1	1	1	1	1	1
45	CH15MM15	15	14786403		AA	AA	AA	AA	AA	AA	AA	AA	1	1	1	1	1	1
46	CH15MM16	15	15482356		BB	BB	BB	BB	BB	BB	BB	BB	1	1	1	1	1	1
47	CH15MM17	15	16073911		AA	AA	AA	AA	AA	AA	AA	AA	1	1	1	1	1	1
803	CH15MM18	15	16643375		BB	BB	BB	BB	BB	BB	BB	BB	1	1	1	1	1	1
1447	CH15MM19	15	19261351		BB	BB	BB	BB	BB	BB	BB	BB	1	1	1	1	1	1
804	CH15MM20	15	20978602		AA	AA	AA	AA	AA	AA	AA	AA	1	1	1	1	1	1
805	CH15MM21	15	22545113		BB	BB	BB	BB	BB	BB	BB	BB	1	1	1	1	1	1
806	CH15MM22	15	24640823		AA	AA	AA	AA	AA	AA	AA	AA	1	1	1	1	1	1
807	CH15MM23	15	25491892		BB	BB	BB	BB	BB	BB	BB	BB	1	1	1	1	1	1
1132	CH15MM24	15	26094714		BB	BB	BB	BB	BB	BB	BB	BB	1	1	1	1	1	1
808	CH15MM25	15	28363926		AA	AA	AA	AA	AA	AA	AA	AA	1	1	1	1	1	1
809	CH15MM26	15	29311563		BB	BB	BB	BB	BB	BB	BB	BB	1	1	1	1	1	1
810	CH15MM27	15	30361234		AA	AA	AA	AA	AA	AA	AA	AA	1	1	1	1	1	1
1162	CH15MM28	15	30800680		AA	AA	AA	AA	AA	AA	AA	AA	1	1	1	1	1	1
811	CH15MM29GM2	15	31491239		AA	AA	AA	AA	AA	AA	AA	AA	1	1	1	1	1	1
812	CH15MM30	15	32096277		AA	AA	AA	AA	AA	AA	AA	AA	1	1	1	1	1	1
1727	CH15MM31	15	34383985		AA	AA	AA	AA	AA	AA	AA	AA	1	1	1	1	1	1
48	CH15MM32	15	36490596		BB	BB	BB	BB	BB	BB	BB	BB	1	1	1	1	1	1
1308	CH15MM33	15	38562542		AA	AA	AA	AA	AA	AA	AA	AA	1	1	1	1	1	1
813	CH15MM34	15	39096937		BB	BB	BB	BB	BB	BB	BB	BB	1	1	1	1	1	1
1248	CH15MM35	15	41359817		BB	BB	BB	BB	BB	BB	BB	BB	1	1	1	1	1	1
50	CH15MM36	15	43206205		AA	AA	AA	AA	AA	AA	AA	AA	1	1	1	1	1	1
814	CH15MM37	15	43892080		BB	BB	BB	BB	BB	BB	BB	BB	1	1	1	1	1	1
1201	CH15MM38	15	44244940		AA	AA	AA	AA	AA	AA	AA	AA	1	1	1	1	1	1
815	CH15MM39	15	45665306		AA	AA	AA	AA	AA	AA	AA	AA	1	1	1	1	1	1
816	CH15MM40	15	47188758		AA	AA	AA	AA	AA	AA	AA	AA	1	1	1	1	1	1
817	CH15MM41	15	50021404		BB	BB	BB	BB	BB	BB	BB	BB	1	1	1	1	1	1
1105	CH15MM42	15	51403508		AA	AA	AA	AA	AA	AA	AA	AA	1	1	1	1	1	1
1392	CH15MM43	15	52526197		AA	AA	AA	AA	AA	AA	AA	AA	1	1	1	1	1	1
818	CH15MM44	15	53062977		BB	BB	BB	BB	BB	BB	BB	BB	1	1	1	1	1	1
1658	CH15MM45	15	53498153		BB	BB	BB	BB	BB	BB	BB	BB	1	1	1	1	1	1
819	CH15MM46	15	54079758		BB	BB	BB	BB	BB	BB	BB	BB	1	1	1	1	1	1
20	CH15MM47BLK7	15	56990569	X	BB	AA	BB	AA	BB	BB	BB	BB	0	1	0	0	0	0
1964	CH15MM48	15	56998500		AA	AA	AA	AA	AA	AA	AA	AA	1	1	1	1	1	1
820	CH15MM49	15	58814425		BB	BB	BB	BB	BB	BB	BB	BB	1	1	1	1	1	1
1250	CH15MM50	15	61406689		BB	BB	BB	BB	BB	BB	BB	BB	1	1	1	1	1	1
294	CH15MM51	15	61573448		BB	BB	BB	BB	BB	BB	BB	BB	1	1	1	1	1	1
821	CH15MM52	15	61631955		BB	BB	BB	BB	BB	BB	BB	BB	1	1	1	1	1	1
1218	CH15MM53	15	63677792		BB	BB	BB	BB	BB	BB	BB	BB	1	1	1	1	1	1
1932	CH15MM54	15	65856370		AA	AA	AA	AA	AA	AA	AA	AA	1	1	1	1	1	1

822	CH15MM55	15	68143270		AA	AA	AA	AA	AA	AA	AA	AA	1	1	1	1	1	1
823	CH15MM56	15	68448503		AA	AA	AA	AA	AA	AA	AA	AA	1	1	1	1	1	1
824	CH15MM57	15	70770335		AA	AA	AA	AA	AA	AA	AA	AA	1	1	1	1	1	1
1338	CH15MM58	15	71605742		BB	BB	BB	BB	BB	BB	BB	BB	1	1	1	1	1	1
825	CH15MM59	15	72189223		BB	BB	BB	BB	BB	BB	BB	BB	1	1	1	1	1	1
826	CH15MM60	15	72483350		BB	BB	BB	BB	BB	BB	BB	BB	1	1	1	1	1	1
2015	CH15MM61	15	75141907		BB	BB	BB	BB	BB	BB	BB	BB	1	1	1	1	1	1
1116	CH15MM62	15	78151819		BB	BB	BB	BB	BB	BB	BB	BB	1	1	1	1	1	1
1355	CH15MM63	15	78678294		AA	AA	AA	AA	AA	AA	AA	AA	1	1	1	1	1	1
827	CH15MM64	15	79085767		BB	BB	BB	BB	BB	BB	BB	BB	1	1	1	1	1	1
1154	CH15MM65	15	79719777		AA	AA	AA	AA	AA	AA	AA	AA	1	1	1	1	1	1
1826	CH15MM66	15	82164497		AA	AA	AA	AA	AA	AA	AA	AA	1	1	1	1	1	1
1990	CH15MM67	15	82403644		BB	BB	BB	BB	BB	BB	BB	BB	1	1	1	1	1	1
828	CH15MM68	15	82560799		BB	BB	BB	BB	BB	BB	BB	BB	1	1	1	1	1	1
1118	CH15MM69	15	85057715		AA	AA	AA	AA	AA	AA	AA	AA	1	1	1	1	1	1
1240	CH15MM70GM3	15	85598596		AA	AA	AA	AA	AA	AA	AA	AA	1	1	1	1	1	1
829	CH15MM71	15	86303733		BB	BB	BB	BB	BB	BB	BB	BB	1	1	1	1	1	1
830	CH15MM72	15	88856958		AA	AA	AA	AA	AA	AA	AA	AA	1	1	1	1	1	1
831	CH15MM73	15	90816455		AA	AA	AA	AA	AA	AA	AA	AA	1	1	1	1	1	1
1878	CH15MM74	15	90938588		AA	AA	AA	AA	AA	AA	AA	AA	1	1	1	1	1	1
832	CH15MM75	15	92664963		AA	AA	AA	AA	AA	AA	AA	AA	1	1	1	1	1	1
1258	CH15MM76	15	93757815		AA	AA	AA	AA	AA	AA	AA	AA	1	1	1	1	1	1
1468	CH15MM77	15	95679367		AA	AA	AA	AA	AA	AA	AA	AA	1	1	1	1	1	1
1832	CH15MM78	15	95741617		AA	AA	AA	AA	AA	AA	AA	AA	1	1	1	1	1	1
833	CH15MM79	15	97550224		AA	AA	AA	AA	AA	AA	AA	AA	1	1	1	1	1	1
834	CH15MM80	15	100255519		AA	AA	AA	AA	AA	AA	AA	AA	1	1	1	1	1	1
835	CH15MM81	15	102030597		BB	BB	BB	BB	BB	BB	BB	BB	1	1	1	1	1	1
1039	CH15MM82	15	102289996		BB	BB	BB	BB	BB	BB	BB	BB	1	1	1	1	1	1
836	CH15MM83	15	103433393		BB	BB	BB	BB	BB	BB	BB	BB	1	1	1	1	1	1
1542	CH16MM1	16	4009670		BB	BB	BB	BB	BB	BB	BB	BB	1	1	1	1	1	1
1543	CH16MM2	16	4532147		BB	BB	BB	BB	BB	BB	BB	BB	1	1	1	1	1	1
1621	CH16MM3	16	5907681		AA	AA	AA	AA	AA	AA	AA	AA	1	1	1	1	1	1
6	CH16MM4GM1	16	6063341		AA	AA	AA	AA	AA	AA	AA	AA	1	1	1	1	1	1
1544	CH16MM5	16	7650712		BB	BB	BB	BB	BB	BB	BB	BB	1	1	1	1	1	1
1545	CH16MM6	16	9258154		BB	BB	BB	BB	BB	BB	BB	BB	1	1	1	1	1	1
1546	CH16MM7	16	10565999		AA	AA	AA	AA	AA	AA	AA	AA	1	1	1	1	1	1
1547	CH16MM8	16	12215630		BB	BB	BB	BB	BB	BB	BB	BB	1	1	1	1	1	1
1548	CH16MM9	16	13143895		AA	AA	AA	AA	AA	AA	AA	AA	1	1	1	1	1	1
284	CH16MM10GM2-BM	16	15839000		BB	BB	BB	BB	BB	BB	BB	BB	1	1	1	1	1	1
1989	CH16MM11	16	18411482		AA	AA	AA	AA	AA	AA	AA	AA	1	1	1	1	1	1
1549	CH16MM12	16	19654956		AA	AA	AA	AA	AA	AA	AA	AA	1	1	1	1	1	1
1550	CH16MM13	16	21155526		AA	AA	AA	AA	AA	AA	AA	AA	1	1	1	1	1	1
1551	CH16MM14	16	24022362		BB	BB	BB	BB	BB	BB	BB	BB	1	1	1	1	1	1
1552	CH16MM15	16	24347718	X	BB	AA	AA	AA	AA	AA	AA	AA	1	1	1	1	1	1
1553	CH16MM16	16	25257147		BB	BB	BB	BB	BB	BB	BB	BB	1	1	1	1	1	1
1554	CH16MM17GM3	16	26978114		AA	AA	AA	AA	AA	AA	AA	AA	1	1	1	1	1	1
1555	CH16MM18	16	27277369		BB	BB	BB	BB	BB	BB	BB	BB	1	1	1	1	1	1
239	CH16MM19	16	28698520		BB	BB	BB	BB	BB	BB	BB	BB	1	1	1	1	1	1
1556	CH16MM20	16	30855920		BB	BB	BB	BB	BB	BB	BB	BB	1	1	1	1	1	1
1665	CH16MM21BLK4	16	32279982	X	BB	AA	AA	AA	AA	AA	AA	AA	1	1	1	1	1	1
1557	CH16MM22	16	32338346		AA	AA	AA	AA	AA	AA	AA	AA	1	1	1	1	1	1
1217	CH16MM23GM4	16	32345239		AA	AA	AA	AA	AA	AA	AA	AA	1	1	1	1	1	1
1558	CH16MM24	16	34183107		AA	AA	AA	AA	AA	AA	AA	AA	1	1	1	1	1	1
1559	CH16MM25	16	35504262		AA	AA	AA	AA	AA	AA	AA	AA	1	1	1	1	1	1
1560	CH16MM26GM5	16	36434699		BB	BB	BB	BB	BB	BB	BB	BB	1	1	1	1	1	1
278	CH16MM27	16	37207323		BB	BB	BB	BB	BB	BB	BB	BB	1	1	1	1	1	1
1561	CH16MM28	16	39046740		BB	BB	BB	BB	BB	BB	BB	BB	1	1	1	1	1	1
1562	CH16MM29	16	40548715		BB	BB	BB	BB	BB	BB	BB	BB	1	1	1	1	1	1
1563	CH16MM30GM6	16	42022510		AA	AA	AA	AA	AA	AA	AA	AA	1	1	1	1	1	1
1564	CH16MM31	16	42205435		BB	BB	BB	BB	BB	BB	BB	BB	1	1	1	1	1	1
1565	CH16MM32	16	45247290		BB	BB	BB	BB	BB	BB	BB	BB	1	1	1	1	1	1
1566	CH16MM33	16	46008686		BB	BB	BB	BB	BB	BB	BB	BB	1	1	1	1	1	1
1567	CH16MM34	16	47148630		AA	AA	AA	AA	AA	AA	AA	AA	1	1	1	1	1	1
1568	CH16MM35	16	50209266		AA	AA	AA	AA	AA	AA	AA	AA	1	1	1	1	1	1
1661	CH16MM36	16	50369824		BB	BB	BB	BB	BB	BB	BB	BB	1	1	1	1	1	1
1569	CH16MM37	16	50656592		BB	BB	BB	BB	BB	BB	BB	BB	1	1	1	1	1	1
1570	CH16MM38GM7	16	50970496		BB	BB	BB	BB	BB	BB	BB	BB	1	1	1	1	1	1
238	CH16MM39	16	51371684		AA	AA	AA	AA	AA	AA	AA	AA	1	1	1	1	1	1
1319	CH16MM40	16	51606253		AA	AA	AA	AA	AA	AA	AA	AA	1	1	1	1	1	1
1709	CH16MM41	16	53434895		BB	BB	BB	BB	BB	BB	BB	BB	1	1	1	1	1	1
138	CH16MM42	16	55104521		AA	AA	AA	AA	AA	AA	AA	AA	1	1	1	1	1	1
1571	CH16MM43	16	56631777		BB	BB	BB	BB	BB	BB	BB	BB	1	1	1	1	1	1
1572	CH16MM44GM8	16	56675300		BB	BB	BB	BB	BB	BB	BB	BB	1	1	1	1	1	1
1573	CH16MM45	16	57472587		AA	AA	AA	AA	AA	AA	AA	AA	1	1	1	1	1	1
1575	CH16MM46BLK7	16	57729119	X	BB	AA	AA	AA	AA	AA	AA	AA	1	1	1	1	1	1
1574	CH16MM47	16	57732226		BB	BB	BB	BB	BB	BB	BB	BB	1	1	1	1	1	1
1576	CH16MM48	16	59391239		BB	BB	BB	BB	BB	BB	BB	BB	1	1	1	1	1	1
1577	CH16MM49	16	61234639		BB	BB	BB	BB	BB	BB	BB	BB	1	1	1	1	1	1
1578	CH16MM50	16	63055086		AA	AA	AA	AA	AA	AA	AA	AA	1	1	1	1	1	1
1682	CH16MM51	16	64902231		BB	BB	BB	BB	BB	BB	BB	BB	1	1	1	1	1	1
1579	CH16MM52	16	66573423		AA	AA	AA	AA	AA	AA	AA	AA	1	1	1	1	1	1
1580	CH16MM53	16	67541594		AA	AA	AA	AA	AA	AA	AA	AA	1	1	1	1	1	1
1581	CH16MM54	16	69301525		AA	AA	AA	AA	AA	AA	AA	AA	1	1	1	1	1	1
1582	CH16MM55	16	71033149		BB	BB	BB	BB	BB	BB	BB	BB	1	1	1	1	1	1
1583	CH16MM56	16	72860280		AA	AA	AA	AA	AA	AA	AA	AA	1	1	1	1	1	1
1484	CH16MM57	16	73110160		BB	BB	BB	BB	BB	BB	BB	BB	1	1	1	1	1	1
1584	CH16MM58	16	74547672		BB	BB	BB	BB	BB	BB	BB	BB	1	1	1	1	1	1
1585	CH16MM59	16	76732911		AA	AA	AA	AA	AA	AA	AA	AA	1	1	1	1	1	1
1459	CH16MM60	16	77227010		BB	BB	BB	BB	BB	BB	BB	BB	1	1	1	1	1	1
1586	CH16MM61	16	78490469		BB	BB	BB	BB	BB	BB	BB	BB	1	1	1	1	1	1
2025	CH16MM62	16	79034420		BB	BB	BB	BB	BB	BB	BB	BB	1	1	1	1	1	1
1869	CH16MM63	16	79873189		AA	AA	AA	AA	AA	AA	AA	AA	1	1	1	1	1	1
1587	CH16MM64	16	81210555		AA	AA	AA	AA	AA	AA	AA	AA	1	1	1	1	1	1
1588	CH16MM65	16	83161949		BB	BB	BB	BB	BB	BB	BB	BB	1	1	1	1	1	1
1666	CH16MM66	16	83781488		BB	BB	BB	BB	BB	BB	BB	BB	1	1	1	1	1	1
1238	CH16MM67	16	84938923		BB	BB	BB	BB	BB	BB	BB	BB	1	1	1	1	1	1
1589	CH16MM68	16	85479261		AA	AA	AA	AA	AA	AA	AA	AA	1	1	1	1	1	1
1590	CH16MM69	16	86836511		AA	AA	AA	AA	AA	AA	AA	AA	1	1	1	1	1	1
1591	CH16MM70	16	87532305		AA	AA	AA	AA	AA	AA	AA	AA	1	1	1	1	1	1
1592	CH16MM71	16	87920304		AA	AA	AA	AA	AA	AA	AA	AA	1	1	1	1	1	1
289	CH16MM72	16	88587815		BB	BB	BB	BB	BB	BB	BB	BB	1	1	1	1	1	1
1593	CH16MM73	16	90298369		BB	BB	BB	BB	BB	BB	BB	BB	1	1	1	1	1	1
1594	CH16MM74	16	93471889		AA	AA	AA	AA	AA	AA	AA	AA	1	1	1	1	1	1
1398	CH16MM75	16	93669455		BB	BB	BB	BB	BB	BB	BB	BB	1	1	1	1	1	1
1426	CH16MM76	16	94018736		BB	BB	BB	BB	BB	BB	BB	BB	1	1	1	1	1	1
2032	CH16MM77	16	97576033		AA	AA	AA	AA	AA	AA	AA	AA	1	1	1	1	1	1
2033	CH16MM78	16	97591035		AA	AA	AA	AA	AA	AA	AA	AA	1	1	1	1	1	1
2031	CH16MM79	16	97611851		AA	AA	AA	AA	AA	AA	AA	AA	1	1	1	1	1	1
1324	CH16MM80	16	98191753		AA	AA	AA	AA	AA	AA	AA	AA	1	1	1	1	1	1
1525	CH17MM1	17	3388654		BB	BB	BB	BB	BB	BB	BB	BB	1	1	1	1	1	1
1787	CH17MM2	17	3766890		AA	AA	AA	AA	AA	AA	AA	AA	1	1	1	1	1	1
837	CH17MM3	17	4044700		BB	BB	BB	BB	BB	BB	BB	BB	1	1	1	1	1	1
838	CH17MM4	17	4592511		AA	AA	AA	AA	AA	AA	AA	AA	1	1	1	1	1	1
1078	CH17MM5	17	4662490		BB	BB	BB	BB	BB	BB	BB	BB	1	1	1	1	1	1
2044	CH17MM6	17	5989632		BB	BB	BB	BB	BB	BB	BB	BB	1	1	1	1	1	1
1741	CH17MM7	17	8381669		AA	AA	AA	AA	AA	AA	AA	AA	1	1	1	1	1	1
1131	CH17MM8	17	9388595		AA	AA	AA	AA	AA	AA	AA	AA	1	1	1	1	1	1
1645	CH17MM9	17	11233291		BB	BB	BB	BB	BB	BB	BB	BB	1	1	1	1	1	1
1135	CH17MM10	17	11723443		BB	BB	BB	BB	BB	BB	BB	BB	1	1	1	1	1	1
307	CH17MM11	17	11992689		AA	AA	AA	AA	AA	AA	AA	AA	1	1	1	1	1	1
53	CH17MM12	17	12736614		BB	BB	BB	BB	BB	BB	BB	BB	1	1	1	1	1	1
1188	CH17MM13	17	13064499		BB	BB	BB	BB	BB	BB	BB	BB	1	1	1	1	1	1
1519	CH17MM14	17	15215815		AA	AA	AA	AA	AA	AA	AA	AA	1	1	1	1	1	1
839	CH17MM15	17	16276466		BB	BB	BB	BB	BB	BB	BB	BB	1	1	1	1	1	1
972	CH17MM16	17	17846305		BB	BB	BB	BB	BB	BB	BB	BB	1	1	1	1	1	1
840	CH17MM17	17	19510335		BB	BB	BB	BB	BB	BB	BB	BB	1	1	1	1	1	1
1321	CH17MM18	17	21394398		AA	AA	AA	AA	AA	AA	AA	AA	1	1	1	1	1	1

1365	CH17MM19	17	22926002		BB	BB	BB	BB	BB	BB	BB	BB	1	1	1	1	1	1
1474	CH17MM20	17	24100880		BB	BB	BB	BB	BB	BB	BB	BB	1	1	1	1	1	1
308	CH17MM21	17	24799566		BB	BB	BB	BB	BB	BB	BB	BB	1	1	1	1	1	1
1961	CH17MM22	17	26039914		BB	BB	BB	BB	BB	BB	BB	BB	1	1	1	1	1	1
1298	CH17MM23	17	28381655		BB	BB	BB	BB	BB	BB	BB	BB	1	1	1	1	1	1
1937	CH17MM24GM1	17	28781038		AA	AA	AA	AA	AA	AA	AA	AA	1	1	1	1	1	1
1196	CH17MM25	17	31726297		BB	BB	BB	BB	BB	BB	BB	BB	1	1	1	1	1	1
1040	CH17MM26GM2	17	33807107		AA	AA	AA	AA	AA	AA	AA	AA	1	1	1	1	1	1
1245	CH17MM27	17	34343989		BB	BB	BB	BB	BB	BB	BB	BB	1	1	1	1	1	1
2005	CH17MM28	17	34795027		BB	BB	BB	BB	BB	BB	BB	BB	1	1	1	1	1	1
1851	CH17MM29	17	35059374		AA	AA	AA	AA	AA	AA	AA	AA	1	1	1	1	1	1
1986	CH17MM30	17	36989322		BB	BB	BB	BB	BB	BB	BB	BB	1	1	1	1	1	1
54	CH17MM31	17	37478448		AA	AA	AA	AA	AA	AA	AA	AA	1	1	1	1	1	1
1949	CH17MM32	17	38990980		AA	AA	AA	AA	AA	AA	AA	AA	1	1	1	1	1	1
55	CH17MM33	17	39772541		BB	BB	BB	BB	BB	BB	BB	BB	1	1	1	1	1	1
1622	CH17MM34	17	41235453		BB	BB	BB	BB	BB	BB	BB	BB	1	1	1	1	1	1
841	CH17MM35	17	41975280		BB	BB	BB	BB	BB	BB	BB	BB	1	1	1	1	1	1
1074	CH17MM36BLK2	17	43804134	X	AA	BB	BB	BB	BB	BB	BB	BB	1	1	1	1	1	1
842	CH17MM37	17	43897393		BB	BB	BB	BB	BB	BB	BB	BB	1	1	1	1	1	1
2045	CH17MM38	17	44325625		AA	AA	AA	AA	AA	AA	AA	AA	1	1	1	1	1	1
1978	CH17MM39	17	45506664		BB	BB	BB	BB	BB	BB	BB	BB	1	1	1	1	1	1
944	CH17MM40	17	46026842		AA	AA	AA	AA	AA	AA	AA	AA	1	1	1	1	1	1
843	CH17MM41	17	46772416		BB	BB	BB	BB	BB	BB	BB	BB	1	1	1	1	1	1
844	CH17MM42	17	47737011		AA	AA	AA	AA	AA	AA	AA	AA	1	1	1	1	1	1
1674	CH17MM43	17	48109331		AA	AA	AA	AA	AA	AA	AA	AA	1	1	1	1	1	1
1348	CH17MM44	17	50793087		AA	AA	AA	AA	AA	AA	AA	AA	1	1	1	1	1	1
1821	CH17MM45	17	51663908		BB	BB	BB	BB	BB	BB	BB	BB	1	1	1	1	1	1
1439	CH17MM46	17	53552802		AA	AA	AA	AA	AA	AA	AA	AA	1	1	1	1	1	1
1445	CH17MM47	17	56999568		BB	BB	BB	BB	BB	BB	BB	BB	1	1	1	1	1	1
213	CH17MM48	17	61107080		BB	BB	BB	BB	BB	BB	BB	BB	1	1	1	1	1	1
242	CH17MM49	17	61947366		AA	AA	AA	AA	AA	AA	AA	AA	1	1	1	1	1	1
1967	CH17MM50	17	62066360		AA	AA	AA	AA	AA	AA	AA	AA	1	1	1	1	1	1
309	CH17MM51	17	64224877		BB	BB	BB	BB	BB	BB	BB	BB	1	1	1	1	1	1
1522	CH17MM52	17	66156395		BB	BB	BB	BB	BB	BB	BB	BB	1	1	1	1	1	1
845	CH17MM53	17	66984514		BB	BB	BB	BB	BB	BB	BB	BB	1	1	1	1	1	1
1097	CH17MM54	17	67483047		AA	AA	AA	AA	AA	AA	AA	AA	1	1	1	1	1	1
9	CH17MM55GM3	17	68465978		BB	BB	BB	BB	BB	BB	BB	BB	1	1	1	1	1	1
1115	CH17MM56	17	69021924		BB	BB	BB	BB	BB	BB	BB	BB	1	1	1	1	1	1
1206	CH17MM57	17	69670995		BB	BB	BB	BB	BB	BB	BB	BB	1	1	1	1	1	1
1289	CH17MM58	17	70833240		BB	BB	BB	BB	BB	BB	BB	BB	1	1	1	1	1	1
846	CH17MM59	17	72668814		BB	BB	BB	BB	BB	BB	BB	BB	1	1	1	1	1	1
295	CH17MM60	17	73103736		BB	BB	BB	BB	BB	BB	BB	BB	1	1	1	1	1	1
1775	CH17MM61	17	73658199		BB	BB	BB	BB	BB	BB	BB	BB	1	1	1	1	1	1
847	CH17MM62	17	73981248		BB	BB	BB	BB	BB	BB	BB	BB	1	1	1	1	1	1
1137	CH17MM63	17	75526825		AA	AA	AA	AA	AA	AA	AA	AA	1	1	1	1	1	1
848	CH17MM64	17	77391928		AA	AA	AA	AA	AA	AA	AA	AA	1	1	1	1	1	1
1178	CH17MM65	17	77914489		BB	BB	BB	BB	BB	BB	BB	BB	1	1	1	1	1	1
214	CH17MM66	17	79515223		BB	BB	BB	BB	BB	BB	BB	BB	1	1	1	1	1	1
1662	CH17MM67	17	81015513		BB	BB	BB	BB	BB	BB	BB	BB	1	1	1	1	1	1
215	CH17MM68	17	81077502		BB	BB	BB	BB	BB	BB	BB	BB	1	1	1	1	1	1
849	CH17MM69	17	82051202		BB	BB	BB	BB	BB	BB	BB	BB	1	1	1	1	1	1
850	CH17MM70	17	84456436		BB	BB	BB	BB	BB	BB	BB	BB	1	1	1	1	1	1
1623	CH17MM71	17	85385338		AA	AA	AA	AA	AA	AA	AA	AA	1	1	1	1	1	1
310	CH17MM72	17	85857606		BB	BB	BB	BB	BB	BB	BB	BB	1	1	1	1	1	1
56	CH17MM73	17	86913143		BB	BB	BB	BB	BB	BB	BB	BB	1	1	1	1	1	1
851	CH17MM74	17	87503632		AA	AA	AA	AA	AA	AA	AA	AA	1	1	1	1	1	1
1696	CH17MM75	17	89199039		BB	BB	BB	BB	BB	BB	BB	BB	1	1	1	1	1	1
1839	CH17MM76	17	90215134		AA	AA	AA	AA	AA	AA	AA	AA	1	1	1	1	1	1
1960	CH17MM77	17	91938734		AA	AA	AA	AA	AA	AA	AA	AA	1	1	1	1	1	1
1380	CH17MM78	17	92912930		AA	AA	AA	AA	AA	AA	AA	AA	1	1	1	1	1	1
852	CH18MM1	18	3687056		AA	AA	AA	AA	AA	AA	AA	AA	1	1	1	1	1	1
853	CH18MM2	18	3967037		BB	BB	BB	BB	BB	BB	BB	BB	1	1	1	1	1	1
216	CH18MM3	18	4813500		BB	BB	BB	BB	BB	BB	BB	BB	1	1	1	1	1	1
58	CH18MM4	18	5565618		BB	BB	BB	BB	BB	BB	BB	BB	1	1	1	1	1	1
1282	CH18MM5	18	8254573		BB	BB	BB	BB	BB	BB	BB	BB	1	1	1	1	1	1
1117	CH18MM6	18	9491813		AA	AA	AA	AA	AA	AA	AA	AA	1	1	1	1	1	1
217	CH18MM7	18	10431432		AA	AA	AA	AA	AA	AA	AA	AA	1	1	1	1	1	1
1000	CH18MM8	18	11094899		AA	AA	AA	AA	AA	AA	AA	AA	1	1	1	1	1	1
1624	CH18MM9	18	11306822		AA	AA	AA	AA	AA	AA	AA	AA	1	1	1	1	1	1
2006	CH18MM10	18	12455434		BB	BB	BB	BB	BB	BB	BB	BB	1	1	1	1	1	1
854	CH18MM11	18	12477417		BB	BB	BB	BB	BB	BB	BB	BB	1	1	1	1	1	1
256	CH18MM12	18	14895705		AA	AA	AA	AA	AA	AA	AA	AA	1	1	1	1	1	1
1911	CH18MM13	18	15373312		AA	AA	AA	AA	AA	AA	AA	AA	1	1	1	1	1	1
1904	CH18MM14	18	16784949		AA	AA	AA	AA	AA	AA	AA	AA	1	1	1	1	1	1
855	CH18MM15BLK3	18	19671420	X	BB	AA	AA	AA	AA	AA	AA	AA	1	1	1	1	1	1
1008	CH18MM16	18	21109817		BB	BB	BB	BB	BB	BB	BB	BB	1	1	1	1	1	1
856	CH18MM17	18	21226461		AA	AA	AA	AA	AA	AA	AA	AA	1	1	1	1	1	1
1532	CH18MM18	18	23001325		BB	BB	BB	BB	BB	BB	BB	BB	1	1	1	1	1	1
218	CH18MM19	18	24860622		BB	BB	BB	BB	BB	BB	BB	BB	1	1	1	1	1	1
219	CH18MM20	18	25729264		AA	AA	AA	AA	AA	AA	AA	AA	1	1	1	1	1	1
857	CH18MM21	18	26881733		BB	BB	BB	BB	BB	BB	BB	BB	1	1	1	1	1	1
858	CH18MM22	18	29410281		AA	AA	AA	AA	AA	AA	AA	AA	1	1	1	1	1	1
1636	CH18MM23	18	30632368		AA	AA	AA	AA	AA	AA	AA	AA	1	1	1	1	1	1
859	CH18MM24GM1	18	30923554		AA	AA	AA	AA	AA	AA	AA	AA	1	1	1	1	1	1
1252	CH18MM25	18	30952210		BB	BB	BB	BB	BB	BB	BB	BB	1	1	1	1	1	1
1490	CH18MM26	18	31395543		BB	BB	BB	BB	BB	BB	BB	BB	1	1	1	1	1	1
1460	CH18MM27	18	33572164		BB	BB	BB	BB	BB	BB	BB	BB	1	1	1	1	1	1
1323	CH18MM28	18	33968545		BB	BB	BB	BB	BB	BB	BB	BB	1	1	1	1	1	1
1512	CH18MM29	18	34867922		AA	AA	AA	AA	AA	AA	AA	AA	1	1	1	1	1	1
860	CH18MM30	18	36563136		BB	BB	BB	BB	BB	BB	BB	BB	1	1	1	1	1	1
1152	CH18MM31	18	37737388		AA	AA	AA	AA	AA	AA	AA	AA	1	1	1	1	1	1
1343	CH18MM32	18	39756682		AA	AA	AA	AA	AA	AA	AA	AA	1	1	1	1	1	1
861	CH18MM33	18	41351739		AA	AA	AA	AA	AA	AA	AA	AA	1	1	1	1	1	1
1210	CH18MM34	18	43364740		BB	BB	BB	BB	BB	BB	BB	BB	1	1	1	1	1	1
1625	CH18MM35	18	44463060		BB	BB	BB	BB	BB	BB	BB	BB	1	1	1	1	1	1
57	CH18MM36	18	46082735		BB	BB	BB	BB	BB	BB	BB	BB	1	1	1	1	1	1
1722	CH18MM37	18	47884501		AA	AA	AA	AA	AA	AA	AA	AA	1	1	1	1	1	1
259	CH18MM38	18	49464960		AA	AA	AA	AA	AA	AA	AA	AA	1	1	1	1	1	1
1202	CH18MM39	18	51674308		BB	BB	BB	BB	BB	BB	BB	BB	1	1	1	1	1	1
220	CH18MM40	18	53592447		BB	BB	BB	BB	BB	BB	BB	BB	1	1	1	1	1	1
862	CH18MM41	18	54949689		AA	AA	AA	AA	AA	AA	AA	AA	1	1	1	1	1	1
1249	CH18MM42	18	55685229		AA	AA	AA	AA	AA	AA	AA	AA	1	1	1	1	1	1
1880	CH18MM43	18	58310005		BB	BB	BB	BB	BB	BB	BB	BB	1	1	1	1	1	1
1172	CH18MM44	18	58570705		BB	BB	BB	BB	BB	BB	BB	BB	1	1	1	1	1	1
59	CH18MM45	18	60214752		BB	BB	BB	BB	BB	BB	BB	BB	1	1	1	1	1	1
1985	CH18MM46	18	62695790		BB	BB	BB	BB	BB	BB	BB	BB	1	1	1	1	1	1
1626	CH18MM47	18	63022572		BB	BB	BB	BB	BB	BB	BB	BB	1	1	1	1	1	1
1480	CH18MM48	18	63808511		BB	BB	BB	BB	BB	BB	BB	BB	1	1	1	1	1	1
1276	CH18MM49	18	65035105		AA	AA	AA	AA	AA	AA	AA	AA	1	1	1	1	1	1
1872	CH18MM50	18	65694361		AA	AA	AA	AA	AA	AA	AA	AA	1	1	1	1	1	1
1288	CH18MM51	18	67000836		AA	AA	AA	AA	AA	AA	AA	AA	1	1	1	1	1	1
1891	CH18MM52	18	69040190		AA	AA	AA	AA	AA	AA	AA	AA	1	1	1	1	1	1
1102	CH18MM53	18	69187029		BB	BB	BB	BB	BB	BB	BB	BB	1	1	1	1	1	1
863	CH18MM54	18	70651591		BB	BB	BB	BB	BB	BB	BB	BB	1	1	1	1	1	1
1708	CH18MM55	18	71978261		AA	AA	AA	AA	AA	AA	AA	AA	1	1	1	1	1	1
864	CH18MM56	18	73957870		BB	BB	BB	BB	BB	BB	BB	BB	1	1	1	1	1	1
1627	CH18MM57	18	74800133		BB	BB	BB	BB	BB	BB	BB	BB	1	1	1	1	1	1
315	CH18MM58	18	75265583		AA	AA	AA	AA	AA	AA	AA	AA	1	1	1	1	1	1
865	CH18MM59	18	77080840		AA	AA	AA	AA	AA	AA	AA	AA	1	1	1	1	1	1
1381	CH18MM60	18	77890660		AA	AA	AA	AA	AA	AA	AA	AA	1	1	1	1	1	1
1481	CH18MM61	18	80361633		AA	AA	AA	AA	AA	AA	AA	AA	1	1	1	1	1	1
7	CH18MM62GM2	18	81201346		AA	AA	AA	AA	AA	AA	AA	AA	1	1	1	1	1	1
866	CH18MM63	18	81619936		BB	BB	BB	BB	BB	BB	BB	BB	1	1	1	1	1	1
867	CH18MM64	18	82712912		BB	BB	BB	BB	BB	BB	BB	BB	1	1	1	1	1	1
21	CH18MM65BLK9	18	84686237	X	BB	AA	AA	AA	AA	AA	AA	AA	1	1	1	1	1	1
271	CH18MM66	18	86034550		AA	AA	AA	AA	AA	AA	AA	AA	1	1	1	1	1	1
868	CH18MM67	18	86617418		BB	BB	BB	BB	BB	BB	BB	BB	1	1	1	1	1	1

1528	CH18MM68	18	89120861		AA	AA	AA	AA	AA	AA	AA	AA	1	1	1	1	1	1
1184	CH19MM1	19	4208173		BB	BB	BB	BB	BB	BB	BB	BB	1	1	1	1	1	1
1432	CH19MM2GM1	19	4818261		BB	BB	BB	BB	BB	BB	BB	BB	1	1	1	1	1	1
63	CH19MM3	19	5283144		AA	AA	AA	AA	AA	AA	AA	AA	1	1	1	1	1	1
1780	CH19MM4	19	6000186		AA	AA	AA	AA	AA	AA	AA	AA	1	1	1	1	1	1
22	CH19MM5	19	9101495	X	AA	BB	BB	BB	BB	BB	BB	BB	1	1	1	1	1	1
869	CH19MM6	19	9543124		BB	BB	BB	BB	BB	BB	BB	BB	1	1	1	1	1	1
2046	CH19MM7	19	9813271		AA	AA	AA	AA	AA	AA	AA	AA	1	1	1	1	1	1
1370	CH19MM8	19	10064010		BB	BB	BB	BB	BB	BB	BB	BB	1	1	1	1	1	1
1047	CH19MM9	19	10066261		BB	BB	BB	BB	BB	BB	BB	BB	1	1	1	1	1	1
1044	CH19MM10	19	10088937		BB	BB	BB	BB	BB	BB	BB	BB	1	1	1	1	1	1
1868	CH19MM11	19	10572580		BB	BB	BB	BB	BB	BB	BB	BB	1	1	1	1	1	1
60	CH19MM12	19	12595293		AA	AA	AA	AA	AA	AA	AA	AA	1	1	1	1	1	1
870	CH19MM13	19	14743248		BB	BB	BB	BB	BB	BB	BB	BB	1	1	1	1	1	1
1266	CH19MM14	19	16159229		AA	AA	AA	AA	AA	AA	AA	AA	1	1	1	1	1	1
1766	CH19MM15	19	17804343		BB	BB	BB	BB	BB	BB	BB	BB	1	1	1	1	1	1
221	CH19MM16	19	19283950		AA	AA	AA	AA	AA	AA	AA	AA	1	1	1	1	1	1
1921	CH19MM17	19	21112530		AA	AA	AA	AA	AA	AA	AA	AA	1	1	1	1	1	1
1858	CH19MM18	19	22859563		BB	BB	BB	BB	BB	BB	BB	BB	1	1	1	1	1	1
1482	CH19MM19	19	24396755		AA	AA	AA	AA	AA	AA	AA	AA	1	1	1	1	1	1
1848	CH19MM20	19	25362165		BB	BB	BB	BB	BB	BB	BB	BB	1	1	1	1	1	1
1171	CH19MM21	19	25643608		AA	AA	AA	AA	AA	AA	AA	AA	1	1	1	1	1	1
1056	CH19MM22	19	26731527		AA	AA	AA	AA	AA	AA	AA	AA	1	1	1	1	1	1
2047	CH19MM23	19	29540192		BB	BB	BB	BB	BB	BB	BB	BB	1	1	1	1	1	1
871	CH19MM24GM2	19	30116026		BB	BB	BB	BB	BB	BB	BB	BB	1	1	1	1	1	1
316	CH19MM25	19	31358105		BB	BB	BB	BB	BB	BB	BB	BB	1	1	1	1	1	1
1013	CH19MM26	19	31484461		AA	AA	AA	AA	AA	AA	AA	AA	1	1	1	1	1	1
1781	CH19MM27	19	31672951		BB	BB	BB	BB	BB	BB	BB	BB	1	1	1	1	1	1
61	CH19MM28	19	32349880		AA	AA	AA	AA	AA	AA	AA	AA	1	1	1	1	1	1
62	CH19MM29	19	34542259		BB	BB	BB	BB	BB	BB	BB	BB	1	1	1	1	1	1
222	CH19MM30	19	36467112		BB	BB	BB	BB	BB	BB	BB	BB	1	1	1	1	1	1
1415	CH19MM31	19	37393165		BB	BB	BB	BB	BB	BB	BB	BB	1	1	1	1	1	1
1996	CH19MM32	19	39330470		BB	BB	BB	BB	BB	BB	BB	BB	1	1	1	1	1	1
1087	CH19MM33	19	40259657		AA	AA	AA	AA	AA	AA	AA	AA	1	1	1	1	1	1
1006	CH19MM34BLK4	19	40364531	X	AA	BB	BB	BB	BB	BB	BB	BB	1	1	1	1	1	1
872	CH19MM35	19	40619480		BB	BB	BB	BB	BB	BB	BB	BB	1	1	1	1	1	1
1094	CH19MM36	19	41749395		BB	BB	BB	BB	BB	BB	BB	BB	1	1	1	1	1	1
1269	CH19MM37	19	42406846		BB	BB	BB	BB	BB	BB	BB	BB	1	1	1	1	1	1
873	CH19MM38	19	44651448		BB	BB	BB	BB	BB	BB	BB	BB	1	1	1	1	1	1
874	CH19MM39	19	45118329		BB	BB	BB	BB	BB	BB	BB	BB	1	1	1	1	1	1
252	CH19MM40	19	45356002		AA	AA	AA	AA	AA	AA	AA	AA	1	1	1	1	1	1
875	CH19MM41	19	46717958		AA	AA	AA	AA	AA	AA	AA	AA	1	1	1	1	1	1
1041	CH19MM42	19	47482330		BB	BB	BB	BB	BB	BB	BB	BB	1	1	1	1	1	1
1735	CH19MM43	19	49475053		BB	BB	BB	BB	BB	BB	BB	BB	1	1	1	1	1	1
248	CH19MM44	19	52162185		BB	BB	BB	BB	BB	BB	BB	BB	1	1	1	1	1	1
1856	CH19MM45	19	52797648		AA	AA	AA	AA	AA	AA	AA	AA	1	1	1	1	1	1
876	CH19MM46	19	54535312		AA	AA	AA	AA	AA	AA	AA	AA	1	1	1	1	1	1
1754	CH19MM47	19	56380821		AA	AA	AA	AA	AA	AA	AA	AA	1	1	1	1	1	1
1732	CH19MM48	19	58720409		AA	AA	AA	AA	AA	AA	AA	AA	1	1	1	1	1	1
2050	CH19MM49	19	59475231		AA	AA	AA	AA	AA	AA	AA	AA	1	1	1	1	1	1
8	CH19MM50GM3	19	60149305		BB	BB	BB	BB	BB	BB	BB	BB	1	1	1	1	1	1
1011	CHXMM1	X	6775675		BB	BB	BB	BB	BB	BB	BB	BB	1	1	1	1	1	1
877	CHXMM2	X	7885141		AA	AA	AA	AA	AA	AA	AA	AA	1	1	1	1	1	1
1999	CHXMM3	X	7947021		BB	BB	BB	BB	BB	BB	BB	BB	1	1	1	1	1	1
131	CHXMM4	X	8334947		AA	AA	AA	AA	AA	AA	AA	AA	1	1	1	1	1	1
132	CHXMM5	X	8498891		AA	AA	AA	AA	AA	AA	AA	AA	1	1	1	1	1	1
878	CHXMM6	X	8776871		BB	BB	BB	BB	BB	BB	BB	BB	1	1	1	1	1	1
134	CHXMM7	X	9031623		BB	BB	BB	BB	BB	BB	BB	BB	1	1	1	1	1	1
894	CHXMM8	X	10762134		AA	AA	AA	AA	AA	AA	AA	AA	1	1	1	1	1	1
879	CHXMM9GM1	X	28552142		BB	BB	BB	BB	BB	BB	BB	BB	1	1	1	1	1	1
223	CHXMM10	X	31247994		AA	AA	AA	AA	AA	AA	AA	AA	1	1	1	1	1	1
224	CHXMM11	X	34572975		AA	AA	AA	AA	AA	AA	AA	AA	1	1	1	1	1	1
880	CHXMM12	X	34801132		AA	AA	AA	AA	AA	AA	AA	AA	1	1	1	1	1	1
120	CHXMM13	X	39961894		BB	BB	BB	BB	BB	BB	BB	BB	1	1	1	1	1	1
881	CHXMM14	X	40510546		AA	AA	AA	AA	AA	AA	AA	AA	1	1	1	1	1	1
882	CHXMM15	X	42770100		AA	AA	AA	AA	AA	AA	AA	AA	1	1	1	1	1	1
225	CHXMM16	X	43278349		AA	AA	AA	AA	AA	AA	AA	AA	1	1	1	1	1	1
883	CHXMM17	X	43720683		BB	BB	BB	BB	BB	BB	BB	BB	1	1	1	1	1	1
121	CHXMM18	X	44311522		BB	BB	BB	BB	BB	BB	BB	BB	1	1	1	1	1	1
1981	CHXMM19	X	44405254		AA	AA	AA	AA	AA	AA	AA	AA	1	1	1	1	1	1
884	CHXMM20	X	45475262		BB	BB	BB	BB	BB	BB	BB	BB	1	1	1	1	1	1
885	CHXMM21	X	47895370		AA	AA	AA	AA	AA	AA	AA	AA	1	1	1	1	1	1
137	CHXMM22BLK3	X	47904667	X	BB	AA	AA	AA	AA	AA	AA	AA	1	1	1	1	1	1
886	CHXMM23	X	49186978		BB	BB	BB	BB	BB	BB	BB	BB	1	1	1	1	1	1
226	CHXMM24	X	52389338		BB	BB	BB	BB	BB	BB	BB	BB	1	1	1	1	1	1
887	CHXMM25	X	52741694		BB	BB	BB	BB	BB	BB	BB	BB	1	1	1	1	1	1
888	CHXMM26	X	53942181		AA	AA	AA	AA	AA	AA	AA	AA	1	1	1	1	1	1
889	CHXMM27	X	55210782		AA	AA	AA	AA	AA	AA	AA	AA	1	1	1	1	1	1
890	CHXMM28	X	56181989		AA	AA	AA	AA	AA	AA	AA	AA	1	1	1	1	1	1
122	CHXMM29	X	59515625		AA	AA	AA	AA	AA	AA	AA	AA	1	1	1	1	1	1
123	CHXMM30	X	60181392		BB	BB	BB	BB	BB	BB	BB	BB	1	1	1	1	1	1
290	CHXMM31	X	61228987		AA	AA	AA	AA	AA	AA	AA	AA	1	1	1	1	1	1
1019	CHXMM32	X	62517350		BB	BB	BB	BB	BB	BB	BB	BB	1	1	1	1	1	1
1063	CHXMM33	X	63007260		AA	AA	AA	AA	AA	AA	AA	AA	1	1	1	1	1	1
892	CHXMM34	X	65765926		BB	BB	BB	BB	BB	BB	BB	BB	1	1	1	1	1	1
124	CHXMM35	X	66015326		AA	AA	AA	AA	AA	AA	AA	AA	1	1	1	1	1	1
893	CHXMM36	X	67285372		AA	AA	AA	AA	AA	AA	AA	AA	1	1	1	1	1	1
125	CHXMM37	X	67980997		AA	AA	AA	AA	AA	AA	AA	AA	1	1	1	1	1	1
126	CHXMM38	X	68179178		AA	AA	AA	AA	AA	AA	AA	AA	1	1	1	1	1	1
1998	CHXMM39	X	68698000		AA	AA	AA	AA	AA	AA	AA	AA	1	1	1	1	1	1
227	CHXMM40	X	72274499		BB	BB	BB	BB	BB	BB	BB	BB	1	1	1	1	1	1
127	CHXMM41	X	72627341		BB	BB	BB	BB	BB	BB	BB	BB	1	1	1	1	1	1
128	CHXMM42	X	72875547		AA	AA	AA	AA	AA	AA	AA	AA	1	1	1	1	1	1
895	CHXMM43	X	73756646		AA	AA	AA	AA	AA	AA	AA	AA	1	1	1	1	1	1
896	CHXMM44	X	74580193		BB	BB	BB	BB	BB	BB	BB	BB	1	1	1	1	1	1
129	CHXMM45	X	75125049		BB	BB	BB	BB	BB	BB	BB	BB	1	1	1	1	1	1
897	CHXMM46	X	76146750		BB	BB	BB	BB	BB	BB	BB	BB	1	1	1	1	1	1
898	CHXMM47	X	76868167		AA	AA	AA	AA	AA	AA	AA	AA	1	1	1	1	1	1
1513	CHXMM48	X	77048128		AA	AA	AA	AA	AA	AA	AA	AA	1	1	1	1	1	1
130	CHXMM49	X	77780392		AA	AA	AA	AA	AA	AA	AA	AA	1	1	1	1	1	1
899	CHXMM50	X	78499387		AA	AA	AA	AA	AA	AA	AA	AA	1	1	1	1	1	1
891	CHXMM51	X	81745577		AA	AA	AA	AA	AA	AA	AA	AA	1	1	1	1	1	1
228	CHXMM52	X	84227028		AA	AA	AA	AA	AA	AA	AA	AA	1	1	1	1	1	1
900	CHXMM53	X	85456399		AA	AA	AA	AA	AA	AA	AA	AA	1	1	1	1	1	1
313	CHXMM54	X	86087663		BB	BB	BB	BB	BB	BB	BB	BB	1	1	1	1	1	1
229	CHXMM55	X	87586756		BB	BB	BB	BB	BB	BB	BB	BB	1	1	1	1	1	1
901	CHXMM56	X	87902633		AA	AA	AA	AA	AA	AA	AA	AA	1	1	1	1	1	1
1720	CHXMM57	X	88379974		BB	BB	BB	BB	BB	BB	BB	BB	1	1	1	1	1	1
133	CHXMM58	X	89752158		BB	BB	BB	BB	BB	BB	BB	BB	1	1	1	1	1	1
135	CHXMM59	X	91222960		BB	BB	BB	BB	BB	BB	BB	BB	1	1	1	1	1	1
902	CHXMM60	X	91823100		BB	BB	BB	BB	BB	BB	BB	BB	1	1	1	1	1	1
230	CHXMM61	X	92235622		BB	BB	BB	BB	BB	BB	BB	BB	1	1	1	1	1	1
231	CHXMM62	X	93530235		AA	AA	AA	AA	AA	AA	AA	AA	1	1	1	1	1	1
903	CHXMM63	X	95063466		BB	BB	BB	BB	BB	BB	BB	BB	1	1	1	1	1	1
232	CHXMM64	X	97261992		BB	BB	BB	BB	BB	BB	BB	BB	1	1	1	1	1	1
904	CHXMM65	X	97492766		AA	AA	AA	AA	AA	AA	AA	AA	1	1	1	1	1	1
905	CHXMM66	X	97613903		AA	AA	AA	AA	AA	AA	AA	AA	1	1	1	1	1	1
1042	CHXMM67	X	98148521		BB	BB	BB	BB	BB	BB	BB	BB	1	1	1	1	1	1
906	CHXMM68	X	98226460		BB	BB	BB	BB	BB	BB	BB	BB	1	1	1	1	1	1
107	CHXMM69	X	100299803		BB	BB	BB	BB	BB	BB	BB	BB	1	1	1	1	1	1
108	CHXMM70	X	100430565		BB	BB	BB	BB	BB	BB	BB	BB	1	1	1	1	1	1
2036	CHXMM71	X	100641829		BB	BB	BB	BB	BB	BB	BB	BB	1	1	1	1	1	1
2037	CHXMM72	X	100679982		AA	AA	AA	AA	AA	AA	AA	AA	1	1	1	1	1	1
907	CHXMM73	X	102010268		BB	BB	BB	BB	BB	BB	BB	BB	1	1	1	1	1	1
908	CHXMM74	X	102845364		BB	BB	BB	BB	BB	BB	BB	BB	1	1	1	1	1	1
1889	CHXMM75	X	103875234		BB	BB	BB	BB	BB	BB	BB	BB	1	1	1	1	1	1
2024	CHXMM76	X	104300680		BB	BB	BB	BB	BB	BB	BB	BB	1	1	1	1	1	1

109	CHXMM77	X	106858553	BB	BB	BB	BB	BB	BB	BB	BB	1	1	1	1	1	1
1913	CHXMM78	X	109582439	AA	AA	AA	AA	AA	AA	AA	AA	1	1	1	1	1	1
909	CHXMM79	X	111135758	AA	AA	AA	AA	AA	AA	AA	AA	1	1	1	1	1	1
110	CHXMM80	X	112457192	AA	AA	AA	AA	AA	AA	AA	AA	1	1	1	1	1	1
111	CHXMM81	X	112904656	AA	AA	AA	AA	AA	AA	AA	AA	1	1	1	1	1	1
141	CHXMM82	X	116527625	BB	BB	BB	BB	BB	BB	BB	BB	1	1	1	1	1	1
112	CHXMM83	X	117683749	BB	BB	BB	BB	BB	BB	BB	BB	1	1	1	1	1	1
1023	CHXMM84GM2	X	119054479	BB	BB	BB	BB	BB	BB	BB	BB	1	1	1	1	1	1
1747	CHXMM85	X	120300412	AA	AA	AA	AA	AA	AA	AA	AA	1	1	1	1	1	1
1762	CHXMM86	X	121042456	AA	AA	AA	AA	AA	AA	AA	AA	1	1	1	1	1	1
910	CHXMM87	X	121285657	AA	AA	AA	AA	AA	AA	AA	AA	1	1	1	1	1	1
911	CHXMM88	X	122120659	BB	BB	BB	BB	BB	BB	BB	BB	1	1	1	1	1	1
912	CHXMM89	X	123782683	AA	AA	AA	AA	AA	AA	AA	AA	1	1	1	1	1	1
1362	CHXMM90	X	124956381	AA	AA	AA	AA	AA	AA	AA	AA	1	1	1	1	1	1
113	CHXMM91	X	125736335	BB	BB	BB	BB	BB	BB	BB	BB	1	1	1	1	1	1
1325	CHXMM92	X	126223821	BB	BB	BB	BB	BB	BB	BB	BB	1	1	1	1	1	1
913	CHXMM93	X	128014181	BB	BB	BB	BB	BB	BB	BB	BB	1	1	1	1	1	1
914	CHXMM94	X	129847872	AA	AA	AA	AA	AA	AA	AA	AA	1	1	1	1	1	1
915	CHXMM95	X	130685594	BB	BB	BB	BB	BB	BB	BB	BB	1	1	1	1	1	1
916	CHXMM96	X	132280571	AA	AA	AA	AA	AA	AA	AA	AA	1	1	1	1	1	1
917	CHXMM97	X	132699243	AA	AA	AA	AA	AA	AA	AA	AA	1	1	1	1	1	1
114	CHXMM98	X	133525088	BB	BB	BB	BB	BB	BB	BB	BB	1	1	1	1	1	1
918	CHXMM99	X	134426687	BB	BB	BB	BB	BB	BB	BB	BB	1	1	1	1	1	1
2009	CHXMM100	X	134595379	AA	AA	AA	AA	AA	AA	AA	AA	1	1	1	1	1	1
919	CHXMM101	X	136857358	AA	AA	AA	AA	AA	AA	AA	AA	1	1	1	1	1	1
920	CHXMM102	X	140595720	AA	AA	AA	AA	AA	AA	AA	AA	1	1	1	1	1	1
115	CHXMM103	X	142652525	BB	BB	BB	BB	BB	BB	BB	BB	1	1	1	1	1	1
116	CHXMM104	X	143438541	AA	AA	AA	AA	AA	AA	AA	AA	1	1	1	1	1	1
117	CHXMM105	X	143595976	AA	AA	AA	AA	AA	AA	AA	AA	1	1	1	1	1	1
921	CHXMM106	X	145863727	BB	BB	BB	BB	BB	BB	BB	BB	1	1	1	1	1	1
922	CHXMM107	X	147274623	AA	AA	AA	AA	AA	AA	AA	AA	1	1	1	1	1	1
1914	CHXMM108	X	150148872	AA	AA	AA	AA	AA	AA	AA	AA	1	1	1	1	1	1
1656	CHXMM109	X	151368132	BB	BB	BB	BB	BB	BB	BB	BB	1	1	1	1	1	1
233	CHXMM110	X	151555476	AA	AA	AA	AA	AA	AA	AA	AA	1	1	1	1	1	1
118	CHXMM111	X	154048891	AA	AA	AA	AA	AA	AA	AA	AA	1	1	1	1	1	1
119	CHXMM112	X	155542834	AA	AA	AA	AA	AA	AA	AA	AA	1	1	1	1	1	1
234	CHXMM113	X	156799296	AA	AA	AA	AA	AA	AA	AA	AA	1	1	1	1	1	1
235	CHXMM114	X	158940488	BB	BB	BB	BB	BB	BB	BB	BB	1	1	1	1	1	1
923	CHXMM115GM3	X	159567431	AA	AA	AA	AA	AA	AA	AA	AA	1	1	1	1	1	1
236	CHYMM1BLK1GM1	Y	1362900	BB	BB	BB	NC	BB	NC	BB	NC	1	NA	1	NA	1	NA

**SNP's**	2040,5	2040	2037,5	2042,5	2040,5	2035
**Total SNPs**	2052	2051	2052	2051	2052	2051
**% C57BL/6J**	99,44%	99,46%	99,29%	99,59%	99,44%	99,22%

Supplementary Tables

Supplementary Table 2. Antibodies used for flow cytometry

Antigen	Fluorophore	Clone	Catalog N°	Company
**Lymphoid panel (100 000 cells read)**				
CD3	FITC	145-2C11	11-0031-81	eBioscience
CD8	PerCP-Cy5.5	53-6.7	45-0081-82	eBioscience
CD183 (CXCR3)	PE	CXCR3-173	126505	Biolegend
CD44	PE	IM7	103007	Biolegend
NK1.1	PE-Cy7	PK136	25-5941-81	eBioscience
CD4	APC-eFluor 780	RM4-5	47-0042-80	eBioscience
CD49b (DX5)	APC	DX5	17-5971-81	eBioscience
CD62L	APC	MEL-14	17-0621-83	eBioscience
B220	BV786	RA3-6B2	563894	BD Biosciences
CD45	BUV395	30-F11	564279	BD Biosciences
Live/dead	Zombie Aqua™ Fixable Viability Kit		423101	Biolegend
**Myeloid panel (200 000 cells read)**				
CD45	FITC	30-F11	103107	Biolegend
CD103	PerCP-eFluor 710	2E7	46-1031-80	eBioscience
CD11c	PE-Cy7	N418	117318	Biolegend
CD64	PE	X54-5/7.1	139303	Biolegend
F4/80	PE	BM8	12-4801-82	eBioscience
CD24	PE-CF594	M1/69	101837	Biolegend
SiglecF	eFluor 660	1RNM44N	50-1702-80	eBioscience
Ly6G	Alexa fluor 700	1A8	561236	BD Biosciences
Ly6C	APC-Cy7	AL-21	560596	BD Biosciences
CD11b	eFluor 450	M1/70	48-0112-80	eBioscience
MHC-II	Horizon v500	M5/114	562366	BD Biosciences

CD3	BV650	17A2	100229	Biolegend
CD19	BV650	6D5	115541	Biolegend
NK1.1	BV650	PK136	108736	Biolegend
Live/dead	Zombie UV™ Fixable Viability Kit		423107	Biolegend

Supplementary Table 3. Primers used for RT-qPCR

**Gene**	**Full protein name**	**Exon location**	**Fluorescent dye**	**Catalog number primer**
18S	18S ribosomal RNA	1-1	FAM™	Hs.PT.39a.22214856.g
Ifng	Interferon gamma	1-2	FAM™	Mm.PT.58.41769240
Il10	Interleukin 10	1-3	FAM™	Mm.PT.58.23604055
Gzmb	Granzyme B	1-2	FAM™	Mm.PT.58.42155916
Prf1	Perforin	2-3	FAM™	Mm.PT.58.41904164
Tgfb1	Transforming growth factor beta	1-2	FAM™	Mm.PT.58.11254750
Ccl5	CC chemokine ligand 5 (RANTES)	2-3	FAM™	Mm.PT.58.43548565
Xcl1	C Chemokine 1 (Lymphotactin)	1-2	FAM™	Mm.PT.58.10915849
